# Bi-allelic loss-of-function variants in *TMEM147* cause moderate to profound intellectual disability with facial dysmorphism and pseudo-Pelger-Huët anomaly

**DOI:** 10.1016/j.ajhg.2022.08.008

**Published:** 2022-08-30

**Authors:** Quentin Thomas, Marialetizia Motta, Thierry Gautier, Maha S. Zaki, Andrea Ciolfi, Julien Paccaud, François Girodon, Odile Boespflug-Tanguy, Thomas Besnard, Jennifer Kerkhof, Haley McConkey, Aymeric Masson, Anne-Sophie Denommé-Pichon, Benjamin Cogné, Eva Trochu, Virginie Vignard, Fatima El It, Lance H. Rodan, Mohammad Ayman Alkhateeb, Rami Abou Jamra, Laurence Duplomb, Emilie Tisserant, Yannis Duffourd, Ange-Line Bruel, Adam Jackson, Siddharth Banka, Meriel McEntagart, Anand Saggar, Joseph G. Gleeson, David Sievert, Hyunwoo Bae, Beom Hee Lee, Kisang Kwon, Go Hun Seo, Hane Lee, Anjum Saeed, Nadeem Anjum, Huma Cheema, Salem Alawbathani, Imran Khan, Jorge Pinto-Basto, Joyce Teoh, Jasmine Wong, Umar Bin Mohamad Sahari, Henry Houlden, Kristina Zhelcheska, Melanie Pannetier, Mona A. Awad, Marion Lesieur-Sebellin, Giulia Barcia, Jeanne Amiel, Julian Delanne, Christophe Philippe, Laurence Faivre, Sylvie Odent, Aida Bertoli-Avella, Christel Thauvin, Bekim Sadikovic, Bruno Reversade, Reza Maroofian, Jérôme Govin, Marco Tartaglia, Antonio Vitobello

**Affiliations:** 1 UMR1231 GAD, Inserm, Université Bourgogne-Franche Comté, Dijon, France; 2 Genetics and Rare Diseases Research Division, Ospedale Pediatrico Bambino Gesù, IRCCS, 00146 Rome, Italy; 3 University Grenoble Alpes, Inserm, CNRS, Institute for Advanced Biosciences, 38000 Grenoble, France; 4 Clinical Genetics Department, Human Genetics and Genome Research Institute, National Research Centre, Cairo, Egypt; 5 Armed Forces College of Medicine, Cairo, Egypt; 6 Biology Division, Department of Biological Hematology, Dijon Hospital, 21000 Dijon, France; 7 Université Paris Cité, UMR 1141 NeuroDiderot, Inserm, 75019 Paris, France; 8 Service de Neuropédiatrie, reference center for leukodystrophies, APHP, Hopital Robert Debré, 75019 Paris, France; 9 Service de Génétique Médicale, CHU Nantes, Nantes, France; 10 Université de Nantes, CHU Nantes, CNRS, Inserm, l'Institut du Thorax, 44000 Nantes, France; 11 Verspeeten Clinical Genome Centre, London Health Sciences Centre, London, ON N6A 5W9, Canada; 12 Department of Pathology and Laboratory Medicine, Western University, London, ON N6A 3K7, Canada; 13 Unité Fonctionnelle Innovation en Diagnostic Génomique des Maladies Rares, FHU-TRANSLAD, CHU Dijon Bourgogne, Dijon, France; 14 Division of Genetics and Genomics, Boston Children’s Hospital, Harvard Medical School, Boston, MA 02115, USA; 15 Department of Neurology, Boston Children’s Hospital, Harvard Medical School, Boston, MA 02115, USA; 16 Women Wellness and Research Center Hamad Medical Corporation, Doha, Qatar; 17 Institute of Human Genetics, University Medical Center, Leipzig, Germany; 18 Division of Evolution, Infection and Genomics, School of Biological Sciences, Faculty of Biology, Medicine and Health, University of Manchester, Manchester, UK; 19 Manchester Centre for Genomic Medicine, St Mary’s Hospital, Manchester University NHS Foundation Trust, Health Innovation Manchester, Manchester, UK; 20 Medical Genetics, St George’s University Hospitals NHS FT, London SW17 0RE, UK; 21 The Portland Hospital, 205-209 Great Portland St, London W1W 5AH, UK; 22 Department of Neurosciences, University of California, San Diego, La Jolla, CA 92093, USA; 23 Rady Children’s Institute for Genomic Medicine, San Diego, La Jolla, CA 92093, USA; 24 Department of Pediatrics, Asan Medical Center Children’s Hospital, University of Ulsan College of Medicine, Seoul, Republic of Korea; 25 3billion, Inc, Seoul, South Korea; 26 Children’s Hospital and University of Child Health Lahore, Lahore, Pakistan; 27 CENTOGENE GmbH, 18055 Rostock, Germany; 28 Laboratory of Human Genetics & Therapeutics, Genome Institute of Singapore, A^∗^STAR, Singapore, Singapore; 29 Department of Neuromuscular Disease, UCL Queen Square Institute of Neurology and The National Hospital for Neurology and Neurosurgery, London, UK; 30 Service d’Hématologie cellulaire et hémostase bioclinique, CHU Rennes, Rennes, France; 31 Clinical and Chemical Pathology Department, Medical Research and Clinical Studies Institute National Research Centre, Cairo, Egypt; 32 Service de Médecine Génomique des Maladies Rares, Hôpital Necker-Enfant Malades, AP-HP, Paris, France; 33 Centre de Référence maladies rares « Anomalies du Développement et syndromes malformatifs », Centre de Génétique, FHU-TRANSLAD, CHU Dijon Bourgogne, Dijon, France; 34 Service de Génétique Clinique, Centre Référence Anomalies du Développement CLAD Ouest, Univ Rennes, Rennes, France; 35 Institut de Génétique et Développement de Rennes, CNRS Inserm UMR 6290, ERL 1305, Univ Rennes, Rennes, France; 36 Centre de référence maladies rares « déficiences intellectuelles de causes rares », Centre de Génétique, FHU-TRANSLAD, CHU Dijon Bourgogne, Dijon, France; 37 Medical Genetics Department, School of Medicine, Koç University, Istanbul, Turkey; 38 Smart-Health Initiative, King Abdullah University of Science and Technology, Thuwal, Saudi Arabia

**Keywords:** TMEM147, LBR, nuclear envelope instability, Pelger-Huët anomaly, translocon dysfunction, neurodevelopmental disorder, intellectual disability, facial dysmorphism, DNA methylation, transcriptomics

## Abstract

The transmembrane protein TMEM147 has a dual function: first at the nuclear envelope, where it anchors lamin B receptor (LBR) to the inner membrane, and second at the endoplasmic reticulum (ER), where it facilitates the translation of nascent polypeptides within the ribosome-bound TMCO1 translocon complex. Through international data sharing, we identified 23 individuals from 15 unrelated families with bi-allelic *TMEM147* loss-of-function variants, including splice-site, nonsense, frameshift, and missense variants. These affected children displayed congruent clinical features including coarse facies, developmental delay, intellectual disability, and behavioral problems. *In silico* structural analyses predicted disruptive consequences of the identified amino acid substitutions on translocon complex assembly and/or function, and *in vitro* analyses documented accelerated protein degradation via the autophagy-lysosomal-mediated pathway. Furthermore, TMEM147-deficient cells showed CKAP4 (CLIMP-63) and RTN4 (NOGO) upregulation with a concomitant reorientation of the ER, which was also witnessed in primary fibroblast cell culture. LBR mislocalization and nuclear segmentation was observed in primary fibroblast cells. Abnormal nuclear segmentation and chromatin compaction were also observed in approximately 20% of neutrophils, indicating the presence of a pseudo-Pelger-Huët anomaly. Finally, co-expression analysis revealed significant correlation with neurodevelopmental genes in the brain, further supporting a role of TMEM147 in neurodevelopment. Our findings provide clinical, genetic, and functional evidence that bi-allelic loss-of-function variants in *TMEM147* cause syndromic intellectual disability due to ER-translocon and nuclear organization dysfunction.

## Graphical abstract

**Figure:**
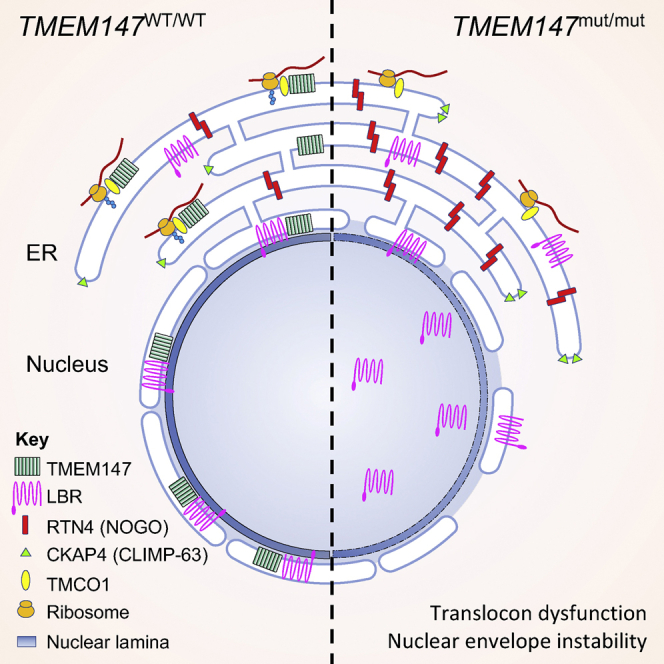

## Teaser

TMEM147 plays an important role in protein localization and biogenesis. We discovered that individuals with bi-allelic *TMEM147* loss-of-function variants show a neurodevelopmental disorder associated with facial dysmorphism and pseudo-Pelger-Huët anomaly. In primary cell lines, we observed nuclear envelope instability accompanied by lamin B receptor mislocalization and ER-translocon dysfunction.

## Main text

Intellectual disability (ID) is defined as a significant impairment in intellectual and adaptive functioning with onset during the developmental period and encompasses a wide panel of neurodevelopmental disorders of variable severity and presentation.^1^ With the growing use of massively parallel sequencing technologies, hundreds of genes have recently been linked to ID (either syndromic or not), thus involving countless molecular pathways in its pathophysiology.^2^, ^3^, ^4^ Yet, despite this incredible leap in recent knowledge, many affected individuals still remain without definite molecular diagnosis and adequate genetic counseling. This observation supports the fact that many more ID-associated genes are still to be discovered.

*TMEM147* (MIM: 613585), located at 19q13.12, encodes the ubiquitously expressed 224 amino acid transmembrane protein 147, an integral membrane protein with seven transmembrane domains.^5^ This protein is highly conserved in the animal kingdom with, respectively, 99% and 78% amino acid identity with mouse’s and zebrafish’s orthologs.^6^ Very little is currently known about TMEM147’s function. Previous studies using immunohistochemical analyses, co-immunoprecipitation assays, and yeast two-hybrid approach showed that TMEM147 is located in the membrane of the endoplasmic reticulum (ER) and that it regulates activity of many other proteins.^5^^,^^6^ Among these are the nicalin (encoded by *NCLN* [MIM: 609156]) and NODAL modulator 2 (NOMO, encoded by *NOMO2* [MIM: 609158]), two other transmembrane proteins of the ER of which TMEM147 is a major binding partner, or cholinergic receptor muscarinic 3 (encoded by *CHRM3* [MIM: 118494]).^5^^,^^6^ In particular, nicalin and NOMO have been described as antagonists of Nodal signaling during mesodermal patterning in zebrafish.^7^ Nicalin is related to nicastrin, one of the γ-secretase proteins involved in Alzheimer’s disease.^7^^,^^8^

A recent study identified a 360 kDa ribosome-associated complex comprising the core Sec61 (translocon) channel and the five accessory factors, transmembrane and coiled-coil domains 1 (TMCO1), coiled-coil domain-containing protein 47 (CCDC47), and the nicalin-TMEM147-NOMO complex, localized at the ribosome exit tunnel, organized around a central membrane cavity revealed by cryo-electron microscopy.^9^ The translocon is a highly conserved multi-subunit protein complex that consists of three subunits (Sec61α, Sec61β, and Sec61γ) functioning as a protein-conducting channel connecting the cytoplasmic and luminal spaces on either side of the ER membrane. Within the translocon, pathogenic variants involved in a human disease have so far only been identified in *SEC61A1* (MIM: 609213), which encodes one of the Sec61α subunits of the translocon complex, generating an autosomal-dominant-inherited condition named tubulointerstitial kidney disease 5 (MIM: 617056), responsible for nephropathy with ID.^10^ Among the accessory factors, recessive pathogenic variants in *TMCO1* underlie cerebrofaciothoracic dysplasia (MIM: 213980)^11^, ^12^, ^13^ and recessive pathogenic variants in *CCDC47* are responsible for trichohepatoneurodevelopmental syndrome (THNS [MIM: 618268]).^14^ Both conditions are associated with moderate to severe intellectual disability and dysmorphic facial features.

Besides its localization at the ER membrane, TMEM147 also localizes at the nuclear envelope, where it interacts with the C-terminal domain of the lamin B receptor (LBR), anchoring it to the inner membrane.^15^ LBR is a lamin-binding protein from the inner nuclear membrane with sterol reductase activity that provides, together with lamins, essential heterochromatin docking sites at the nuclear envelope.^16^^,^^17^ In mouse models, the absence of both LBR and lamin A/C leads to loss of peripheral heterochromatin and an inverted architecture with heterochromatin localizing to the nuclear interior.^18^ In humans, heterozygous pathogenic variants in *LBR* (MIM: 600024) are associated with Pelger-Huët anomaly (MIM: 169400)^19^ and Reynolds syndrome (MIM: 613471),^20^ while bi-allelic variants are associated with Greenberg skeletal dysplasia (MIM: 215140)^21^ and rhizomelic skeletal dysplasia with or without Pelger-Huët anomaly (MIM: 618019).^22^^,^^23^ Pelger-Huët anomaly is characterized by abnormal nuclear shape and chromatin organization in blood granulocytes. Affected individuals show hypolobulated neutrophil nuclei with coarse chromatin. Homozygous individuals have ovoid neutrophil nuclei as well as varying degrees of developmental delay, epilepsy, and skeletal abnormalities.^21^ In mouse, deletion of the LBR N-terminal domain recapitulated Pelger-Huët anomaly without disrupting X chromosome inactivation.^24^

In HeLa cells, knockdown experiments of *TMEM147* with an RNAi approach suggested a significant reduction of Hoechst, H3K9me3, and LBR staining accompanied by a mislocalization of LBR to the ER, indicating potential defaults in chromatin condensation.^15^ By contrast, ER markers cytoskeleton-linking membrane protein CKAP4 (CLIMP-63) and RNT4 (NOGO) were upregulated.^15^^,^^25^ CKAP4 is a non-glycosylated type II ER membrane protein.^26^^,^^27^ Restricted localization of CKAP4 to the reticular part of the ER is mediated by self-association that retains the protein in the ER^28^ and limits its mobility in the membrane.^29^ The cytoplasmic segment of CKAP4 binds to microtubules, both *in vivo* and *in vitro*, and overexpression of the protein leads to a parallel rearrangement of ER and microtubules, suggesting that the protein links ER membranes to the microtubule cytoskeleton.^30^ RTN4 is a member of the reticulon family of proteins and is critical for regulating the tubular structure of the ER.^31^^,^^32^ Excess RTN4 limit ER luminal transport, Ca^2+^ release, and induced pluripotent stem cells (iPSCs)-derived cortical neuron’s axonal extension, while RTN4 elimination reverses the effect.^33^

**Figure 1:**
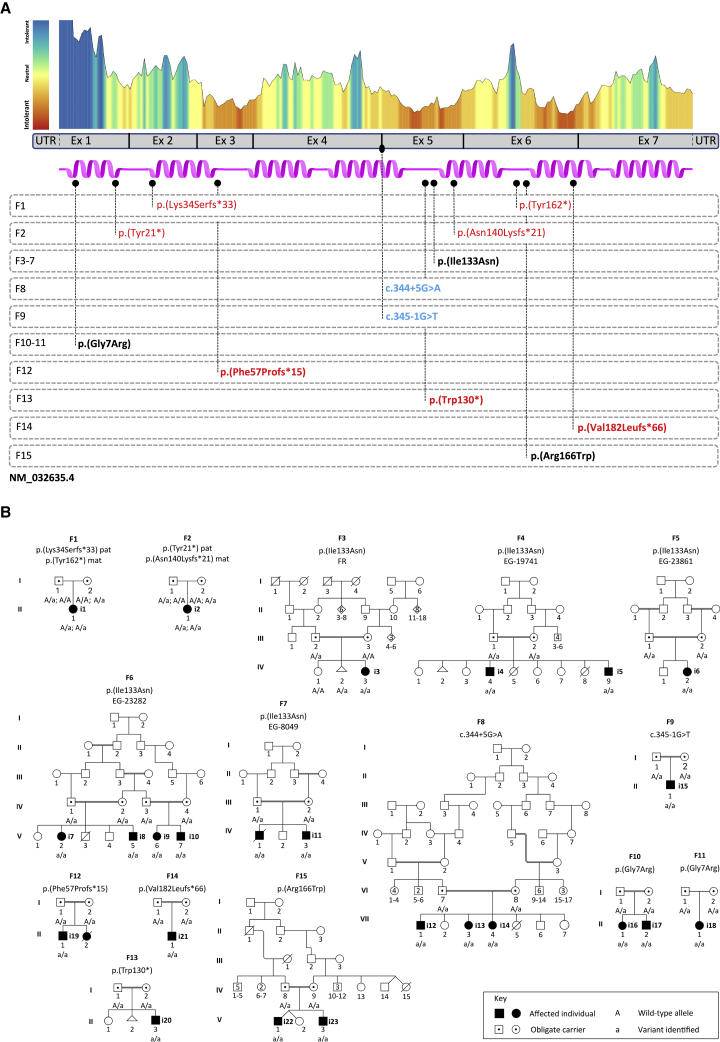
Individuals with *TMEM147* germline variants identified in the cohort (A) Representation of TMEM147 (purple) with its seven helices. Metadome constraint plot and distribution of exomic regions are reported above the TMEM147 model. Families with disease-causing variants are reported below. Homozygous variants are indicated in bold. Nonsense and frameshift variants are indicated in red, splice-site variant in blue, and missense variant in black. (B) Family pedigrees and segregation analysis of the identified variants. “A” represents the wild-type allele and “a” the mutated one.

**Figure 2:**
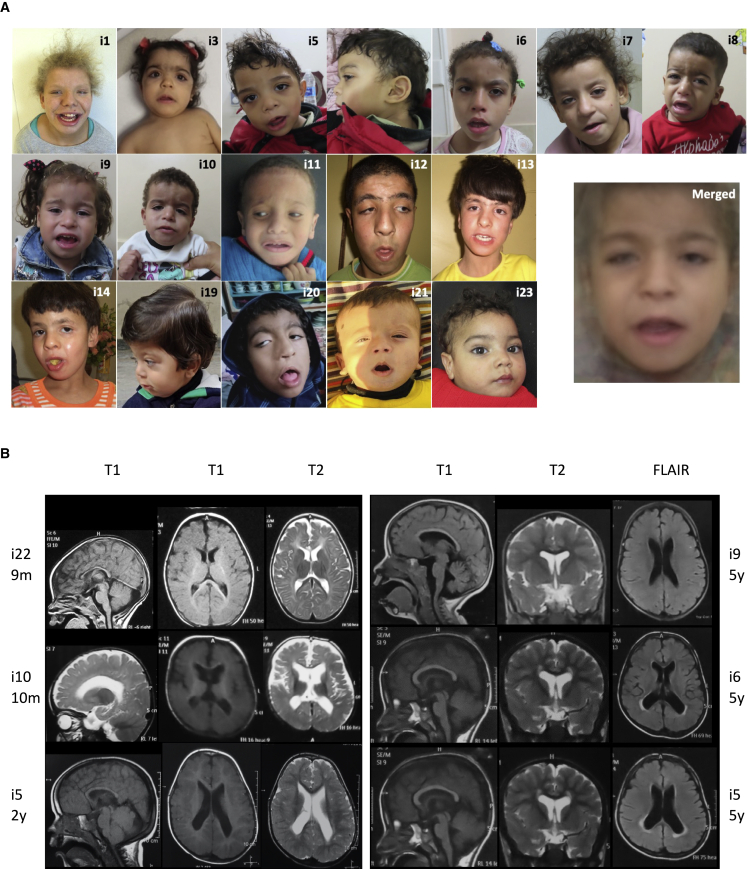
Photos of the affected individuals included in the cohort and brain MRI results (A) Affected individuals showed consistent coarse facial features. A merged image was obtained by the Facer tool. (B) Brain MRI of individuals with *TMEM147* disease-causing variants. Thin corpus callosum is present in all individuals whose brain MRIs were available. Subcortical atrophy with ventricle enlargement is observed in all individuals except i22 (F15-V-1) and i9 (F6-V-6) and was more pronounced in i10 (F6-V-7) at 10 months of age, associated with a cortical atrophy. According to age, myelination remains poor on MRI performed at 5 years of age (i5 – F4-IV-9, i6 – F5-IV-2, and i9 – F6-V-6), particularly in the temporal white matter with a hypersignal flair of the periventricular white matter in two individuals.

However, despite all previously developed elements, the involvement of *TMEM147* in human pathology remains uncertain to this day. Through an international data sharing mainly facilitated by GeneMatcher,^34^ we gathered 23 individuals from 15 unrelated families with bi-allelic pathogenic *TMEM147* variants (Table S1, Figure 1A). We obtained informed consent from all affected individuals or their legal representative for the sequencing procedures and the publication of their results along with clinical and molecular data. Special consent forms were signed authorizing publication of pictures when relevant. The study was performed within the framework of the GAD (“Génétique des Anomalies du Développement”) collection and approved by the appropriate institutional review board of Dijon University Hospital (DC2011-1332). These affected individuals displayed overlapping clinical features including moderate to profound ID, developmental delay, behavioral problems, and facial dysmorphism (Table 1, Figure 2A, extended clinical information is available in Table S2).

**Table 1:** Clinical features of the *TMEM147* cohort

Individual family	i1 F1	i2 F2	i3 F3	i4 F4	i5 F4	i6 F5	i7 F6	i8 F6	i9 F6	i10 F6	i11 F7	i12 F8	i13 F8	i14 F8	i15 F9	i16 F10	i17 F10	i18 F11	i19 F12	i20 F13	i21 F14	i22 F15	i23 F15	Total
Sex	F	F	F	M	M	F	F	M	F	M	M	M	F	F	M	F	M	F	M	M	M	M	M	10F/13M
Age at last follow-up	16 y	8 y	2 y	13 y	4 y 3 m	6 y 6 m	9 y 1 m	2 y 11 m	6 y	3 y 2 m	2 y 6 m	14 y	8 y	6 y	3 y	8 y	5 y	18 y	2 y	4 y 6 m	14 m	6 y	1 y	N/A
Motor delay	+	+	+	+	+	+	+	+	+	+	+	+	+	+	+	+	+	+	+	+	+	+	+	23/23
Walking age	2 y	5 y	na	4 y	na	3 y	4 y	na	5 y	na	na	3 y	3 y	3 y	3 y	UN	UN	3 y	1.5 y	na	na	na	na	N/A
Intellectual disability	+	+	UN	+	+	+	+	+	+	+	+	+	+	+	+	+	+	+	+	+	+	+	ty	21/21
Severity	UN	se	UN	IQ: 50	IQ: 45	IQ: 53	IQ: 48	IQ: 35–40	IQ: 45	IQ: 35	IQ: 20–25	se	se	se	UN	se	se	se	mo	se	UN	IQ: 35	ty	N/A
Speech ability upon last examination	sw	ns	ba	ss	ns	sw	sw	ns	sw	ns	ns	ns	sw	ns	sw	ss	sw	sw	sw	sw	ba	ns	UN	22/22
Behavioral problems	+	+	−	+	+	+	+	+	+	+	+	−	−	−	UN	+	+	+	−	+	−	+	−	15/22
Neurological abnormalities	+	−	+	−	−	−	−	−	−	+	+	−	−	−	−	−	+	−	−	−	−	+	−	6/23
Facial dysmorphism	+	+	+	+	+	+	+	+	+	+	+	+	+	+	+	+	UN	+	+	+	+	+	+	22/22
Brain MRI abnormalities	−	−	−	+	+	+	+	+	+	+	+	UN	UN	UN	−	UN	UN	+	+	+	−	+	+	13/18

M, male; F, female; y, years; m, months; na, not acquired; UN, unknown; ty, too young; se, severe; mo, moderate; sw, single words; ns, no speech; ba, babbling; ss, short sentences; N/A, not applicable.

Ten individuals were female (43%) and 13 male (57%). Family history was unremarkable for all families but one (F12) in which a younger sibling died of a possibly unrelated disorder, as familial segregation analysis showed that she was not homozygous for the same *TMEM147* variant as the proband (Figure 1B). Heterozygous carriers were healthy. Twenty individuals were born to consanguineous parents (87%). Most individuals were born following uneventful pregnancies and deliveries (18/23, 78%); one pregnancy was marked by severe intrauterine growth retardation at 27 gestational weeks, facial dysmorphism evocative of Cornelia de Lange syndrome and preeclampsia (i3 – F3-IV-3), two were marked by reduced placenta flow (i6 – F5-IV-2 and 8 – F6-V-5), another one was marked by a neonatal hypoxic ischemic event (i20 – F13-II-3), and one individual delivered by C-section for fetal distress and meconial amniotic fluid (i1 – F1-II-1). She was admitted in neonatology during her first days of life with feeding and breathing difficulties. Delivery was at full term for 17 individuals (74%), at 37 weeks for four individuals (i15 – F9-II-1, i19 – F12-II-1, i20 – F13-II-3, i21 – F14-II-1), and at 35 weeks for two individuals (i3 – F3-IV-3, i5 – F4-IV-9), all from different families. Birth measurements were within normative values for most individuals; however, five presented a birth weight below −3 standard deviations, including four from the same family (i3 - F3-IV-3, i7 - F6-V-2, i8 - F6-V-5, i9 - F6-V-6, i10 - F6-V-7) and i3 (F3-IV-3), who also presented birth length and orbitofrontal circumference (OFC) below −3 standard deviations (Table S2). Data on growth parameters upon last follow-up were collected at heterogenous ages and mostly within normative values. However, eight individuals (35%) presented at least one measurement below −2 standard deviations. Amongst them, four presented isolated microcephaly that was not present at birth. Detailed measurements are presented in Table S2.

All individuals had global developmental delay and intellectual disability. Language delay was severe: upon their last follow-up, eight individuals (36%) had developed no speech at all, 12 (55%) could only babble or speak a few words, and only two (9%) could make short sentences. Of note, one individual was too young at his last examination for a reliable language evaluation (i23 – F15-V-3, age 1 year). Gross and fine motor skills were impaired for all individuals, although less severe than language delay, as most children were ambulatory by 5 years of age. Behavioral problems were observed in 15 individuals (65%) who shared a common tendency of self-injury. Other recurrent behavioral problems included hyperactivity, aggressivity, and outbursts of anger. All individuals presented ID, which was considered severe for all but one individual (either through neuropsychological testing or simple clinical evaluation).

All individuals (22/22, one missing information) displayed consistent facial dysmorphisms including coarse facies, prominent forehead, board depressed nasal root, tented mouth, long smooth philtrum, and low-set ears as major features (Table 1, Table S2, Figure 2A). We performed a computer-assisted facial visualization^35^ from all available photographs in order to generate a “typical” face for a person with this *TMEM147*-associated disorder by using the Facer program (https://github.com/johnwmillr/Facer). These analyses confirmed that morphological features were marked in *TMEM147*-related disorder and highlighted some of the morphological features identified in the individuals: high forehead, long philtrum, tented mouth (Figure 2). It is noteworthy these facial particularities led attending physicians to clinically and independently suspect either an RAS-associated disorder or a chromatinopathy. Among the 11 individuals for whom a cardiac ultrasound was available, two presented a patent foramen ovale, one presented an atrial septum defect, and one a large patent ductus arteriosus. Neurological examinations were mostly unremarkable or limited to signs related to motor-development delay. Of note, three individuals (14%) presented with hypotonia, two had tonic seizures (9%), and one had synkinesis with mirror movements of the hands that spontaneously resolved during childhood. Brain MRIs were available for 18 individuals and were mostly normal but revealed features including enlarged ventricles, thin corpus callosum, and white matter hyperintensities that were evocative of myelination delay alongside with mild cerebellar atrophy without dysplasia in some individuals. Cortical and subcortical atrophy was marked in only one individual (i10 – F6-V-7). Myelination delay was mostly observed in posterior regions (temporal lobes) with visible improvement upon follow-up (i5 – F4-IV-9, available MRIs at 2 and 5 years of age). Corpus callosum was thin in most individuals but never dysplastic (Figure 2B).

Twelve different variants were identified and included stop-gain (3/12), frameshift (4/12), missense (3/12), and splice-site variants (2/12) (Table S1, Figure 1). Interestingly, one missense variant (c.398T>A [p. Ile133Asn] [GenBank: NM_032635.4]) was found in five different families (F3 living in France but from North African ancestry, and F4 to F7 coming from Egypt), while a different missense variant (c.19G>C [p.Gly7Arg]) was found in two different families (F10 living in the United States and F11 coming from French and Spanish ancestries). All splice-site, nonsense, or frameshift variants but the c.540_543dup (p.Val182Leufs^∗^66), which is 12 nucleotides before the last exon-intron junction, were predicted to result in nonsense-mediated mRNA decay (NMD).^36^^,^^37^ However, in the p.Val182Leufs^∗^66 variant, the last 45 amino acids, encoding the C-terminal domain of the protein encompassing almost one third of the 6^th^ and the entire 7^th^ helix, were replaced by a sequence of 66 amino acids with no significant similarity to any known human protein. Regarding the splice-site variants c.344+5G>A and c.345−1G>T, *in silico* analysis predicted them to suppress the activity of the donor splice-site of exon 4 (137 nt) and the acceptor splice-site of exon 5 (85 nt), respectively (Figures S2A–S2D). Both were predicted to alter the reading frame of the transcript (Figures S2A and S2C),^38^ eventually resulting in nonsense-mediated mRNA decay. A minigene approach was established in order to measure the functional readout of the c.344+5G>A variant, confirming exon 4 skipping (Figure S2E). Most variants were present in gnomAD, but none were present at a homozygous state (Table S1). Given the prevalence of bi-allelic variants predicted to result in loss of function (LoF, i.e., nonsense, frameshift, and splice-site variants) and a congruent phenotype spectrum in the cohort, the identified missense variants were predicted to alter protein stability and function. Indeed, the three missense variants affected conserved amino acids but were predicted to variably impact protein function according to CADD,^39^ PolyPhen2,^40^ GERP,^41^ REVEL,^42^ 3Cnet,^43^ and Metadome^44^ algorithms (Figure 1, Table S1).

**Figure 3:**
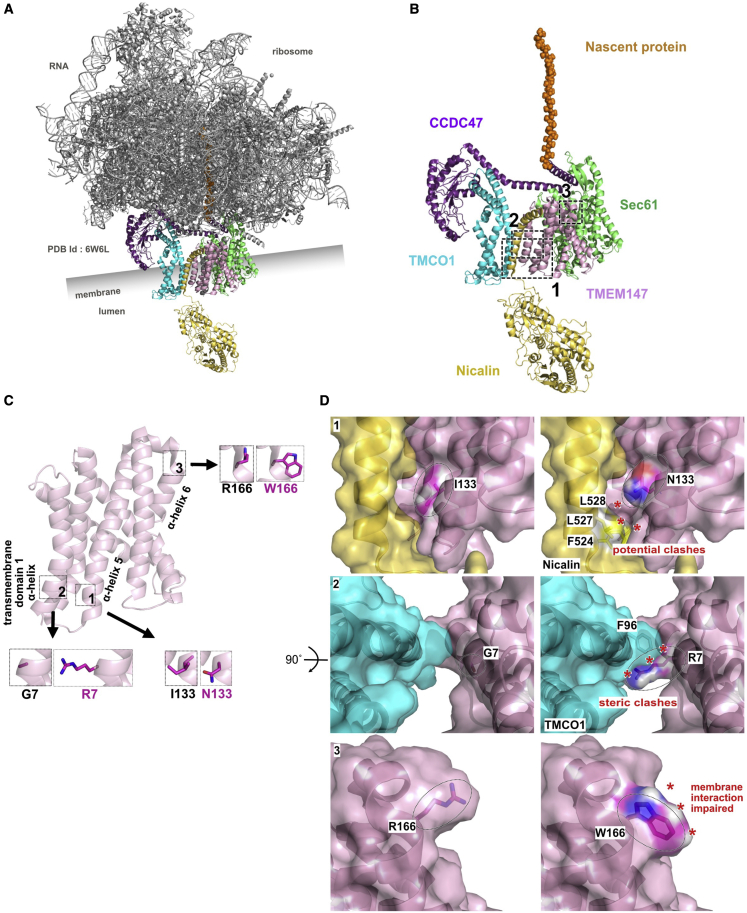
Structural analysis of the p.Gly7Arg, p.Ile133Asn, and p.Arg166Trp variants (A) Cartoon representation of the ER translocon structure (PDB: 6W6L).^9^ The five proteins involved in the transmembrane channel are colored: Nicalin (yellow), Sec61 (green), TMCO1 (cyan), CCDC47 (purple), and TMEM147 (pink). Ribosomal proteins and RNA are presented in gray. Membrane position is indicated with the lumen. Nascent protein sequence is shown as orange spheres inside the ribosome structure. (B) Structural representation of TMEM147 and its main partners. For better visualization, ribosomal proteins and RNAs have been masked. Three dotted squares indicate the magnified regions used to model the variant effects. Squares 1, 2, and 3 indicate the enlarged regions used to model the p.Ile133Asn, p.Gly7Arg, and p.Arg166Trp variants, respectively. (C) Cartoon representation of TMEM147 presenting the three enlarged regions. In the larger dotted inserts, the wild-type situation for each amino acid is displayed as stick. 1, Ile133; 2, Gly7; and 3, Arg166. In the adjacent insert, the mutated amino acid is modeled as a comparison. Only the side chains are represented in purple, leaving the α-carbon as a pink ribbon representation. Hydrogens have been masked for better visualization. (D) (1, left) Surface rendering of the Ile133 area. The α-helix faces the carboxy-terminal region of Nicalin. (1, right) Surface rendering of the Ile133Asn variant as a model. From the 11 possible rotamers for asparagine in this context, the one causing the minimum of constraints was selected. (2, left) Surface rendering of the Gly7 area. The molecule was rotated by 90° and viewed from above. (2, right) Surface rendering of the Gly7Arg variant as a model. From the 24 possible rotamers for arginine in this context, all of them are causing mild to severe steric clashes with TMEM147 itself or the surrounding polypeptidic chains. The rotamer causing the lowest degree of perturbation was arbitrarily selected. (3, left) Surface rendering of the Arg166 area. (2, right) Surface rendering of the p.Arg166Trp variant as a model. From the seven possible rotamers for tryptophan in this context, all of them are causing mild steric clashes with TMEM147. The rotamer causing the lowest degree of perturbation was arbitrarily selected. Dotted shapes indicate the positions of the amino acids in the chain.

To provide functional insight into the impact of the TMEM147 variants, we performed an *in silico* structural analysis. Gly7 is located in the transmembrane domain 1, at the beginning of the α-helix 1. Ile133 is located at the beginning of α-helix 5 between the transmembrane domains 3 and 4, while Arg166 is located at the beginning of α-helix 6 where the transmembrane region initiates (Figure S1). The 3D structure of the translocon was recently resolved by cryo-electron microscopy (PDB: 6W6L, Figure 3A)^9^ and used here to further explore the possible functional impact of the three missense changes (Figures 3B and 3C). Considering the p.Gly7Arg substitution, the arginine extension was predicted to push into the TMCO1-facing amino terminal region, creating a steric conflict with the Phe96 (Figure 3, insert 2, asterisks). Similarly, p.Ile133Asn generated tensions with the adjacent amino acids in TMEM147 (data not shown) and its extension towards nicalin, would cause potential clashes with three amino acids in nicalin: Phe524, Leu527, and Leu528 (Figure 3, insert 1, asterisks). The c.496C>T (p.Arg166Trp) variant changed the surface environment and net charge. Even though this side of the chain is not in direct contact with other proteins of the ER translocon, it is located in the vicinity of the membrane itself and the transmembrane part of the α-helix 6. Such drastic changes in both conformation and charges could explain the instability observed for this variant (Figure 3, insert 3).

**Figure 4:**
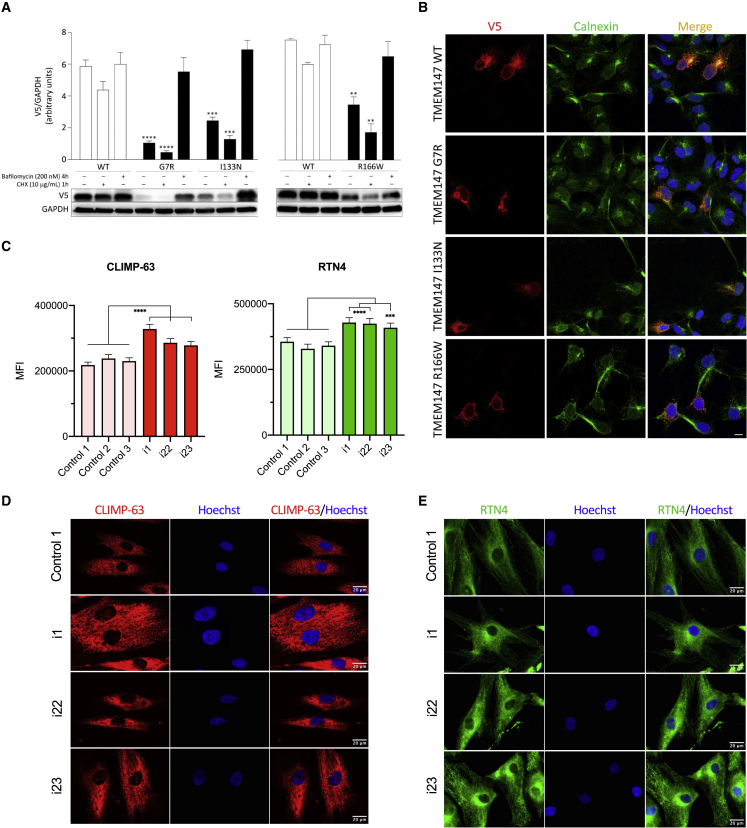
Biochemical characterization of the TMEM147 mutant proteins and immunostainings in fibroblasts (A) Accelerated degradation of the TMEM147^Arg7Gly^ (R7G), TMEM147^Ile133Asn^ (I133N), and TMEM147^Arg166Trp^ (R166W) proteins. Immunoblot analysis shows WT and variant V5-tagged TMEM147 protein levels in transfected COS-1 cells, basally and after CHX (10 μg/mL) or bafilomycin (200 nM) treatment. GAPDH was used as loading control. Representative blots (below) and mean ± SD densitometry values (above) of three independent experiments are shown. Asterisks indicate statistically significant differences compared with WT TMEM147 (^∗∗∗∗^p ≤ 0.0001; ^∗∗∗^p ≤ 0.001; ^∗∗^p ≤ 0.05; two-way ANOVA followed by Tukey’s multiple comparison test). (B) Subcellular localization of transiently expressed V5-tagged WT or mutant TMEM147 proteins in COS-1 cells under steady-state conditions revealed by confocal microscopy analysis. Cells were stained with the anti-V5 monoclonal antibody (red). Co-localization analysis was performed using the endoplasmic reticulum marker calnexin (green). Merged images with nuclei (Hoechst 33342 staining, blue) are displayed on the right. Scale bar, 10 μm. (C) Quantification of mean fluorescence signals ± SEM detected in (D). Three technical replicates were performed per cell line. A total of 150 measurements per cell line were performed. Asterisks indicate statistically significant differences compared with cell lines from healthy individuals: control 1, control 2, and control 3 (^∗∗∗∗^p ≤ 0.0001; ^∗∗∗^p ≤ 0.001; ^∗∗^p ≤ 0.01; ^∗^p ≤ 0.05; one-sample Wilcoxon test, based on the average of the three control samples). (D and E) Localization analysis of CKAP4 (CLIMP-63) (D) and RTN4 (E) in fibroblasts from i1, i22, and i23 and healthy control individuals (only control 1 is shown in the figure). Scale bar, 20 μm.

To validate the impact of the identified missense variants on TMEM147 stability, we generated three V5-tagged TMEM147 mutants and assessed the protein level of each in transiently transfected COS-1 cells, basally and after 1 h of treatment with the protein synthesis inhibitor cycloheximide (CHX). Immunoblotting and relative quantitative analyses revealed a variably reduced level of the three TMEM147 mutants, indicating that the tested variants significantly impact TMEM147 stability; p.Gly7Arg was the most unstable and p.Arg166Trp was the least (Figure 4A). Treatment with bafilomycin, a late-stage inhibitor of autophagy and lysosomal protein degradation, rescued the reduced levels of all mutants (Figure 4A), indicating that accelerated degradation of TMEM147 mutant proteins occurs via the autophagy-lysosomal pathway. We then imaged COS-1 cells transiently transfected with plasmids encoding V5-tagged TMEM147, and we confirmed the localization of the wild-type (WT) protein to the endoplasmic reticulum, where it colocalizes with calnexin (Figure 4B). Assessment of the subcellular localization of the TMEM147 mutants showed that, similarly to the WT protein, all tested proteins colocalized with calnexin, indicating proper targeting to the endoplasmic reticulum (Figure 4B).

**Figure 5:**
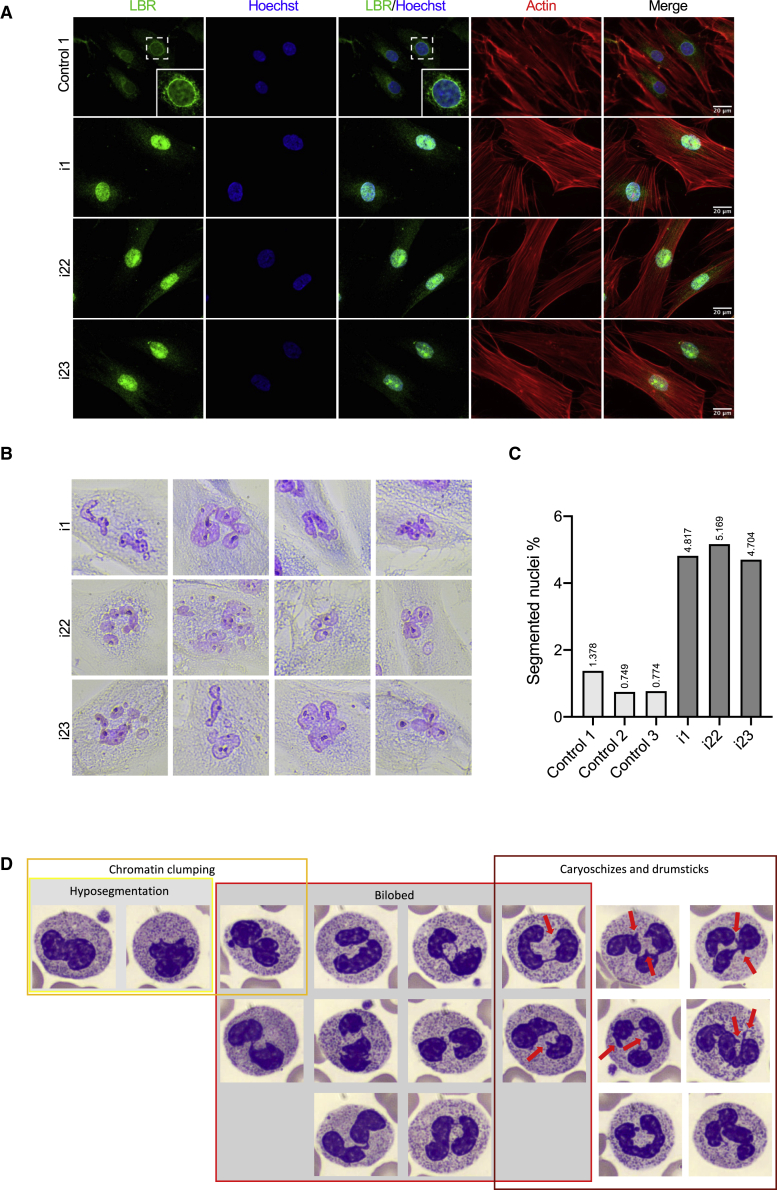
LBR localization, nuclear morphology in fibroblasts, and Pelger-Huët-like anomaly in granulocytes (A) Localization analysis of LBR and actin in fibroblasts from i1, i22, and i23 and healthy control individuals (only control 1 is shown in the figure). Scale bar, 20 μm. The inset in control 1 line shows a higher magnification of the nucleus indicated by the dashed square. Actin staining shows that TMEM147 does not affect gross cell morphology. (B) Nuclear segmentation is observed in fibroblast cell nuclei from affected individuals. Fibroblasts were stained with the May Grunwald-Giemsa (MGG) method. (C) Quantification of the nuclear segmentation observed in fibroblast cell lines. (D) Chromatin clumping, hyposegmentation, bilobed nuclei, carioschizes, and drumsticks (red arrows) observed in neutrophils of i1.

Next, we sought to investigate the functional readout of TMEM147 bi-allelic loss of function in derived primary cultures. For this purpose, we obtained primary fibroblast cells from i1 (F1-II-1) and i22 and i23 (F15-V-1 and F15-V-3, respectively) with compound heterozygous nonsense c.486C>G (p.Tyr162^∗^) and frameshift c.100_118del (p.Lys34Serfs^∗^33) variants or the homozygous p.Arg166Trp missense variant in *TMEM147*, respectively. ER markers CKAP4 (CLIMP-63) and RTN4 were significantly upregulated in TMEM147 individuals as compared to healthy volunteers (Figures 4C–4E). This upregulation was accompanied by a concomitant parallel rearrangement of ER, indicating that in absence of TMEM147, morphological and physiological changes take place (Figure 4D and 4E). *TMEM147*-deficient cells were previously shown to alter LBR localization. We thus performed LBR localization analysis in fibroblasts from healthy controls and affected individuals and observed that it was restricted to the nuclear envelope in the former, while it was evenly distributed throughout the nucleus in the latter condition (Figure 5A). Interestingly, nuclei from affected individuals were associated with a higher risk of showing abnormal segmentation as compared to cultures from healthy individuals, indicating that LBR mislocalization could cause nuclear instability (Figures 5D and 5C).

Finally, we hypothesized that hematological abnormalities such as Pelger-Huët anomaly would likely be present in *TMEM147*-null individuals. Cytological examination of the blood smear frequently revealed lobulation defects of the neutrophils (Figures 5D and S3A), with forms of hyposegmentation similar to the Pelger-Huët anomaly, although with a lower proportion, bilobed aspects in the shape of a sack with two round or oval lobes connected by a thread of chromatin, rare chromatin clumping, and frequent nuclear appendices (caryoschizes or drumsticks), isolated or associated with the preceding abnormalities. These cytological abnormalities concerned about 15%–25% of neutrophils and were also identified in sparse eosinophils, indicating that the anomaly affects all granulocytes but is most evident in polymorphonuclear neutrophils (Figure S3B). None were observed in platelets, red blood cells, lymphocytes, and monocytes. Formally, these hematological features could not be classified as a typical Pelger-Huët anomaly, but rather pseudo-Pelger-Huët anomaly because the majority of mature granulocytes were not bilobed, although they clearly belonged to the same phenotypic spectrum.

*TMEM147* is widely expressed in the developing human brain (GTEx: https://gtexportal.org/home/). To further explore the relevance of *TMEM147* function during neurodevelopment, we studied its correlation with an established set of neurodevelopmental disorder (NDD)-associated genes.^45^ By using the ARCHS4 database (ARCHS4: https://maayanlab.cloud/archs4/),^46^ we observed that *TMEM147* expression is significantly correlated to this group of genes in the brain (Figure S4A, p value < 0.01) and that this correlation of expression patterns is brain specific (Figure S4B). To further support this tissue-specific correlation, NDD genes with correlation values greater than |0.3| with *TMEM147* were also analyzed by means of GTEx database. Consistently, the cluster analysis highlighted that the co-expression profile of *TMEM147* and its most correlated NDD-associated genes clustered in two main groups, one of which specifically containing all brain sites, indicating a tissue-specific correlation among these genes (Figure S4C). Although several individuals in our cohort presented white matter abnormalities such as white matter atrophy, thin corpus callosum, dilated ventricles, enlarged cortical sulci, and possible myelination delay, it is noteworthy that *TMEM147*-related disorders cannot be considered as primary white matter disorders but rather as encephalopathies. Indeed, the lack of upper-motor neuron syndrome in all individuals, the presence of global hypotonia in several individuals, and the overall lack of clear white matter hypomyelination or demyelination in the available brain MRI is congruent with an encephalopathy rather than a leukodystrophy. Interestingly, among the list of NDD genes with strong co-expression with *TMEM147*, nine genes correlated with the HPO term “encephalopathy” (HP: 0001298), i.e., *CDKL5* (developmental and epileptic encephalopathy [MIM: 300672]), *CLTC* (intellectual developmental disorder, autosomal dominant 56 [MIM: 617854]), *KMT2E* (O’Donnel-Luria-Rodan syndrome [MIM: 618512]), *NRXN1* (Pitt-Hopkins-like syndrome 2 [MIM: 614325]), *SCN1A* (developmental and epileptic encephalopathy 6B, non-Dravet [MIM: 619317]; Dravet syndrome [MIM: 607208]; febrile seizures, familial, 3A [MIM: 604403]), *SCN2A* (developmental and epileptic encephalopathy 11 [MIM: 613721]), *TCF4* (Pitt-Hopkins syndrome [MIM: 610954]), *UNC80* (hypotonia, infantile, with psychomotor retardation and characteristic facies 2 [MIM: 616801]), and *WDR45* (neurodegeneration with brain iron accumulation 5 [MIM: 300894]).

The individuals in our cohort presented global developmental delay with particularly severe speech delay (only one individual could make short sentences upon last follow-up) and psychomotor delay. Even though all individuals had delayed motor milestones, they all eventually acquired walking, although gait was often described as imperfect or unsteady. Clinical follow-up revealed that fine motor skills were also impaired. Severe intellectual disability was observed in all individuals. Beyond these developmental features, the individuals’ phenotype was mostly made of marked facial dysmorphism. Of note, the facial features of a large proportion of these individuals were suggestive of a RASopathy or a chromatinopathy.^47^, ^48^, ^49^, ^50^ For some, sequencing of a panel of genes associated with these diseases was performed. However, Episign analysis revealed a genome-wide DNA methylation profile incompatible with all 59 episignatures assessed, including chomatinopathy conditions such as Cornelia de Lange, Rubinstein-Taybi, Coffin-Siris, and Wiedemann-Steiner syndromes. A typical positive case presents with a methylation variant pathogenicity (MVP) greater than 0.5 and both tested individuals presented with MVPs of 0.02 or less (Figure S4D).

TMEM147 was originally identified as a protein complexing with NOMO and nicalin.^5^ On the basis of the documented codependence among the three proteins, dysregulation of *TMEM147* is expected to alter the nicalin-NOMO protein complex and, consequently, the various molecular pathways it is involved in. Nicalin-NOMO interaction has been shown to modulate (antagonize) the Nodal pathway.^7^ For instance, previous efforts had shown that the Nodal pathway is paramount in early embryonic development where it takes part in mesoendoderm induction,^51^ right-left patterning,^52^^,^^53^ and neural patterning as Nodal antagonism is required for the formation of neuroectoderm.^51^ Some actors of the TGF-β superfamily even take part in the final patterning of the vertebrate central nervous system.^54^ Overall, these elements made *TMEM147* an even stronger candidate for neurodevelopmental disorders.

Here, we described a cohort of 23 affected individuals identified through an international collaboration who harbored bi-allelic disease-causing variants in *TMEM147*, thus offering a clinical profiling of a neurodevelopmental disorder caused by *TMEM147* loss of function. Splice-site, nonsense, or frameshift disease-causing variants were largely represented in our cohort. Missense variants, resulting in accelerated degradation of the mutated protein via the autophagy-lysosomal pathway, were identified in several unrelated families showing consistent clinical features. No phenotypic differences between affected individuals with predicted loss-of-function versus missense variants were noted. The analysis of primary fibroblasts and granulocytes provided functional evidence of endoplasmic reticulum and the nuclear envelope dysfunction. Interestingly, previous experiments had shown that *TMEM147* knockdown results in a drastic decline of LBR protein levels, an altered diffusional mobility of LBR, and its relocation to the ER in HeLa cells.^15^ However, our experiments demonstrate that the diffusional mobility of LBR is not accompanied by decreased protein levels in primary fibroblast cells from affected individuals. *TMEM147* is ubiquitously expressed, however co-expression data suggested a strong correlation with neurodevelopmental genes linking subcellular dysfunction to key genes involved in brain development.

Overall, *TMEM147* should be considered as a gene responsible for intellectual disability, developmental delay, and facial dysmorphism and should be considered as a potential differential diagnosis with chromatinopathies or RASopathies.

## Data Availability

All of the data produced in this study are included in the main manuscript and in supplemental material.

## Supplemental information

Document S1. Figures S1–S4, Tables S1 and S2, and supplemental material and methods

Document S2. Article plus supplemental information
