# Supplementary material for: Bi-allelic loss-of-function variants in TMEM147 cause moderate to profound intellectual disability with facial dysmorphism and pseudo-Pelger-Huët anomaly
Source: Am J Hum Genet. 2022 Aug 30;109(10):1909–22. doi: 10.1016/j.ajhg.2022.08.008 (PMC9606387; doi:10.1016/j.ajhg.2022.08.008)
Supplement: Document S1. Figures S1–S4, Tables S1 and S2, and supplemental material and methods [file mmc1.pdf]

The American Journal of Human Genetics, Volume *109*

Supplemental information

Bi-allelic loss-of-function variants in *TMEM147* cause moderate to profound intellectual disability with facial dysmorphism and pseudo-Pelger-Huët anomaly

Quentin Thomas, Marialetizia Motta, Thierry Gautier, Maha S. Zaki, Andrea Ciolfi, Julien Paccaud, François Girodon, Odile Boespflug-Tanguy, Thomas Besnard, Jennifer Kerkhof, Haley McConkey, Aymeric Masson, Anne-Sophie Denommé-Pichon, Benjamin Cogné, Eva Trochu, Virginie Vignard, Fatima El It, Lance H. Rodan, Mohammad Ayman Alkhateeb, Rami Abou Jamra, Laurence Duplomb, Emilie Tisserant, Yannis Duffourd, Ange-Line Bruel, Adam Jackson, Siddharth Banka, Meriel McEntagart, Anand Saggar, Joseph G. Gleeson, David Sievert, Hyunwoo Bae, Beom Hee Lee, Kisang Kwon, Go Hun Seo, Hane Lee, Anjum Saeed, Nadeem Anjum, Huma Cheema, Salem Alawbathani, Imran Khan, Jorge Pinto-Basto, Joyce Teoh, Jasmine Wong, Umar Bin Mohamad Sahari, Henry Houlden, Kristina Zhelcheska, Melanie Pannetier, Mona A. Awad, Marion Lesieur-Sebellin, Giulia Barcia, Jeanne Amiel, Julian Delanne, Christophe Philippe, Laurence Faivre, Sylvie Odent, Aida Bertoli-Avella, Christel Thauvin, Bekim Sadikovic, Bruno Reversade, Reza Maroofian, Jérôme Govin, Marco Tartaglia, and Antonio Vitobello

A

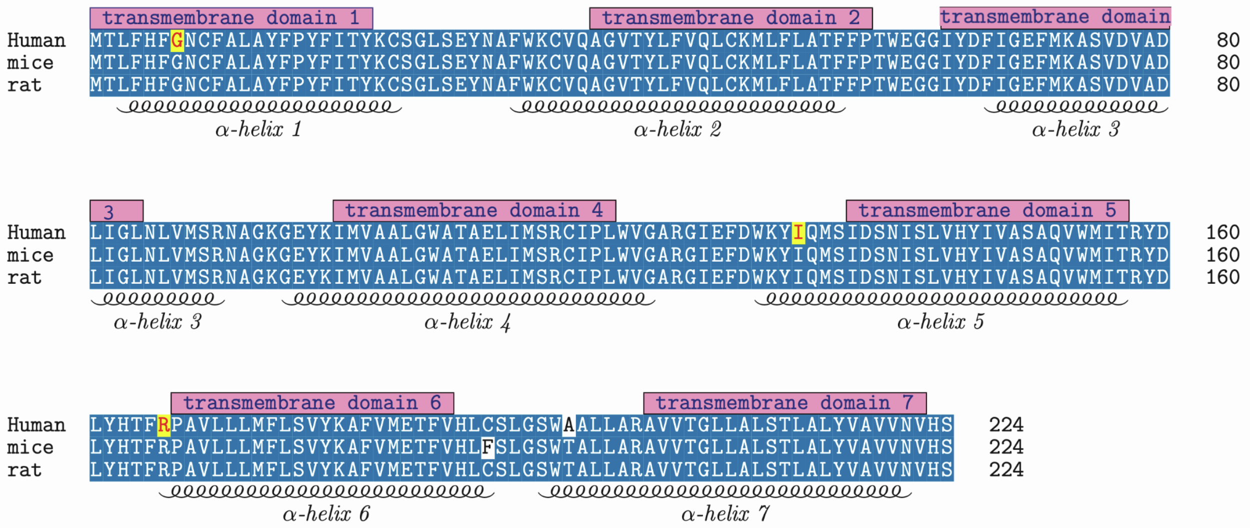
transmembrane domain 1
transmembrane domain 2
transmembrane domain
Human MTLFHFGNCFALAYFPYFITYKCSGLSEYNAFWKCVQAGVTYLFVQLCKMLFLATFFPTWEGGIYDFIGEFMKASVDVAD 80
mice MTLFHFGNCFALAYFPYFITYKCSGLSEYNAFWKCVQAGVTYLFVQLCKMLFLATFFPTWEGGIYDFIGEFMKASVDVAD 80
rat MTLFHFGNCFALAYFPYFITYKCSGLSEYNAFWKCVQAGVTYLFVQLCKMLFLATFFPTWEGGIYDFIGEFMKASVDVAD 80
α-helix 1
α-helix 2
α-helix 3
3
transmembrane domain 4
transmembrane domain 5
Human LIGLNLVMSRNAGKGEYKIMVAALGWATAELIMSRCIPLWVGARGIEFDWKYIQMSIDSNISLVHYIVASAQVWMITRYD 160
mice LIGLNLVMSRNAGKGEYKIMVAALGWATAELIMSRCIPLWVGARGIEFDWKYIQMSIDSNISLVHYIVASAQVWMITRYD 160
rat LIGLNLVMSRNAGKGEYKIMVAALGWATAELIMSRCIPLWVGARGIEFDWKYIQMSIDSNISLVHYIVASAQVWMITRYD 160
α-helix 3
α-helix 4
α-helix 5
transmembrane domain 6
transmembrane domain 7
Human LYHTFRPAVLLLMFLSVYKAFVMETFVHLCSLGSWAALLARAVVTGLLALSTLALYVAVVNVHS 224
mice LYHTFRPAVLLLMFLSVYKAFVMETFVHLFSLGSWTALLARAVVTGLLALSTLALYVAVVNVHS 224
rat LYHTFRPAVLLLMFLSVYKAFVMETFVHLCSLGSWTALLARAVVTGLLALSTLALYVAVVNVHS 224
α-helix 6
α-helix 7

B

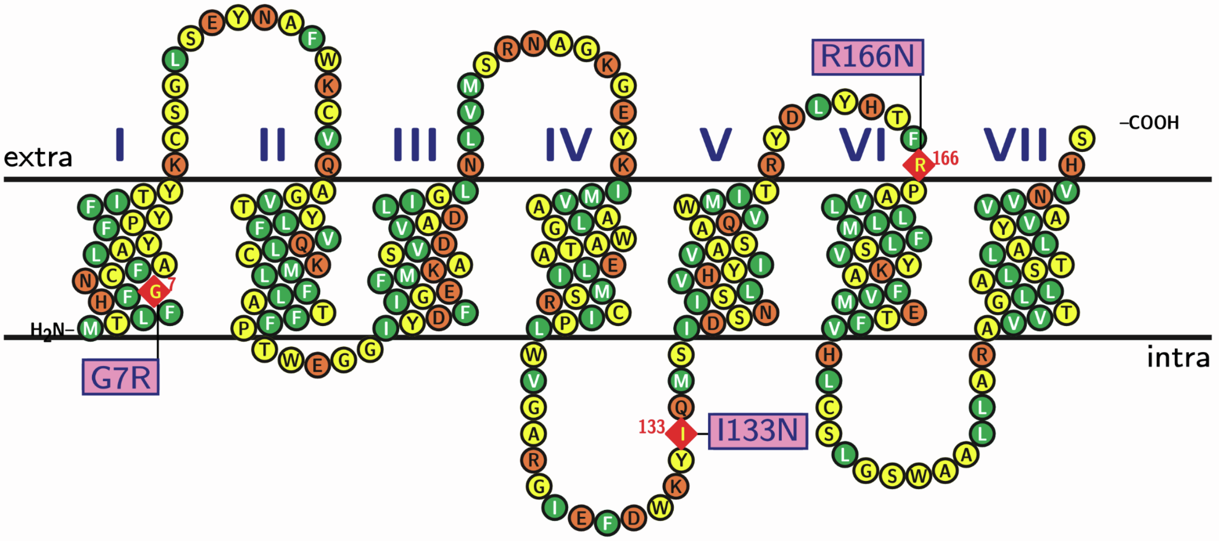
extra
intra
I
II
III
IV
V
VI
VII
H_2N–
–COOH
R166N
166
G7R
133
I133N

Figure S1 TMEM147 protein organization. (A) Clustal Ω alignment between the human, rat and mice sequences of TMEM147. The three sequences are 224 amino acid long and share above 99% identity. The seven transmembrane domains are indicated above the alignment and the α-helix regions are indicated below (from UniProt: Q9BVK8 and AlphaFold: AF-Q9VBK8-F1). Mutation positions are shown in red on a yellow background in the human sequence. The alignment was imaged with the TEXshade package in LaTeX.

(B) Membrane protein topology plot. The seven transmembrane domains are represented spaced by short loops. Mutation positions are indicated in pink boxes and the wild-type amino acid as a red diamond in the sequence string. Amino acids colored orange are considered external, green ones internal and yellow ambivalent in the structure. The plot was generated based on data found both in the UniProt and the AlphaFold databases using the TEXtopo package in LaTeX.

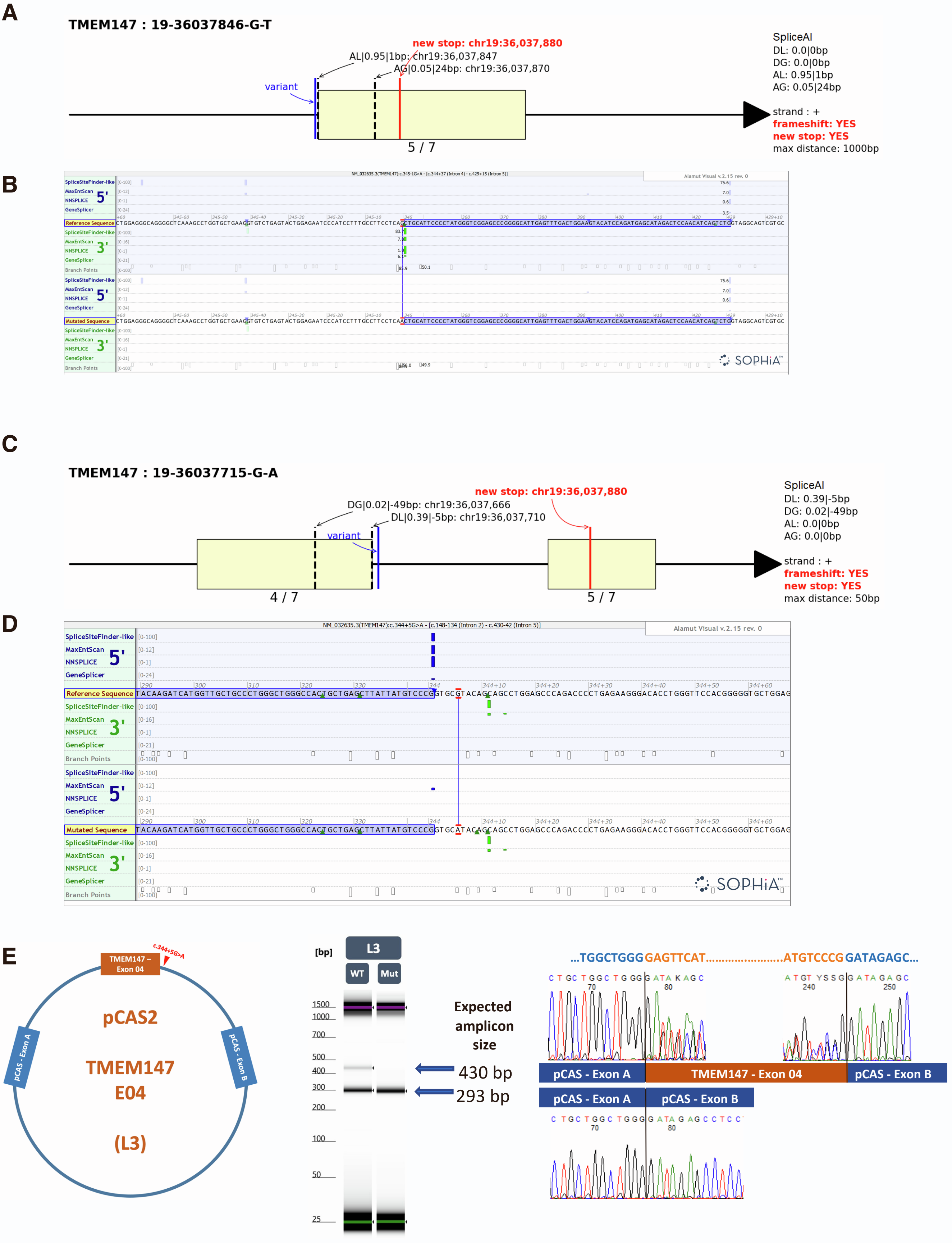
A
TMEM147 : 19-36037846-G-T
new stop: chr19:36,037,880
AL|0.95|1bp: chr19:36,037,847
AG|0.05|24bp: chr19:36,037,870
variant
5 / 7
SpliceAI
DL: 0.0|0bp
DG: 0.0|0bp
AL: 0.95|1bp
AG: 0.05|24bp
strand : +
frameshift: YES
new stop: YES
max distance: 1000bp
B
Reference Sequence
Mutated Sequence
SpliceSiteFinder-like
MaxEntScan
NNSPLICE
GeneSplicer
Branch Points
5'
3'
SOPHiA
C
TMEM147 : 19-36037715-G-A
new stop: chr19:36,037,880
DG|0.02|-49bp: chr19:36,037,666
DL|0.39|-5bp: chr19:36,037,710
variant
4 / 7
5 / 7
SpliceAI
DL: 0.39|-5bp
DG: 0.02|-49bp
AL: 0.0|0bp
AG: 0.0|0bp
strand : +
frameshift: YES
new stop: YES
max distance: 50bp
D
NM_032635.3(TMEM147):c.344+5G>A - [c.148-134 (Intron 2) - c.430-42 (Intron 5)]
Alamut Visual v.2.15 rev. 0
Reference Sequence
Mutated Sequence
SpliceSiteFinder-like
MaxEntScan
NNSPLICE
GeneSplicer
Branch Points
5'
3'
SOPHiA
E
TMEM147 – Exon 04
c.344+5G>A
pCAS2
TMEM147
E04
(L3)
pCAS - Exon A
pCAS - Exon B
[bp]
L3
WT
Mut
1500
1000
700
500
400
300
200
100
50
25
Expected amplicon size
430 bp
293 bp
...TGGCTGGG GAGTTCAT.......................ATGTCCCG GATAGAGC...
pCAS - Exon A
TMEM147 - Exon 04
pCAS - Exon B
pCAS - Exon A
pCAS - Exon B

Figure S2 Splice-site variants predictions and minigene results. SpliceAI score-based drawing (SpliceVI - 3 billion) and Alamut (Sophia Genetics) prediction of c.345-1G>T (A-B) and c.344+5G>A (C-D) splice-site variants. (E) Minigene analysis of the c.344+5G>A variant showing exon 4 skipping. The construct backbone (L3) utilized for this analysis is shown on the left. The red arrowhead indicates the position of the intronic variant. Reverse transcription polymerase chain reaction (RT-PCR) products obtained from wild-type (WT) and mutated (Mut) minigene constructs. The Sanger sequencing results of the amplicons obtained from the minigene analysis are shown on the right, confirming the inclusion of exon 4 in the WT construct and exon skipping in the Mut construct.

A

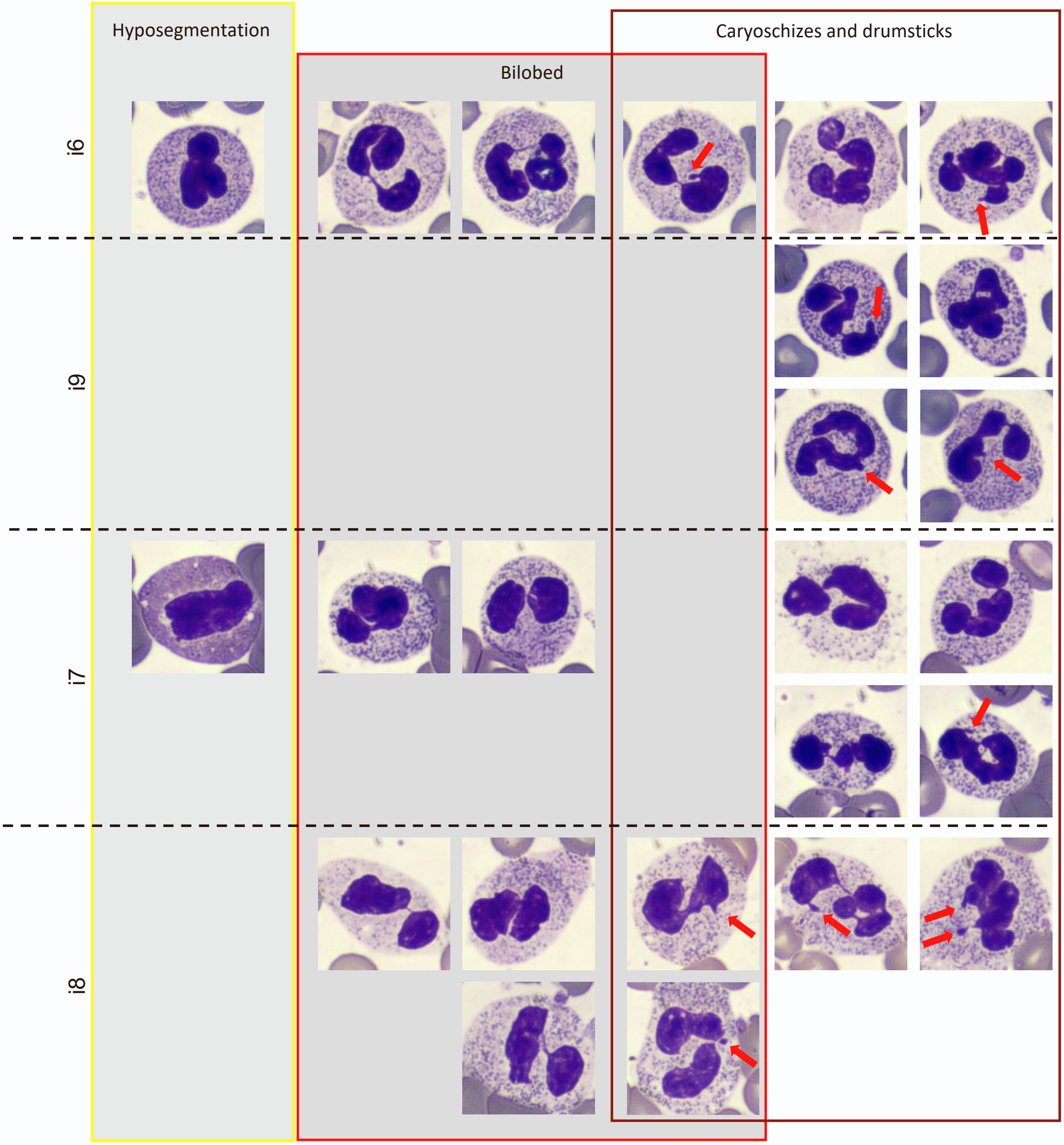

Hyposegmentation
Caryoschizes and drumsticks
Bilobed
i6
i9
i7
i8

B

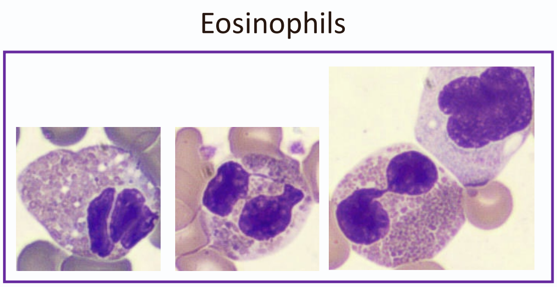

Eosinophils

Figure S3. Pseudo-Pelger-Huet anomaly identified in additional *TMEM147* individuals. (A) Chromatin clumping, hyposegmentation, bilobed nuclei, carioschizes, and drumsticks (red arrows) observed in neutrophils of i6, i7, i8, and i9 (F5-IV-2, F6-V-2, 5 and 6 respectively).(B) Sparse eosinophils showing nuclear anomalies observed in *TMEM147* individuals.

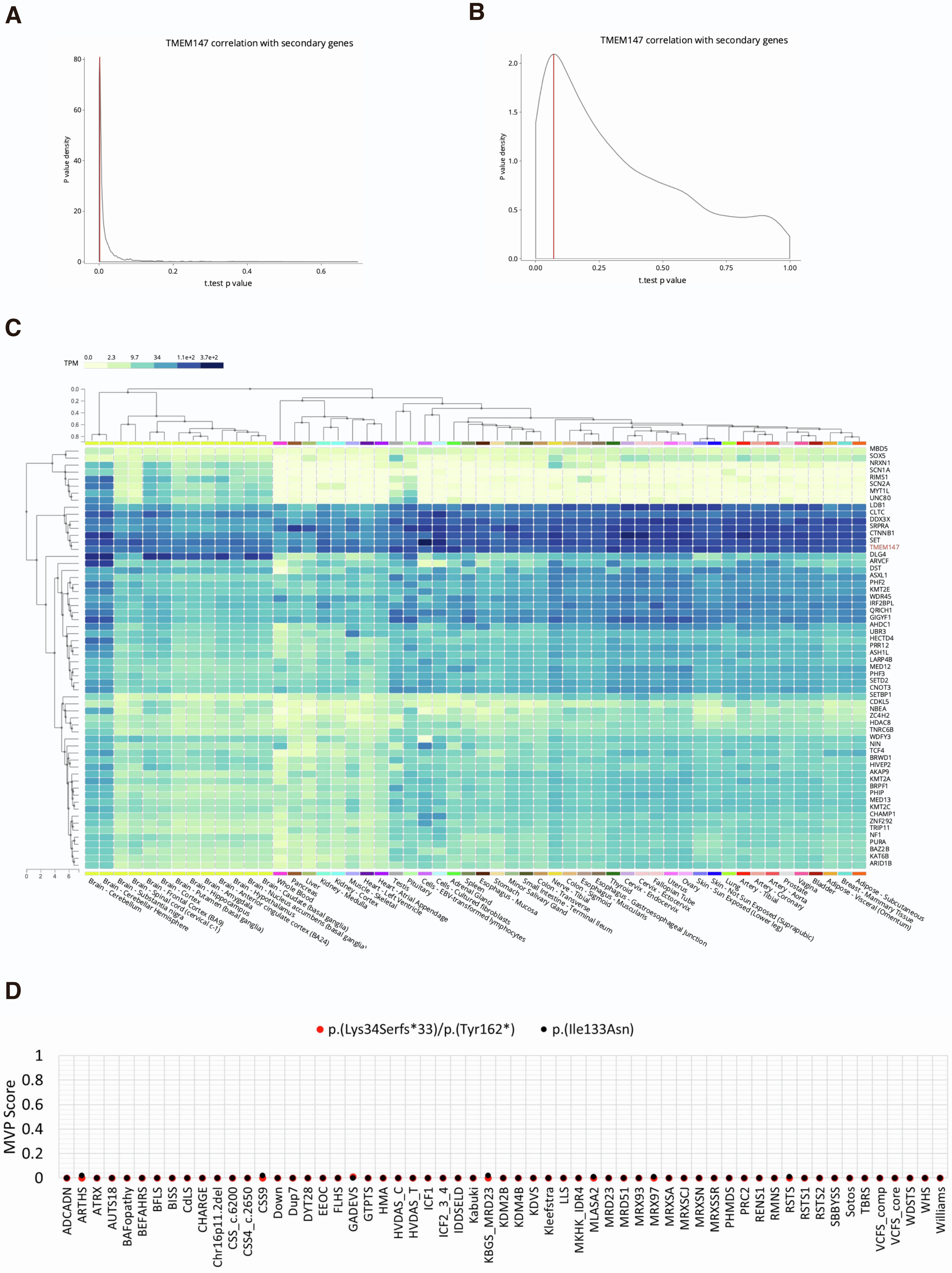

A
TMEM147 correlation with secondary genes
P value density
t.test p value
B
TMEM147 correlation with secondary genes
P value density
t.test p value
C
TPM
0.0 2.3 9.7 34 1.1e+2 3.7e+2
MBD5
SOX5
NRXN1
SCN1A
RIMS1
SCN2A
MYT1L
UNC80
LDB1
CLTC
DDX3X
SRPRA
CTNNB1
SET
TMEM147
DLG4
ARVCF
DST
ASXL1
PHF2
KMT2E
WDR45
IRF2BPL
QRICH1
GIGYF1
AHDC1
UBR3
HECTD4
PRR12
ASH1L
LARP4B
MED12
PHF3
SETD2
CNOT3
SETBP1
CDKL5
NBEA
ZC4H2
HDAC8
TNRC6B
WDFY3
NIN
TCF4
BRWD1
HIVEP2
AKAP9
KMT2A
BRPF1
PHIP
MED13
KMT2C
CHAMP1
ZNF292
TRIP11
NF1
PURA
BAZ2B
KAT6B
ARID1B
Brain - Cerebellum
Brain - Cerebellar Hemisphere
Brain - Substantia nigra
Brain - Spinal cord (cervical c-1)
Brain - Frontal Cortex (BA9)
Brain - Cortex
Brain - Putamen (basal ganglia)
Brain - Hippocampus
Brain - Amygdala
Brain - Anterior cingulate cortex (BA24)
Brain - Hypothalamus
Brain - Nucleus accumbens (basal ganglia)
Brain - Caudate (basal ganglia)
Whole Blood
Pancreas
Liver
Kidney - Medulla
Kidney - Cortex
Muscle - Skeletal
Heart - Left Ventricle
Heart - Atrial Appendage
Testis
Pituitary
Cells - EBV-transformed lymphocytes
Cells - Cultured fibroblasts
Adrenal Gland
Spleen
Esophagus - Mucosa
Stomach
Minor Salivary Gland
Small Intestine - Terminal Ileum
Colon - Transverse
Nerve - Tibial
Colon - Sigmoid
Esophagus - Muscularis
Thyroid
Esophagus - Gastroesophageal Junction
Cervix - Endocervix
Cervix - Ectocervix
Fallopian Tube
Uterus
Ovary
Skin - Sun Exposed (Lower leg)
Skin - Not Sun Exposed (Suprapubic)
Lung
Artery - Tibial
Artery - Coronary
Artery - Aorta
Prostate
Vagina
Bladder
Adipose - Visceral (Omentum)
Breast - Mammary Tissue
Adipose - Subcutaneous
D
p.(Lys34Serfs*33)/p.(Tyr162*)
p.(Ile133Asn)
MVP Score
ADCADN
ARTHS
ATRX
AUTS18
BAFopathy
BEFAHRS
BFLS
BISS
CdLS
CHARGE
Chr16p11.2del
CSS_c.6200
CSS4_c.2650
CSS9
Down
Dup7
DYT28
EEOC
FLHS
GADEVS
GTPTS
HMA
HVDAS_C
HVDAS_T
ICF1
ICF2_3_4
IDDSELD
Kabuki
KBGS_MRD23
KDM2B
KDM4B
KDVS
Kleefstra
LLS
MKHK_IDR4
MLASA2
MRD23
MRD51
MRX93
MRX97
MRXSA
MRXSCJ
MRXSN
MRXSSR
PHMDS
PRC2
RENS1
RMNS
RSTS
RSTS1
RSTS2
SBBYSS
Sotos
TBRS
VCFS_comp
VCFS_core
WDSTS
WHS
Williams

Figure S4 ***TMEM147*** **co-expression analysis with neurodevelopmental genes and EpiSign (DNA methylation) analysis of peripheral blood from two cases with variants in** ***TMEM147*****.** *TMEM147* expression profile in different human tissues. (A) The plot shows the distribution of t-test *p*-values for Pearson correlations between *TMEM147* and NDD gene set in brain tissues as obtained by permutation testing. Summit *p*-value = 0.002 [**]. (B) The plot shows the distribution of t-test *p*-values for Pearson correlations between *TMEM147* and NDD genes in all tissues as obtained by permutation testing. Summit p-value = 0.071 [ns]. (C) Heatmap showing TMP (transcripts *per* kilobase million) values from GTEx database for *TMEM147* and its most correlated NDD genes (>|0.3|). Gene expression profiles are clustered by gene (vertical clustering) and by tissue (horizontal clustering). (D) A multi-class supervised classification system capable of discerning between multiple episignatures by generating a probability score (MVP) for each episignature. A positive score is typically greater than 0.5 and the presentation of all scores as 0.02 or less indicate a profile more similar to controls for each episignature assessed.

Table S1. Genetic variants identified in the *TMEM147* cohort

Individual Family	Genomic coordinates (GRCh37; hg19) chr19:	Transcript NM_032635.4	Protein	Familial segregation	GnomAD frequency	RASopathy or Chromatinopathy indication	*In silico* predictions
i1 – F1	g.36036812_36036830del<br>g.36038077C>G	c.100_118del<br>c.486C>G	p.Lys34Serfs*33<br>p.Tyr162*	Compound heterozygous	3.99e-6<br>0	RASopathy	
i2 – F2	g.36036704C>G<br>g.36037921dup	c.63C>G<br>c.419dup	p.Tyr21*<br>p.Asn140Lysfs*21	Compound heterozygous	0<br>0	none	
i3 – F3	g.36037900T>A	c.398T>A	p.Ile133Asn	homozygous	0	Chromatinopathy (Cornelia de Lange)	CADD 28<br>PP2 0.98<br>GERP 5.11<br>REVEL 0.472<br>3Cnet 0.859
i4 – F4	g.36037900T>A	c.398T>A	p.Ile133Asn	homozygous	0		CADD 28<br>PP2 0.98<br>GERP 5.11<br>REVEL 0.472<br>3Cnet 0.859
i5 – F4	g.36037900T>A	c.398T>A	p.Ile133Asn	homozygous	0		CADD 28<br>PP2 0.98<br>GERP 5.11<br>REVEL 0.472<br>3Cnet 0.859
i6 – F5	g.36037900T>A	c.398T>A	p.Ile133Asn	homozygous	0		CADD 28<br>PP2 0.98<br>GERP 5.11<br>REVEL 0.472<br>3Cnet 0.859
i7 – F6	g.36037900T>A	c.398T>A	p.Ile133Asn	homozygous	0		CADD 28<br>PP2 0.98<br>GERP 5.11<br>REVEL 0.472<br>3Cnet 0.859
i8 – F6	g.36037900T>A	c.398T>A	p.Ile133Asn	homozygous	0		CADD 28<br>PP2 0.98<br>GERP 5.11<br>REVEL 0.472<br>3Cnet 0.859
i9 – F6	g.36037900T>A	c.398T>A	p.Ile133Asn	homozygous	0		CADD 28<br>PP2 0.98<br>GERP 5.11<br>REVEL 0.472<br>3Cnet 0.859
i10 – F6	g.36037900T>A	c.398T>A	p.Ile133Asn	homozygous	0		CADD 28<br>PP2 0.98<br>GERP 5.11<br>REVEL 0.472<br>3Cnet 0.859
i11 – F7	g.36037900T>A	c.398T>A	p.Ile133Asn	homozygous	0		CADD 28<br>PP2 0.98<br>GERP 5.11<br>REVEL 0.472<br>3Cnet 0.859
i12 – F8	g.36037715G>A	c.344+5G>A	p.?	homozygous	1.41e-5		
i13 – F8	g.36037715G>A	c.344+5G>A	p.?	homozygous	1.41e-5		
i14 – F8	g.36037715G>A	c.344+5G>A	p.?	homozygous	1.41e-5		
i15 – F9	g.36037846G>T	c.345-1G>T	p.?	homozygous	0		
i16 – F10	g.36036660G>C	c.19G>C	p.Gly7Arg	homozygous	5.01e-5	RASopathy	CADD 27.9<br>PP2 1.0<br>GERP 5.4<br>REVEL 0.345<br>3Cnet 0.056
i17 – F10	g.36036660G>C	c.19G>C	p.Gly7Arg	homozygous	5.01e-5	RASopathy	CADD 27.9<br>PP2 1.0<br>GERP 5.4<br>REVEL 0.345<br>3Cnet 0.056

i18 – F11	g.36036660G>C	c.19G>C	p.Gly7Arg	homozygous	5.01e-5	none	CADD 27.9<br>PP2 1.0<br>GERP 5.4<br>REVEL 0.345<br>3Cnet 0.056
i19 – F12	g.36037443_36037452del	c.169_172del	p.Phe57Profs*15	homozygous	0		
i20 – F13	g.36037892G>A	c.390G>A	p.Trp130*	homozygous	0		
i21 – F14	g.36038131_36038134dup	c.540_543dup	p.Val182Leufs*66	homozygous	1.99e-5		
i22 – F15	g.36038087C>T	c.496C>T	p.Arg166Trp	homozygous	0		CADD 25.9<br>PP2 0.99<br>GERP 5.64<br>REVEL 0.206<br>3Cnet 0.471
i23 – F15	g.36038087C>T	c.496C>T	p.(Arg166Trp)	homozygous	0		CADD 25.9<br>PP2 0.99<br>GERP 5.64<br>REVEL 0.206<br>3Cnet 0.471

[a] PP2: PolyPhen2

[b] 3Cnet cutoff is 0.75

Table S2 Extended clinical features of the *TMEM147* cohort

Individual<br>Family	i1<br>F1	i2<br>F2	i3<br>F3	i4<br>4	i5<br>F4	i6<br>F5	i7<br>F6	i8<br>F6	i9<br>F6	i10<br>F6	i11<br>F7
Year of birth	2004	2008	2020	2005	2016	2016	2013	2019	2016	2018	2012
Sex	female	female	female	male	male	female	female	male	female	male	male
Measurements at birth	-1.12/+0.35/-1.23	UN	-6/-6/-5	-0.98/-1/-1.7	-0.5/+0.3/+05	-0.5/-0.08/-1.11	-4/-1.8/+0.1	-4.3/-1/-0.89	-3.2/-0.88/-1.4	-4/-1.3/-1.7	-1.9/-1.2/-1.5
Age at last follow-up	16y	8y	2y	13y	4y 3m	6y 6m	9y 1m	2y 11m	6y	3y 2m	2y 6m
Measurements at last follow-up	-1.5/+2.25/+1.5	-0.69/-0.5/UN	UN	-1.7/+1.3/+2.4	-2.1/+1/-1.2	-1/-1/-1.5	-0.8/-1.5/-2.3	-1.7/-1.8/-1.6	-1.2/-2.3/-1	-1.6/-1.6/-1.6	-1.8/-1/-4.6
Motor delay	+	+	+	+	+	+	+	+	+	+	+
Walking age	2y	5y	not acquired	4y	not acquired	3y	4y	not acquired	5y	not acquired	not acquired
Intellectual disability	+	+	UN	+	+	+	+	+	+	+	+
Severity	UN	severe	UN	IQ: 50	IQ: 45	IQ: 53	IQ: 48	IQ: 35-40	IQ: 45	IQ: 35	IQ: 20-25
Language delay	+	+	+	+	+	+	+	+	+	+	+
Speech ability upon last examination	single words	no speech	babbling	short sentences	no speech	single words	single words	no speech	single words	no speech	no speech
Behavioral problems	outbursts of anger, hyperactivity, self-injury	aggressivity, frustration intolerance self-injury	-	repetitive movements, outbursts of anger, hyperactivity	outbursts of anger, hyperactivity, anxiety	outbursts of anger, hyperactivity, hair-eating	outbursts of anger, hyperactivity	outbursts of anger, anxiety	outbursts of anger	outbursts of anger, anxiety	outbursts of anger, excessive crying
Neurological features	synkinesis with mirror movements of the hands (childhood), hypotonia	-	general hypotonia	-	-	-	-	-	-	bilateral severe sensorineural hearing loss	tonic seizures
Facial features	coarse facies, thick curly hair, hypertelorism, low-set ears, epicanthus large mouth, thick lower lip, sparse eyebrows, broad depressed nasal root	coarse facies, thin upper lip, epicanthus synophrys	broad depressed nasal root, synophrys, epicanthus, tented mouth	prominent forehead, broad nasal root, flat nasal tip, large tented mouth, long philtrum low set ears	prominent forehead, hypertelorism, down-slanting palpebral fissures, depressed broad nasal root, long philtrum, thin upper lip, everted lower lip, low set ears	coarse facies, elongated face, sparse hair, prominent forehead, depressed nasal root, down-slanting palpebral fissures, long philtrum, tented big mouth, low set ears	coarse facies, prominent forehead, sparse eyebrows, prominent nasal root, triangular prominent nose, long smooth philtrum, thin upper lip, low set large ears	coarse facies, prominent forehead, down-slanting palpebral fissures, synophrys depressed nasal root, long smooth philtrum, tented mouth, low set ears	coarse facies, prominent forehead depressed nasal root, broad nasal tip, long smooth philtrum, big mouth, low set ears	coarse facies, prominent forehead, curly hair, down-slanting palpebral fissures, depressed nasal root, long smooth philtrum, tented mouth, low set ears	prominent forehead, sparse eyebrows, depressed nasal root, broad nose tip, smooth philtrum, tented mouth, low set ears
Other Features	kyphosis, fifth finger bilateral clinodactyly, flat feet, valgus feet transverse palmar crease, pubic hair at 5 years 6 months	scoliosis, 5th finger clinodactyly, transverse palmar crease	transverse palmar crease	-	-	-	-	-	-	patent foramen ovale	-

Brain MRI	normal	normal	normal	thin corpus callosum	thin corpus callosum, myelination delay	thin corpus callosum, enlarged lateral ventricles, periventricular white matter T2 hyperintensities	thin corpus callosum, mild prominent verminan folia,	thin corpus callosum, mild prominent vermian folia	thin corpus callosum, enlarged lateral ventricles, prominent vermian folia	thin corpus callosum, enlarged lateral ventricles, white matter hyperintensities, prominent cerebellar folia	thin corpus callosum, hypoplastic lower vermis

Individual<br>Family	i12<br>F8	i13<br>F8	i14<br>F8	i15<br>F9	i16<br>F10	i17<br>F10	i18<br>F11	i19<br>F12	i20<br>F13	i21<br>F14	i22<br>F15	i23<br>F15
Year of birth	1996	2002	2004	2018	2013	2016	2004	2019	2016	2020	2006	2010
Sex	male	female	female	male	female	male	female	male	male	male	male	male
Measurements at birth	within norms	within norms	within norms	UN	within norms	within norms	UN	-1.17/-0.77/-0.9	-1/-0.8/-1.7	-1.17/+1.5/-1.8	-1/-0.5/+0.9	-1.2/-0.4/+1
Age at last follow-up	14y	8y	6y	3y	8y	5y	18y	2 years	4y6m	14m	6y	1y
Measurements at last follow-up	within norms	within norms	within norms	-1.2/0/-2.5	UN	-2/-2/UN	UN	+1/-1/+1	-3/-3/-3	-2/-1/+1	-2/-2/-4.6	-1.7/+0.2/-2.1
Motor delay	+	+	+	+	+	+	+	+	+	+	+	+
Walking age	3y	3y	3y	3y	UN	UN	3y	1.5y	not acquired	not acquired	not acquired	not acquired
Intellectual disability	+	+	+	+	+	+	+	+	+	+	+	too young
Severity	severe	severe	severe	severe	severe	severe	severe	moderate	severe	UN	IQ: 35	too young
Language delay	+	+	+	+	+	+	+	+	+	+	+	UN
Speech ability upon last examination	no speech	single words	no speech	single words	short sentences	single words	single words	single words	single words	babbling	no speech	UN
Behavioral problems	-	-	-	UN	outbursts of anger	outbursts of anger, self-injury	Anxiety, hyperactivity	-	outbursts of anger, aggressivity	-	outburst of anger, autistic traits	-
Neurological features	-	-	-	-	-	general hypotonia	-	-	-	-	tonic seizures	-
Facial features	coarse facies, synophrys, epicanthus, long smooth philtrum, elongated face	coarse facies, synophrys, epicanthus, broad nasal root, smooth philtrum, big mouth, thin upper lip	coarse facies, synophrys, epicanthus, broad nasal root, smooth philtrum big mouth	marked epicantus, large ears, tented mouth, short philtrum inverted nasal tip	coarse facies, hypertelorism, sparse hair, synophyrs, full lips	UN	long face, exophoria, prognathism, everted lower lip, anteverted floppy ears	coarse facies, prominent forehead epicanthus, thin upper lip	coarse facies, synophrys, marked epicanthus, flat nasal tip, long smooth philtrum big tented mouth, elongated face	coarse facies, prominent forehead, sparse eyebrows, epicanthus, depressed nasal root, long philtrum tented mouth	prominent forehead, depressed nasal root, broad nose tip, prominent nares, short philtrum, tented mouth, low set ears	prominent forehead, depressed nasal root, broad nose tip, prominent nares, long philtrum, tented mouth, low set ears
Other features	elbows arthrogryposis	ichthyosis	ichthyosis	atrial septal defect, large patent ductus arteriosus			kyphoscoliosis, pes planus, long and slender fingers and toes, late puberty	patent foramen ovale	-	-	-	-
Brain MRI	UN	UN	UN	normal	UN	UN	posterior white matter hyperintensity	reduced white matter volume	Periventricular and occipital white matter T2 hyperintensities, myelination delay	normal	thin corpus callosum mild frontoparietal cortical atrophy, wide interhemispheric fissure, bilateral mild deep sylvian fissure,	thin corpus callosum, mild frontoparietal cortical atrophy, wide interhemispheric fissure, bilateral mild deep sylvian fissure,

											periventricular and occipital white matter T2 hyperintensities	periventricular white matter T2 hyperintensities

[a] y: years
[b] m: months
[c] na: not acquired
[d] UN: unknown
[e] ty: too young
[f] se: severe
[g] mo moderate
[h] sw: single words
[i] ns: no speech
[j] ba: babbling
[k] ss: short sentences
[l] Measurements were expressed as standard deviation scores of weight, height and head circumference respectively

Supplemental material and methods

Protein modelling and *in silico* analysis

The ER translocon structure[1] was fetched from the Protein database (PDB id:6W6L) and displayed using PyMol version 2.5.2 (https://pymol.org/). All proteins were displayed as cartoon views. When necessary, surface was added to the polypeptide chains with the transparency set to 0.3. The PyMol Wizard tools was used to model the different mutations inside TMEM147 sequence. For each mutation studied, the WT amino acid was first visualized as sticks inside the cartoon representation, leaving only the side chain colored. Surface view was generated as mentioned above. Then the amino acid was mutated accordingly and made visible in the same manner. When steric clashes were reported for the possible rotamers by the wizard tool in PyMol, the least severe situation was selected. A putative view was modeled and oriented to match the WT situation. The alignment in Figure S1 was obtained with the TEXshade package in LaTeX.[2] The plot was generated based on data found both in the UniProt and the AlphaFold databases using the TEXtopo package in LaTeX.[3]

Reagents

Dulbecco's modified Eagle's medium (DMEM), fetal bovine serum (FBS), phosphate-buffered saline (PBS), glutamine and antibiotics were obtained from Euroclone (Wetherby, UK). Polyethylenimine (PEI) transfection reagent was purchased from Polysciences (Warrington, PA). Protease and phosphatase inhibitor cocktails, cycloheximide (CHX) and bafilomycin A1 were from Sigma-Aldrich (St. Luis, MO). Trans-Blot Turbo Transfer Packs were obtained from Bio-Rad Laboratories (Hercules, CA). ECL Western Blotting Detection reagents were from Pierce Biotechnology (Rockford, IL). QuiKChange II Site-Directed Mutagenesis kit was purchased from Stratagene (La Jolla, CA). pcDNA6.2/V5-HisA eukaryotic expression vector,

and Hoechst 33342 staining solution were from Invitrogen (Carlsbad, CA). The following antibodies were used: mouse monoclonal anti-V5 (R96025, Invitrogen); mouse monoclonal anti-GAPDH (SC-32233, Santa Cruz, Dallas, CA); rabbit polyclonal anti-calnexin (ab 10286, Abcam, Cambridge, UK); mouse monoclonal anti-CKAP4 (anti-CLIMP-63; ABS669-0100 Enzo Life Sciences, Farmingdale, NY, USA); rabbit polyclonal anti-RTN4 (anti-NOGO; ab47085 Abcam - Cambridge, UK); rabbit polyclonal anti-LBR (HPA062236 Sigma Aldrich, St. Louis, Missouri, USA); anti-actin horseradish peroxidase-conjugated anti-mouse (31450, Invitrogen); goat anti-mouse conjugated to Alexa Fluor 594 (A-11020, Invitrogen); goat anti-mouse conjugated to Alexa Fluor 568 (A-11031, Invitrogen) for fibroblast cell staining; goat anti-rabbit conjugated to Alexa Fluor 488 (A-11070, Invitrogen for COS-1 cell staining or A-11034, Invitrogen for fibroblast cell staining). Actin staining was obtained using ActinRed^{TM} 555 ReadyProbes^{TM} reagent (R37112, Invitrogen).

Constructs

The entire coding sequence of WT TMEM147 was cloned into the pcDNA6.2/V5-HisA eukaryotic expression vector. Mutant TMEM147 constructs carrying the p.Gly7Arg, p.Ile133Asn or p.Arg166Trp amino acid substitutions were generated by PCR-based site-directed mutagenesis using the QuiKChange II Site-Directed Mutagenesis Kit.[4] The identity of each construct was verified by bidirectional sequencing (ABI BigDye terminator Sequencing Kit v3.1, SeqStudio Genetic Analyzer; Applied Biosystems, Foster City, CA).

Cell culture, transfection, and inhibitor treatment

COS-1 cells were cultured in DMEM medium supplemented with 10% heat-inactivated FBS, 1% L-glutamine, and antibiotics (37 °C, humidified atmosphere containing 5% CO_2). Subconfluent cells were transfected using the PEI transfection reagent according to the

manufacturer's instructions. Cells were treated with cycloheximide (CHX) (10 μg/mL) or bafilomycin A1 (200 nM) to analyze protein stability and degradation.

Human primary fibroblasts (healthy control and patient-derived fibroblasts) were cultured in DMEM High Glucose medium 4.5 g/L (HyClone Thermo Scientific, Waltham, MA) supplemented with 10% Fetal Bovine Serum (FBS, Thermo Scientific) and 1% ZellShield (commercial mixture of antibiotics and anti-mycoplasmas reagents) (Minerva, Biovalley, France). Cells were cultured at 37 °C in a humidified 5% CO_2 atmosphere.

Cell homogenate and immunoblotting analyses

Cells were lysed in radio-immune precipitation assay (RIPA) buffer, pH 8.0, supplemented with protease and phosphatase inhibitors. Lysates were centrifuged at 16000g for 20 min at 4 °C, and supernatant protein concentration was determined by Bradford assay, using BSA as standard. Western blotting (WB) and densitometric analyses were performed as previously described.[4,5]

Immunohistochemistry and cell staining

Fibroblast cells were seeded 24 hours before labeling in 6-well plates containing coverslips, 2.10^5 cells per well. Cells were washed with cold PBS and fixed with 4% formaldehyde for 20 min at room temperature. The cells were then incubated with NH_4Cl (50mM) solution for 10 min, followed by permeabilization with 0.3% PBS-Triton solution for 7 min, still at room temperature. Saturation was performed with 1% PBS-BSA, 0.1% Tween 20, 15 min at room temperature. The primary antibody was incubated at 4°C overnight, diluted in blocking buffer. The next day, after 3 washes with cold PBS, the 1/1000th secondary antibody was added for 45 min. After 3 washes, nuclei were stained using Hoechst, washed and the mount is made on slides for microscope analysis.

Fibroblast cell lines were stained with May Grunwald-Giemsa (MGG) staining method to inspect nucleus morphology.

Imaging

COS-1 cells ($15x10^3$) were seeded on glass coverslips and transfected with the various constructs for 24 h. Cells were fixed with 4% paraformaldehyde (30 min, 4 °C) and permeabilized (0.5% Triton X-100, 10 min, room temperature [RT]). Cells were stained with a mouse monoclonal anti-V5 followed by goat anti-mouse Alexa Fluor 594, and then with a rabbit polyclonal anti-calnexin followed by goat anti-rabbit Alexa Fluor 488. Finally, nuclei were stained with Hoechst 33342 solution and glass coverslips were mounted on the microscope slides by using PBS-glycerol buffer. Confocal laser scanning microscopy analysis was performed by a Leica TCS-SP8X (Leica Microsystems) equipped with a 405 nm diode laser and a white light laser (WLL) source using excitation spectral laser lines at 488 nm and 594 nm. COS-1 cells stained only with the fluorochrome-conjugated secondary antibody were used to set up acquisition parameters. Signals from different fluorescent probes were taken in sequential scanning mode. Image processing used Adobe Photoshop 7.0 software (Adobe Systems Incorporated). Microscopic studies in patient-derived primary fibroblasts were performed at the GAD laboratory with a Zeiss AX10. Image analyses and quantifications were performed with ImageJ software. Quantification was obtained by calculating a Correlated Total Cell Fluorescence (CTCF) parameter with the following equation:

CTCF = integrated density – (area of selected cell x mean fluorescence of background).[6]

Blood smears were stained using standard May-Grünwald-Giemsa stain and then analyzed on a Sysmex XN haematology analyser coupled with a DI camera.

Statistical analysis

Statistical analyses were performed using GraphPad Prism software (GraphPad Software, La Jolla, CA) with a two-way ANOVA followed by Tukey's multiple comparison test or a one sample Wilcoxon test.

Transcriptome analysis

ARCHS4, a database with thousands of standardized RNA-Seq datasets (ARCHS4: https://maayanlab.cloud/archs4),[7] and *Correlation AnalyzeR* tool,[8] were used to calculate co-expression correlations with respect to tissue and disease (cancer/normal) condition. Pearson correlation between the *TMEM147* expression pattern and those from a previously defined set of genes mutated in neurodevelopmental disorders[9] in brain tissues was assessed by *Correlation AnalyzeR* R package. The selected gene set included genes with at least three *de novo bona fide* loss-of-function (LoF) mutations in unrelated individuals with NDDs and no *de novo* LoF mutation in apparently healthy subjects from the denovo-db v.1.6.1 database (denovo-db: https://denovo-db.gs.washington.edu/denovo-db/),[10] and nonredundant *de novo* mutations from Satterstrom et al. (2020).[11] Statistical significance of correlations was evaluated by permutation t-test comparing the selected NDD genes to a list of random genes of the same size. The *p*-value distribution constructed during permutation testing approximates the likelihood that *TMEM147* and the gene list are more correlated than expected by random association.[8] The GTEx database (GTEx: https://www.gtexportal.org/home/) was used to independently test the brain-specific correlation of *TMEM147* with the most correlated genes (>|0.3|) of the NDD gene set.

DNA Methylation Data Analysis

Methylation analysis was performed with version 3 of the clinically validated EpiSign assay as previously described.[12–15] Briefly, methylated and unmethylated signal intensity generated from the EPIC array was imported into R 3.5.1 for normalization, background correction and filtering. Beta values ranging from 0 (no methylation) to 1 (complete methylation) were calculated as a measure of methylation level and processed through the established support vector machine (SVM) classification algorithm for EpiSign disorders. The EpiSign Knowledge Database composed of over 5000 methylation profiles from reference disorder-specific and unaffected control cohorts was utilized by the classifier to generate disorder specific methylation variant pathogenicity (MVP) scores. MVP scores are a measure of prediction confidence for each disorder, ranging from 0 (discordant) to 1 (highly concordant). A positive classification typically generates MVP scores greater than 0.5 and these scores in combination with assessment of hierarchical clustering and multidimensional scaling are used in generating the final matched EpiSign result.

Supplemental references

1. McGilvray, P.T., Anghel, S.A., Sundaram, A., Zhong, F., Trnka, M.J., Fuller, J.R., Hu, H., Burlingame, A.L., and Keenan, R.J. (2020). An ER translocon for multi-pass membrane protein biogenesis. ELife *9*, e56889.

2. Beitz, E. (2000). TeXshade: shading and labeling of multiple sequence alignments using LaTeX2e. Bioinformatics *16*, 135–139.

3. Beitz, E. (2000). TeXshade: shading and labeling of multiple sequence alignments using LaTeX2e. Bioinformatics *16*, 135–139.

4. Motta, M., Fasano, G., Gredy, S., Brinkmann, J., Bonnard, A.A., Simsek-Kiper, P.O., Gulec, E.Y., Essaddam, L., Utine, G.E., Guarnetti Prandi, I., et al. (2021). SPRED2 loss-of-function causes a recessive Noonan syndrome-like phenotype. The American Journal of Human Genetics *108*, 2112–2129.

5. Motta, M., Pannone, L., Pantaleoni, F., Bocchinfuso, G., Radio, F.C., Cecchetti, S., Ciolfi, A., Di Rocco, M., Elting, M.W., Brilstra, E.H., et al. (2020). Enhanced MAPK1 Function Causes a

Neurodevelopmental Disorder within the RASopathy Clinical Spectrum. The American Journal of Human Genetics *107*, 499–513.

6. Abaza, M.S.I., Afzal, M., Al-Attiyah, R.J., and Guleri, R. (2016). Methylferulate from Tamarix aucheriana inhibits growth and enhances chemosensitivity of human colorectal cancer cells: possible mechanism of action. BMC Complement Altern Med *16*, 384.

7. Lachmann, A., Torre, D., Keenan, A.B., Jagodnik, K.M., Lee, H.J., Wang, L., Silverstein, M.C., and Ma'ayan, A. (2018). Massive mining of publicly available RNA-seq data from human and mouse. Nat Commun *9*, 1366.

8. Miller, H.E., and Bishop, A.J.R. (2021). Correlation AnalyzeR: functional predictions from gene co-expression correlations. BMC Bioinformatics *22*, 206.

9. Radio, F.C., Pang, K., Ciolfi, A., Levy, M.A., Hernández-García, A., Pedace, L., Pantaleoni, F., Liu, Z., de Boer, E., Jackson, A., et al. (2021). SPEN haploinsufficiency causes a neurodevelopmental disorder overlapping proximal 1p36 deletion syndrome with an episignature of X chromosomes in females. The American Journal of Human Genetics *108*, 502–516.

10. Turner, T.N., Yi, Q., Krumm, N., Huddleston, J., Hoekzema, K., F. Stessman, H.A., Doebley, A.-L., Bernier, R.A., Nickerson, D.A., and Eichler, E.E. (2017). denovo-db: a compendium of human *de novo* variants. Nucleic Acids Res *45*, D804–D811.

11. Satterstrom, F.K., Kosmicki, J.A., Wang, J., Breen, M.S., De Rubeis, S., An, J.-Y., Peng, M., Collins, R., Grove, J., Klei, L., et al. (2020). Large-Scale Exome Sequencing Study Implicates Both Developmental and Functional Changes in the Neurobiology of Autism. Cell *180*, 568-584.e23.

12. Levy, M.A., McConkey, H., Kerkhof, J., Barat-Houari, M., Bargiacchi, S., Biamino, E., Bralo, M.P., Cappuccio, G., Ciolfi, A., Clarke, A., et al. (2022). Novel diagnostic DNA methylation episignatures expand and refine the epigenetic landscapes of Mendelian disorders. Human Genetics and Genomics Advances *3*, 100075.

13. Aref-Eshghi, E., Kerkhof, J., Pedro, V.P., Barat-Houari, M., Ruiz-Pallares, N., Andrau, J.-C., Lacombe, D., Van-Gils, J., Fergelot, P., Dubourg, C., et al. (2020). Evaluation of DNA Methylation Episignatures for Diagnosis and Phenotype Correlations in 42 Mendelian Neurodevelopmental Disorders. The American Journal of Human Genetics *106*, 356–370.

14. Aref-Eshghi, E., Bend, E.G., Colaiacovo, S., Caudle, M., Chakrabarti, R., Napier, M., Brick, L., Brady, L., Carere, D.A., Levy, M.A., et al. (2019). Diagnostic Utility of Genome-wide DNA Methylation Testing in Genetically Unsolved Individuals with Suspected Hereditary Conditions. The American Journal of Human Genetics *104*, 685–700.

15. Sadikovic, B., Levy, M.A., Kerkhof, J., Aref-Eshghi, E., Schenkel, L., Stuart, A., McConkey, H., Henneman, P., Venema, A., Schwartz, C.E., et al. (2021). Clinical epigenomics: genome-wide DNA methylation analysis for the diagnosis of Mendelian disorders. Genetics in Medicine *23*, 1065–1074.
