# Supplementary material for: Bi-allelic loss-of-function variants in TMEM147 cause moderate to profound intellectual disability with facial dysmorphism and pseudo-Pelger-Huët anomaly
Source: Am J Hum Genet. 2022 Aug 30;109(10):1909–22. doi: 10.1016/j.ajhg.2022.08.008 (PMC9606387; doi:10.1016/j.ajhg.2022.08.008)
Supplement: Document S2. Article plus supplemental information [file mmc2.pdf]

Bi-allelic loss-of-function variants in *TMEM147* cause moderate to profound intellectual disability with facial dysmorphism and pseudo-Pelger-Huët anomaly

Graphical abstract

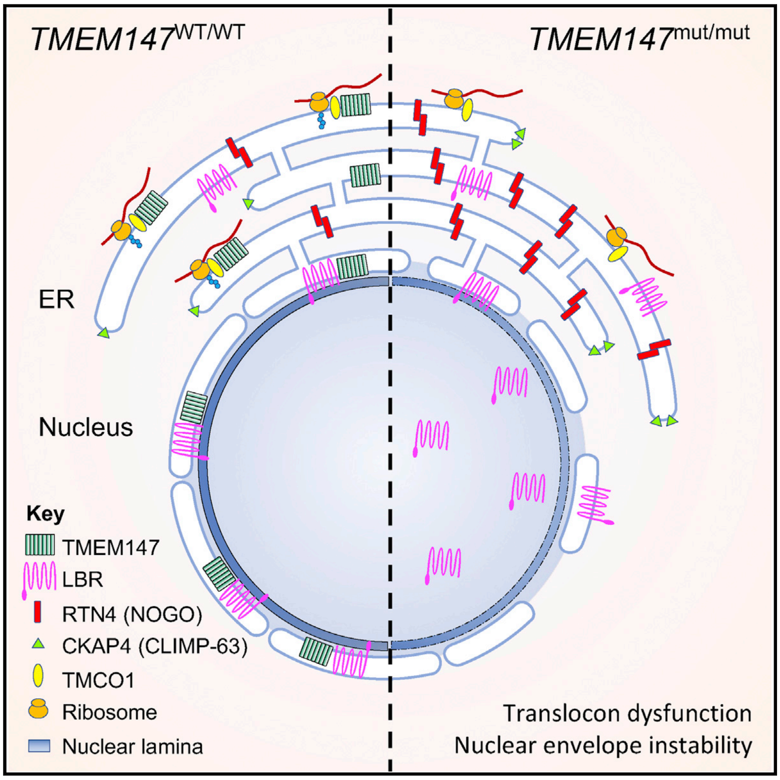

Authors

Quentin Thomas, Marialetizia Motta, Thierry Gautier, ..., Jérôme Govin, Marco Tartaglia, Antonio Vitobello

Correspondence

quentin.thomas@chu-dijon.fr (Q.T.),
antonio.vitobello@u-bourgogne.fr (A.V.)

TMEM147 plays an important role in protein localization and biogenesis. We discovered that individuals with bi-allelic *TMEM147* loss-of-function variants show a neurodevelopmental disorder associated with facial dysmorphism and pseudo-Pelger-Huët anomaly. In primary cell lines, we observed nuclear envelope instability accompanied by lamin B receptor mislocalization and ER-translocon dysfunction.

Thomas et al., 2022, The American Journal of Human Genetics *109*, 1909–1922
October 6, 2022 © 2022 The Authors.
https://doi.org/10.1016/j.ajhg.2022.08.008

CellPress

REPORT

Bi-allelic loss-of-function variants in *TMEM147* cause moderate to profound intellectual disability with facial dysmorphism and pseudo-Pelger-Huët anomaly

Quentin Thomas,[1,39,*] Marialetizia Motta,[2,39] Thierry Gautier,[3,39] Maha S. Zaki,[4,5,39] Andrea Ciolfi,[2] Julien Paccaud,[1] François Girodon,[1,6] Odile Boespflug-Tanguy,[7,8] Thomas Besnard,[9,10] Jennifer Kerkhof,[11] Haley McConkey,[11,12] Aymeric Masson,[1] Anne-Sophie Denommé-Pichon,[1,13] Benjamin Cogné,[9,10] Eva Trochu,[9] Virginie Vignard,[10] Fatima El It,[1] Lance H. Rodan,[14,15] Mohammad Ayman Alkhateeb,[16] Rami Abou Jamra,[17] Laurence Duplomb,[1] Emilie Tisserant,[1] Yannis Duffourd,[1] Ange-Line Bruel,[1,13] Adam Jackson,[18,19] Siddharth Banka,[18,19] Meriel McEntagart,[20] Anand Saggar,[20,21] Joseph G. Gleeson,[22,23] David Sievert,[22] Hyunwoo Bae,[24] Beom Hee Lee,[24] Kisang Kwon,[25] Go Hun Seo,[25] Hane Lee,[25] Anjum Saeed,[26] Nadeem Anjum,[26] Huma Cheema,[26] Salem Alawbathani,[27] Imran Khan,[27] Jorge Pinto-Basto,[27] Joyce Teoh,[28] Jasmine Wong,[28] Umar Bin Mohamad Sahari,[28]

(Author list continued on next page)

Summary

The transmembrane protein TMEM147 has a dual function: first at the nuclear envelope, where it anchors lamin B receptor (LBR) to the inner membrane, and second at the endoplasmic reticulum (ER), where it facilitates the translation of nascent polypeptides within the ribosome-bound TMCO1 translocon complex. Through international data sharing, we identified 23 individuals from 15 unrelated families with bi-allelic *TMEM147* loss-of-function variants, including splice-site, nonsense, frameshift, and missense variants. These affected children displayed congruent clinical features including coarse facies, developmental delay, intellectual disability, and behavioral problems. *In silico* structural analyses predicted disruptive consequences of the identified amino acid substitutions on translocon complex assembly and/or function, and *in vitro* analyses documented accelerated protein degradation via the autophagy-lysosomal-mediated pathway. Furthermore, TMEM147-deficient cells showed CKAP4 (CLIMP-63) and RTN4 (NOGO) upregulation with a concomitant reorientation of the ER, which was also witnessed in primary fibroblast cell culture. LBR mislocalization and nuclear segmentation was observed in primary fibroblast cells. Abnormal nuclear segmentation and chromatin compaction were also observed in approximately 20% of neutrophils, indicating the presence of a pseudo-Pelger-Huët anomaly. Finally, co-expression analysis revealed significant correlation with neurodevelopmental genes in the brain, further supporting a role of TMEM147 in neurodevelopment. Our findings provide clinical, genetic, and functional evidence that bi-allelic loss-of-function variants in *TMEM147* cause syndromic intellectual disability due to ER-translocon and nuclear organization dysfunction.

Intellectual disability (ID) is defined as a significant impairment in intellectual and adaptive functioning with onset during the developmental period and encompasses a wide panel of neurodevelopmental disorders of variable severity and presentation.[1] With the growing use of massively parallel sequencing technologies, hundreds of

[1]UMR1231 GAD, Inserm, Université Bourgogne-Franche Comté, Dijon, France; [2]Genetics and Rare Diseases Research Division, Ospedale Pediatrico Bambino Gesù, IRCCS, 00146 Rome, Italy; [3]University Grenoble Alpes, Inserm, CNRS, Institute for Advanced Biosciences, 38000 Grenoble, France; [4]Clinical Genetics Department, Human Genetics and Genome Research Institute, National Research Centre, Cairo, Egypt; [5]Armed Forces College of Medicine, Cairo, Egypt; [6]Biology Division, Department of Biological Hematology, Dijon Hospital, 21000 Dijon, France; [7]Université Paris Cité, UMR 1141 NeuroDiderot, Inserm, 75019 Paris, France; [8]Service de Neuropédiatrie, reference center for leukodystrophies, APHP, Hopital Robert Debré, 75019 Paris, France; [9]Service de Génétique Médicale, CHU Nantes, Nantes, France; [10]Université de Nantes, CHU Nantes, CNRS, Inserm, l'Institut du Thorax, 44000 Nantes, France; [11]Verspeeten Clinical Genome Centre, London Health Sciences Centre, London, ON N6A 5W9, Canada; [12]Department of Pathology and Laboratory Medicine, Western University, London, ON N6A 3K7, Canada; [13]Unité Fonctionnelle Innovation en Diagnostic Génomique des Maladies Rares, FHU-TRANSLAD, CHU Dijon Bourgogne, Dijon, France; [14]Division of Genetics and Genomics, Boston Children's Hospital, Harvard Medical School, Boston, MA 02115, USA; [15]Department of Neurology, Boston Children's Hospital, Harvard Medical School, Boston, MA 02115, USA; [16]Women Wellness and Research Center Hamad Medical Corporation, Doha, Qatar; [17]Institute of Human Genetics, University Medical Center, Leipzig, Germany; [18]Division of Evolution, Infection and Genomics, School of Biological Sciences, Faculty of Biology, Medicine and Health, University of Manchester, Manchester, UK; [19]Manchester Centre for Genomic Medicine, St Mary's Hospital, Manchester University NHS Foundation Trust, Health Innovation Manchester, Manchester, UK; [20]Medical Genetics, St George's University Hospitals NHS FT, London SW17 0RE, UK; [21]The Portland Hospital, 205-209 Great Portland St, London W1W 5AH, UK; [22]Department of Neurosciences, University of California, San Diego, La Jolla, CA 92093, USA; [23]Rady Children's Institute for Genomic Medicine, San Diego, La Jolla, CA 92093, USA; [24]Department of Pediatrics, Asan Medical Center Children's Hospital, University of Ulsan College of Medicine, Seoul, Republic of Korea; [25]3billion, Inc, Seoul, South Korea; [26]Children's Hospital and University of Child Health Lahore, Lahore, Pakistan; [27]CENTOGENE GmbH, 18055 Rostock, Germany; [28]Laboratory of Human Genetics & Therapeutics, Genome Institute of Singapore, A*STAR, Singapore, Singapore; [29]Department of Neuromuscular Disease, UCL Queen Square Institute of Neurology and The National Hospital for Neurology and Neurosurgery, London,

(Affiliations continued on next page)

© 2022 The Authors. This is an open access article under the CC BY license (http://creativecommons.org/licenses/by/4.0/).

Henry Houlden,[29] Kristina Zhelcheska,[29] Melanie Pannetier,[30] Mona A. Awad,[31] Marion Lesieur-Sebellin,[32] Giulia Barcia,[32] Jeanne Amiel,[32] Julian Delanne,[1,33] Christophe Philippe,[1,13] Laurence Faivre,[1,33] Sylvie Odent,[34,35] Aida Bertoli-Avella,[27] Christel Thauvin,[1,13,36] Bekim Sadikovic,[11,12] Bruno Reversade,[28,37,38,39] Reza Maroofian,[29,39] Jérôme Govin,[3,39] Marco Tartaglia,[2,39] and Antonio Vitobello[1,13,39,*]

genes have recently been linked to ID (either syndromic or not), thus involving countless molecular pathways in its pathophysiology.[2–4] Yet, despite this incredible leap in recent knowledge, many affected individuals still remain without definite molecular diagnosis and adequate genetic counseling. This observation supports the fact that many more ID-associated genes are still to be discovered.

TMEM147 (MIM: 613585), located at 19q13.12, encodes the ubiquitously expressed 224 amino acid transmembrane protein 147, an integral membrane protein with seven transmembrane domains.[5] This protein is highly conserved in the animal kingdom with, respectively, 99% and 78% amino acid identity with mouse's and zebrafish's orthologs.[6] Very little is currently known about TMEM147's function. Previous studies using immunohistochemical analyses, co-immunoprecipitation assays, and yeast two-hybrid approach showed that TMEM147 is located in the membrane of the endoplasmic reticulum (ER) and that it regulates activity of many other proteins.[5,6] Among these are the nicalin (encoded by *NCLN* [MIM: 609156]) and NODAL modulator 2 (NOMO, encoded by *NOMO2* [MIM: 609158]), two other transmembrane proteins of the ER of which TMEM147 is a major binding partner, or cholinergic receptor muscarinic 3 (encoded by *CHRM3* [MIM: 118494]).[5,6] In particular, nicalin and NOMO have been described as antagonists of Nodal signaling during mesodermal patterning in zebrafish.[7] Nicalin is related to nicastrin, one of the γ-secretase proteins involved in Alzheimer's disease.[7,8]

A recent study identified a 360 kDa ribosome-associated complex comprising the core Sec61 (translocon) channel and the five accessory factors, transmembrane and coiled-coil domains 1 (TMCO1), coiled-coil domain-containing protein 47 (CCDC47), and the nicalin-TMEM147-NOMO complex, localized at the ribosome exit tunnel, organized around a central membrane cavity revealed by cryo-electron microscopy.[9] The translocon is a highly conserved multi-subunit protein complex that consists of three subunits (Sec61α, Sec61β, and Sec61γ) functioning as a protein-conducting channel connecting the cytoplasmic and luminal spaces on either side of the ER membrane. Within the translocon, pathogenic variants involved in a human disease have so far only been identified in *SEC61A1* (MIM: 609213), which encodes one of the Sec61α subunits of the translocon complex, generating an autosomal-dominant-inherited condition named tubulointerstitial kidney disease 5 (MIM: 617056), responsible for nephropathy with ID.[10] Among the accessory factors, recessive pathogenic variants in *TMCO1* underlie cerebrofaciothoracic dysplasia (MIM: 213980)[11–13] and recessive pathogenic variants in *CCDC47* are responsible for trichohepatoneurodevelopmental syndrome (THNS [MIM: 618268]).[14] Both conditions are associated with moderate to severe intellectual disability and dysmorphic facial features.

Besides its localization at the ER membrane, TMEM147 also localizes at the nuclear envelope, where it interacts with the C-terminal domain of the lamin B receptor (LBR), anchoring it to the inner membrane.[15] LBR is a lamin-binding protein from the inner nuclear membrane with sterol reductase activity that provides, together with lamins, essential heterochromatin docking sites at the nuclear envelope.[16,17] In mouse models, the absence of both LBR and lamin A/C leads to loss of peripheral heterochromatin and an inverted architecture with heterochromatin localizing to the nuclear interior.[18] In humans, heterozygous pathogenic variants in *LBR* (MIM: 600024) are associated with Pelger-Huët anomaly (MIM: 169400)[19] and Reynolds syndrome (MIM: 613471),[20] while bi-allelic variants are associated with Greenberg skeletal dysplasia (MIM: 215140)[21] and rhizomelic skeletal dysplasia with or without Pelger-Huët anomaly (MIM: 618019).[22,23] Pelger-Huët anomaly is characterized by abnormal nuclear shape and chromatin organization in blood granulocytes. Affected individuals show hypolobulated neutrophil nuclei with coarse chromatin. Homozygous individuals have ovoid neutrophil nuclei as well as varying degrees of developmental delay, epilepsy, and skeletal abnormalities.[21] In mouse, deletion of the LBR N-terminal domain recapitulated Pelger-Huët anomaly without disrupting X chromosome inactivation.[24]

In HeLa cells, knockdown experiments of *TMEM147* with an RNAi approach suggested a significant reduction of

UK; [30]Service d'Hématologie cellulaire et hémostase bioclinique, CHU Rennes, Rennes, France; [31]Clinical and Chemical Pathology Department, Medical Research and Clinical Studies Institute National Research Centre, Cairo, Egypt; [32]Service de Médecine Génomique des Maladies Rares, Hôpital Necker-Enfant Malades, AP-HP, Paris, France; [33]Centre de Référence maladies rares « Anomalies du Développement et syndromes malformatifs », Centre de Génétique, FHU-TRANSLAD, CHU Dijon Bourgogne, Dijon, France; [34]Service de Génétique Clinique, Centre Référence Anomalies du Développement CLAD Ouest, Univ Rennes, Rennes, France; [35]Institut de Génétique et Développement de Rennes, CNRS Inserm UMR 6290, ERL 1305, Univ Rennes, Rennes, France; [36]Centre de référence maladies rares « déficiences intellectuelles de causes rares », Centre de Génétique, FHU-TRANSLAD, CHU Dijon Bourgogne, Dijon, France; [37]Medical Genetics Department, School of Medicine, Koç University, Istanbul, Turkey; [38]Smart-Health Initiative, King Abdullah University of Science and Technology, Thuwal, Saudi Arabia
[39]These authors contributed equally
*Correspondence: quentin.thomas@chu-dijon.fr (Q.T.), antonio.vitobello@u-bourgogne.fr (A.V.)
https://doi.org/10.1016/j.ajhg.2022.08.008.

Hoechst, H3K9me3, and LBR staining accompanied by a mislocalization of LBR to the ER, indicating potential defaults in chromatin condensation.[15] By contrast, ER markers cytoskeleton-linking membrane protein CKAP4 (CLIMP-63) and RNT4 (NOGO) were upregulated.[15,25] CKAP4 is a non-glycosylated type II ER membrane protein.[26,27] Restricted localization of CKAP4 to the reticular part of the ER is mediated by self-association that retains the protein in the ER[28] and limits its mobility in the membrane.[29] The cytoplasmic segment of CKAP4 binds to microtubules, both *in vivo* and *in vitro*, and overexpression of the protein leads to a parallel rearrangement of ER and microtubules, suggesting that the protein links ER membranes to the microtubule cytoskeleton.[30] RTN4 is a member of the reticulon family of proteins and is critical for regulating the tubular structure of the ER.[31,32] Excess RTN4 limit ER luminal transport, Ca^{2+} release, and induced pluripotent stem cells (iPSCs)-derived cortical neuron's axonal extension, while RTN4 elimination reverses the effect.[33]

However, despite all previously developed elements, the involvement of *TMEM147* in human pathology remains uncertain to this day. Through an international data sharing mainly facilitated by GeneMatcher,[34] we gathered 23 individuals from 15 unrelated families with bi-allelic pathogenic *TMEM147* variants (Table S1, Figure 1A). We obtained informed consent from all affected individuals or their legal representative for the sequencing procedures and the publication of their results along with clinical and molecular data. Special consent forms were signed authorizing publication of pictures when relevant. The study was performed within the framework of the GAD ("Génétique des Anomalies du Développement") collection and approved by the appropriate institutional review board of Dijon University Hospital (DC2011-1332). These affected individuals displayed overlapping clinical features including moderate to profound ID, developmental delay, behavioral problems, and facial dysmorphism (Table 1, Figure 2A, extended clinical information is available in Table S2).

Ten individuals were female (43%) and 13 male (57%). Family history was unremarkable for all families but one (F12) in which a younger sibling died of a possibly unrelated disorder, as familial segregation analysis showed that she was not homozygous for the same *TMEM147* variant as the proband (Figure 1B). Heterozygous carriers were healthy. Twenty individuals were born to consanguineous parents (87%). Most individuals were born following uneventful pregnancies and deliveries (18/23, 78%); one pregnancy was marked by severe intrauterine growth retardation at 27 gestational weeks, facial dysmorphism evocative of Cornelia de Lange syndrome and preeclampsia (i3 – F3-IV-3), two were marked by reduced placenta flow (i6 – F5-IV-2 and 8 – F6-V-5), another one was marked by a neonatal hypoxic ischemic event (i20 – F13-II-3), and one individual delivered by C-section for fetal distress and meconial amniotic fluid (i1 – F1-II-1). She was admitted in neonatology during her first days of life with feeding and breathing difficulties. Delivery was at full term for 17 individuals (74%), at 37 weeks for four individuals (i15 – F9-II-1, i19 – F12-II-1, i20 – F13-II-3, i21 – F14-II-1), and at 35 weeks for two individuals (i3 – F3-IV-3, i5 – F4-IV-9), all from different families. Birth measurements were within normative values for most individuals; however, five presented a birth weight below −3 standard deviations, including four from the same family (i3 - F3-IV-3, i7 - F6-V-2, i8 - F6-V-5, i9 - F6-V-6, i10 - F6-V-7) and i3 (F3-IV-3), who also presented birth length and orbitofrontal circumference (OFC) below −3 standard deviations (Table S2). Data on growth parameters upon last follow-up were collected at heterogenous ages and mostly within normative values. However, eight individuals (35%) presented at least one measurement below −2 standard deviations. Amongst them, four presented isolated microcephaly that was not present at birth. Detailed measurements are presented in Table S2.

All individuals had global developmental delay and intellectual disability. Language delay was severe: upon their last follow-up, eight individuals (36%) had developed no speech at all, 12 (55%) could only babble or speak a few words, and only two (9%) could make short sentences. Of note, one individual was too young at his last examination for a reliable language evaluation (i23 – F15-V-3, age 1 year). Gross and fine motor skills were impaired for all individuals, although less severe than language delay, as most children were ambulatory by 5 years of age. Behavioral problems were observed in 15 individuals (65%) who shared a common tendency of self-injury. Other recurrent behavioral problems included hyperactivity, aggressivity, and outbursts of anger. All individuals presented ID, which was considered severe for all but one individual (either through neuropsychological testing or simple clinical evaluation).

All individuals (22/22, one missing information) displayed consistent facial dysmorphisms including coarse facies, prominent forehead, board depressed nasal root, tented mouth, long smooth philtrum, and low-set ears as major features (Table 1, Table S2, Figure 2A). We performed a computer-assisted facial visualization[35] from all available photographs in order to generate a "typical" face for a person with this *TMEM147*-associated disorder by using the Facer program (https://github.com/johnwmillr/Facer). These analyses confirmed that morphological features were marked in *TMEM147*-related disorder and highlighted some of the morphological features identified in the individuals: high forehead, long philtrum, tented mouth (Figure 2). It is noteworthy these facial particularities led attending physicians to clinically and independently suspect either an RAS-associated disorder or a chromatinopathy. Among the 11 individuals for whom a cardiac ultrasound was available, two presented a patent foramen ovale, one presented an atrial septum defect, and one a large patent ductus arteriosus. Neurological examinations were mostly unremarkable or limited to signs related to motor-development delay. Of note, three individuals (14%) presented with hypotonia, two had tonic seizures (9%), and one had synkinesis with mirror

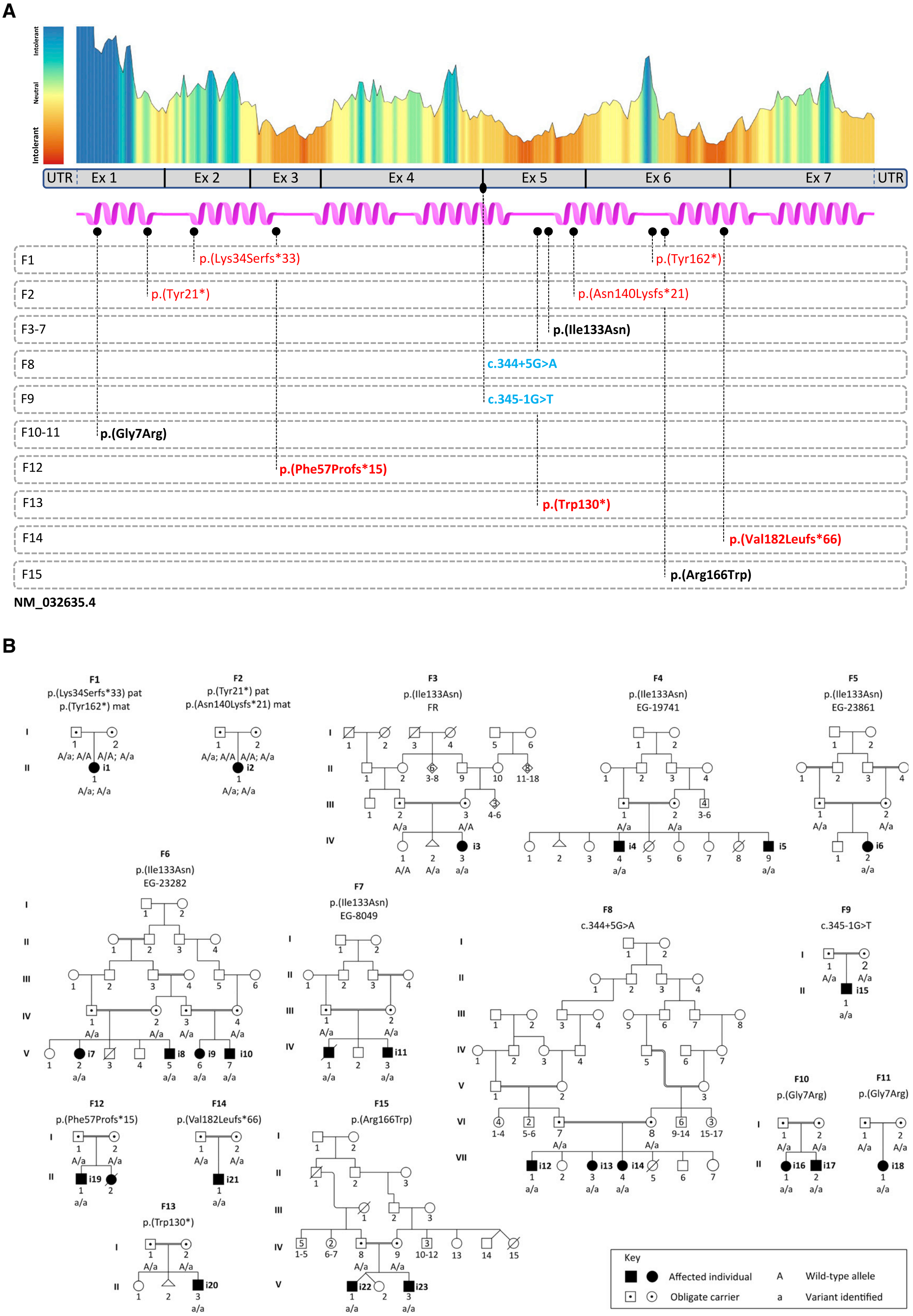

Figure 1. Individuals with *TMEM147* germline variants identified in the cohort

(A) Representation of TMEM147 (purple) with its seven helices. Metadome constraint plot and distribution of exomic regions are reported above the TMEM147 model. Families with disease-causing variants are reported below. Homozygous variants are indicated in bold. Nonsense and frameshift variants are indicated in red, splice-site variant in blue, and missense variant in black.

(B) Family pedigrees and segregation analysis of the identified variants. "A" represents the wild-type allele and "a" the mutated one.

Table 1. Clinical features of the *TMEM147* cohort

Individual family	i1 F1	i2 F2	i3 F3	i4 F4	i5 F4	i6 F5	i7 F6	i8 F6	i9 F6	i10 F6	i11 F7	i12 F8	i13 F8	i14 F8	i15 F9	i16 F10	i17 F10	i18 F11	i19 F12	i20 F13	i21 F14	i22 F15	i23 F15	Total
Sex	F	F	F	M	M	F	F	M	F	M	M	M	F	F	M	F	M	F	M	M	M	M	M	10F/ 13M
Age at last follow-up	16 y	8 y	2 y	13 y	4 y 3 m	6 y 6 m	9 y 1 m	2 y 11 m	6 y	3 y 2 m	2 y 6 m	14 y	8 y	6 y	3 y	8 y	5 y	18 y	2 y	4 y 6 m	14 m	6 y	1 y	N/A
Motor delay	+	+	+	+	+	+	+	+	+	+	+	+	+	+	+	+	+	+	+	+	+	+	+	23/23
Walking age	2 y	5 y	na	4 y	na	3 y	4 y	na	5 y	na	na	3 y	3 y	3 y	3 y	UN	UN	3 y	1.5 y	na	na	na	na	N/A
Intellectual disability	+	+	UN	+	+	+	+	+	+	+	+	+	+	+	+	+	+	+	+	+	+	+	ty	21/21
Severity	UN	se	UN	IQ: 50	IQ: 45	IQ: 53	IQ: 48	IQ: 35–40	IQ: 45	IQ: 35	IQ: 20–25	se	se	se	UN	se	se	se	mo	se	UN	IQ: 35	ty	N/A
Speech ability upon last examination	sw	ns	ba	ss	ns	sw	sw	ns	sw	ns	ns	ns	sw	ns	sw	ss	sw	sw	sw	sw	ba	ns	UN	22/22
Behavioral problems	+	+	−	+	+	+	+	+	+	+	+	−	−	−	UN	+	+	+	−	+	−	+	−	15/22
Neurological abnormalities	+	−	+	−	−	−	−	−	−	+	+	−	−	−	−	−	+	−	−	−	−	+	−	6/23
Facial dysmorphism	+	+	+	+	+	+	+	+	+	+	+	+	+	+	+	+	UN	+	+	+	+	+	+	22/22
Brain MRI abnormalities	−	−	−	+	+	+	+	+	+	+	+	UN	UN	UN	−	UN	UN	+	+	+	−	+	+	13/18

M, male; F, female; y, years; m, months; na, not acquired; UN, unknown; ty, too young; se, severe; mo, moderate; sw, single words; ns, no speech; ba, babbling; ss, short sentences; N/A, not applicable.

A

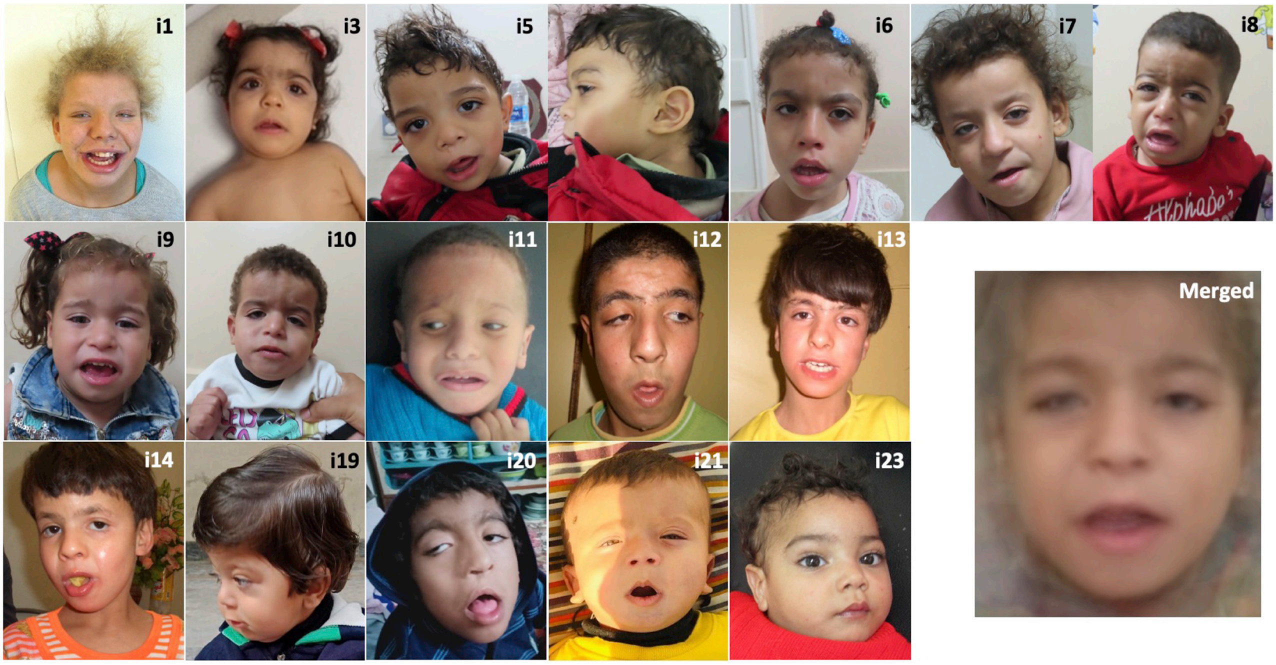

B

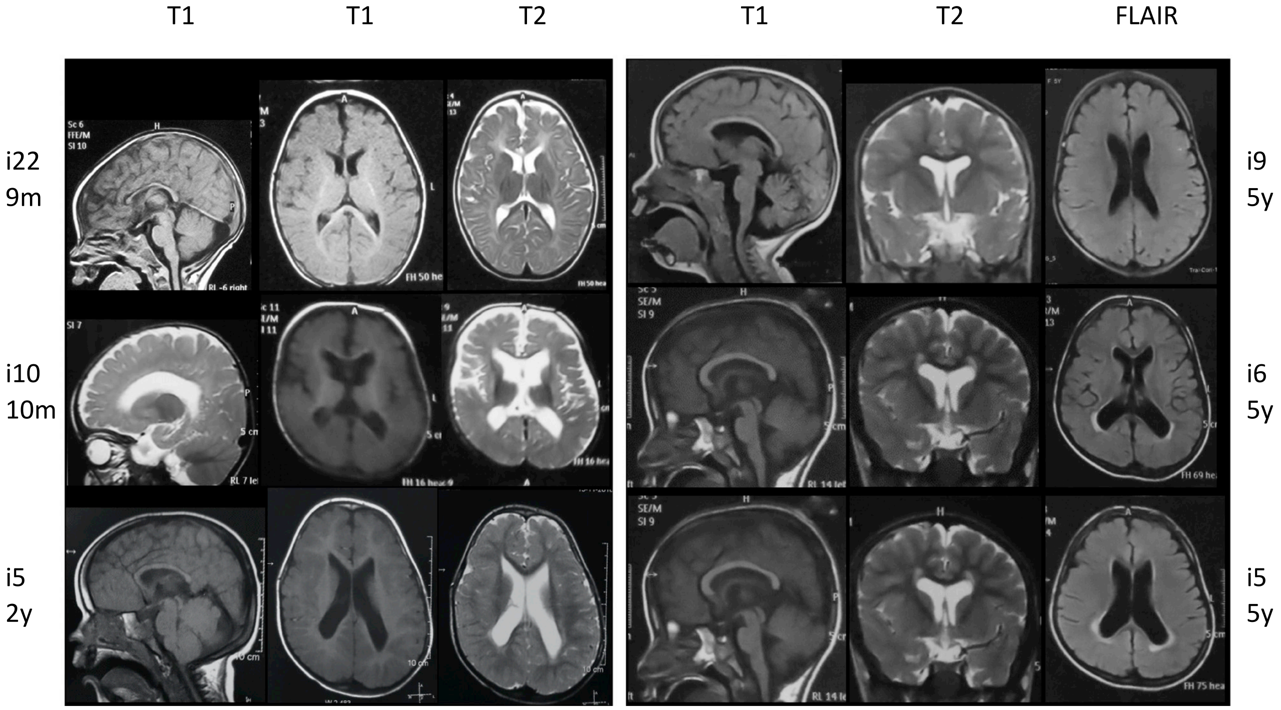

Figure 2. Photos of the affected individuals included in the cohort and brain MRI results
(A) Affected individuals showed consistent coarse facial features. A merged image was obtained by the Facer tool.
(B) Brain MRI of individuals with *TMEM147* disease-causing variants. Thin corpus callosum is present in all individuals whose brain MRIs were available. Subcortical atrophy with ventricle enlargement is observed in all individuals except i22 (F15-V-1) and i9 (F6-V-6) and was more pronounced in i10 (F6-V-7) at 10 months of age, associated with a cortical atrophy. According to age, myelination remains poor on MRI performed at 5 years of age (i5 – F4-IV-9, i6 – F5-IV-2, and i9 – F6-V-6), particularly in the temporal white matter with a hypersignal flair of the periventricular white matter in two individuals.

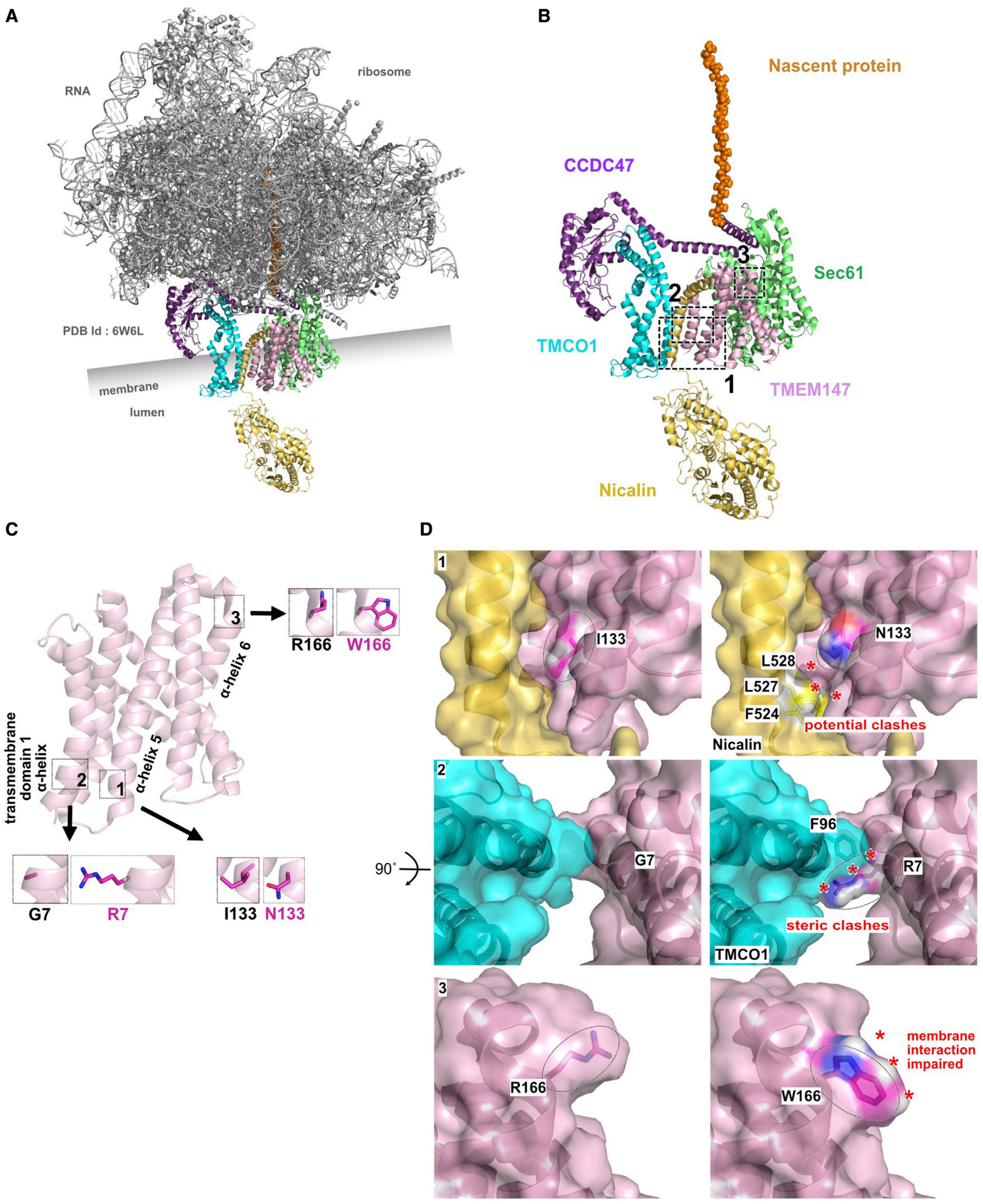

Figure 3. Structural analysis of the p.Gly7Arg, p.Ile133Asn, and p.Arg166Trp variants

(A) Cartoon representation of the ER translocon structure (PDB: 6W6L).[9] The five proteins involved in the transmembrane channel are colored: Nicalin (yellow), Sec61 (green), TMCO1 (cyan), CCDC47 (purple), and TMEM147 (pink). Ribosomal proteins and RNA are presented in gray. Membrane position is indicated with the lumen. Nascent protein sequence is shown as orange spheres inside the ribosome structure.

(B) Structural representation of TMEM147 and its main partners. For better visualization, ribosomal proteins and RNAs have been masked. Three dotted squares indicate the magnified regions used to model the variant effects. Squares 1, 2, and 3 indicate the enlarged regions used to model the p.Ile133Asn, p.Gly7Arg, and p.Arg166Trp variants, respectively.

(legend continued on next page)

movements of the hands that spontaneously resolved during childhood. Brain MRIs were available for 18 individuals and were mostly normal but revealed features including enlarged ventricles, thin corpus callosum, and white matter hyperintensities that were evocative of myelination delay alongside with mild cerebellar atrophy without dysplasia in some individuals. Cortical and subcortical atrophy was marked in only one individual (i10 – F6-V-7). Myelination delay was mostly observed in posterior regions (temporal lobes) with visible improvement upon follow-up (i5 – F4-IV-9, available MRIs at 2 and 5 years of age). Corpus callosum was thin in most individuals but never dysplastic (Figure 2B).

Twelve different variants were identified and included stop-gain (3/12), frameshift (4/12), missense (3/12), and splice-site variants (2/12) (Table S1, Figure 1). Interestingly, one missense variant (c.398T>A [p. Ile133Asn] [GenBank: NM_032635.4]) was found in five different families (F3 living in France but from North African ancestry, and F4 to F7 coming from Egypt), while a different missense variant (c.19G>C [p.Gly7Arg]) was found in two different families (F10 living in the United States and F11 coming from French and Spanish ancestries). All splice-site, nonsense, or frameshift variants but the c.540_543dup (p.Val182Leufs*66), which is 12 nucleotides before the last exon-intron junction, were predicted to result in nonsense-mediated mRNA decay (NMD).[36,37] However, in the p.Val182Leufs*66 variant, the last 45 amino acids, encoding the C-terminal domain of the protein encompassing almost one third of the 6th and the entire 7th helix, were replaced by a sequence of 66 amino acids with no significant similarity to any known human protein. Regarding the splice-site variants c.344+5G>A and c.345−1G>T, *in silico* analysis predicted them to suppress the activity of the donor splice-site of exon 4 (137 nt) and the acceptor splice-site of exon 5 (85 nt), respectively (Figures S2A–S2D). Both were predicted to alter the reading frame of the transcript (Figures S2A and S2C),[38] eventually resulting in nonsense-mediated mRNA decay. A minigene approach was established in order to measure the functional readout of the c.344+5G>A variant, confirming exon 4 skipping (Figure S2E). Most variants were present in gnomAD, but none were present at a homozygous state (Table S1). Given the prevalence of bi-allelic variants predicted to result in loss of function (LoF, i.e., nonsense, frameshift, and splice-site variants) and a congruent phenotype spectrum in the cohort, the identified missense variants were predicted to alter protein stability and function. Indeed, the three missense variants affected conserved amino acids but were predicted to variably impact protein function according to CADD,[39] PolyPhen2,[40] GERP,[41] REVEL,[42] 3Cnet,[43] and Metadome[44] algorithms (Figure 1, Table S1).

To provide functional insight into the impact of the TMEM147 variants, we performed an *in silico* structural analysis. Gly7 is located in the transmembrane domain 1, at the beginning of the α-helix 1. Ile133 is located at the beginning of α-helix 5 between the transmembrane domains 3 and 4, while Arg166 is located at the beginning of α-helix 6 where the transmembrane region initiates (Figure S1). The 3D structure of the translocon was recently resolved by cryo-electron microscopy (PDB: 6W6L, Figure 3A)[9] and used here to further explore the possible functional impact of the three missense changes (Figures 3B and 3C). Considering the p.Gly7Arg substitution, the arginine extension was predicted to push into the TMCO1-facing amino terminal region, creating a steric conflict with the Phe96 (Figure 3, insert 2, asterisks). Similarly, p.Ile133Asn generated tensions with the adjacent amino acids in TMEM147 (data not shown) and its extension towards nicalin, would cause potential clashes with three amino acids in nicalin: Phe524, Leu527, and Leu528 (Figure 3, insert 1, asterisks). The c.496C>T (p.Arg166Trp) variant changed the surface environment and net charge. Even though this side of the chain is not in direct contact with other proteins of the ER translocon, it is located in the vicinity of the membrane itself and the transmembrane part of the α-helix 6. Such drastic changes in both conformation and charges could explain the instability observed for this variant (Figure 3, insert 3).

To validate the impact of the identified missense variants on TMEM147 stability, we generated three V5-tagged TMEM147 mutants and assessed the protein level of each in transiently transfected COS-1 cells, basally and after 1 h of treatment with the protein synthesis inhibitor cycloheximide (CHX). Immunoblotting and relative quantitative analyses revealed a variably reduced level of the three TMEM147 mutants, indicating that the tested variants significantly impact TMEM147 stability; p.Gly7Arg was the most unstable and p.Arg166Trp was the least (Figure 4A). Treatment with bafilomycin, a late-stage inhibitor of

(C) Cartoon representation of TMEM147 presenting the three enlarged regions. In the larger dotted inserts, the wild-type situation for each amino acid is displayed as stick. 1, Ile133; 2, Gly7; and 3, Arg166. In the adjacent insert, the mutated amino acid is modeled as a comparison. Only the side chains are represented in purple, leaving the α-carbon as a pink ribbon representation. Hydrogens have been masked for better visualization.
(D) (1, left) Surface rendering of the Ile133 area. The α-helix faces the carboxy-terminal region of Nicalin. (1, right) Surface rendering of the Ile133Asn variant as a model. From the 11 possible rotamers for asparagine in this context, the one causing the minimum of constraints was selected. (2, left) Surface rendering of the Gly7 area. The molecule was rotated by 90° and viewed from above. (2, right) Surface rendering of the Gly7Arg variant as a model. From the 24 possible rotamers for arginine in this context, all of them are causing mild to severe steric clashes with TMEM147 itself or the surrounding polypeptidic chains. The rotamer causing the lowest degree of perturbation was arbitrarily selected. (3, left) Surface rendering of the Arg166 area. (2, right) Surface rendering of the p.Arg166Trp variant as a model. From the seven possible rotamers for tryptophan in this context, all of them are causing mild steric clashes with TMEM147. The rotamer causing the lowest degree of perturbation was arbitrarily selected. Dotted shapes indicate the positions of the amino acids in the chain.

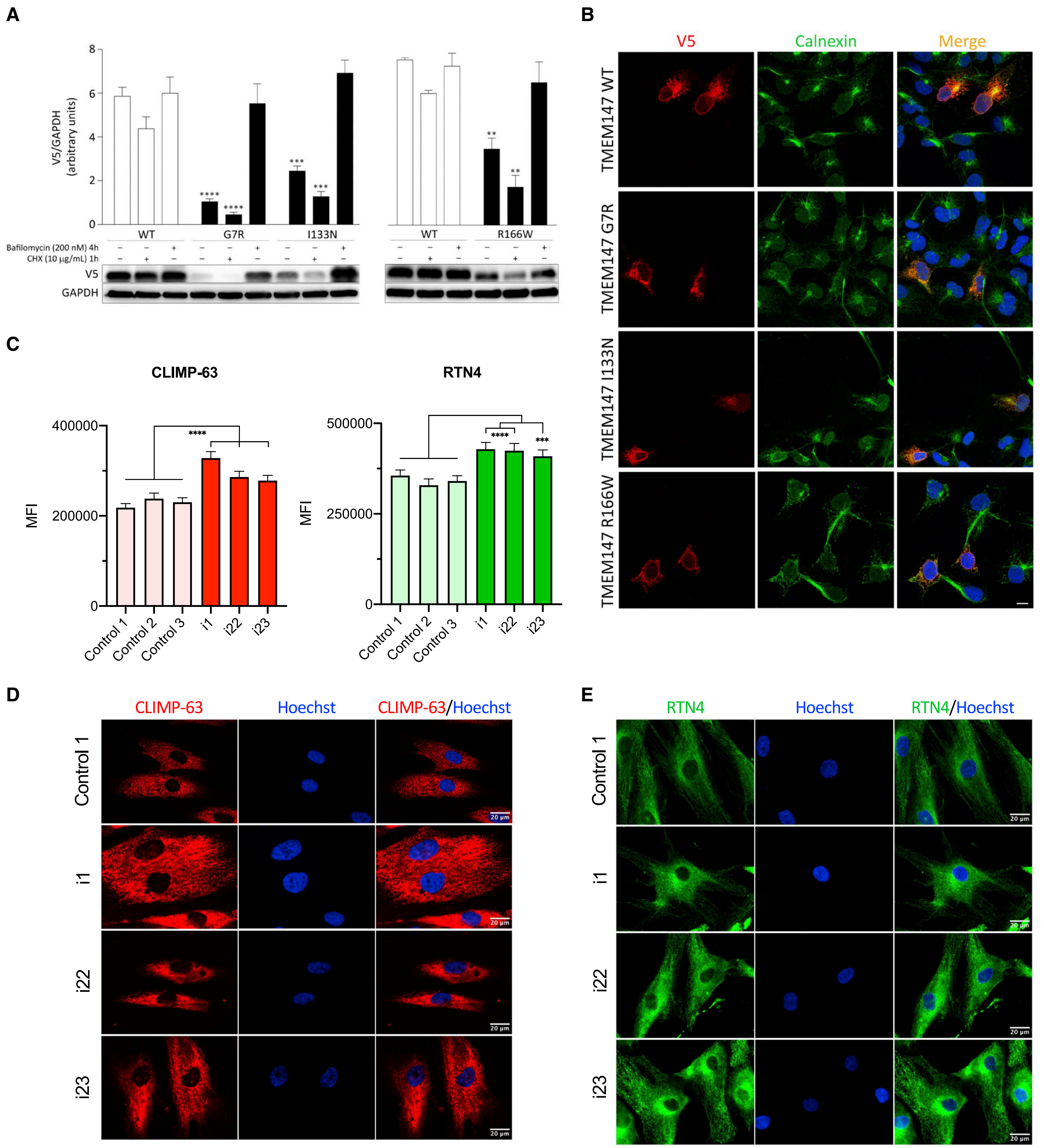

Figure 4. Biochemical characterization of the TMEM147 mutant proteins and immunostainings in fibroblasts
(A) Accelerated degradation of the TMEM147^{Arg7Gly} (R7G), TMEM147^{Ile133Asn} (I133N), and TMEM147^{Arg166Trp} (R166W) proteins. Immunoblot analysis shows WT and variant V5-tagged TMEM147 protein levels in transfected COS-1 cells, basally and after CHX (10 μg/mL) or bafilomycin (200 nM) treatment. GAPDH was used as loading control. Representative blots (below) and mean ± SD densitometry values (above) of three independent experiments are shown. Asterisks indicate statistically significant differences compared with WT TMEM147 (****$p \leq 0.0001$; ***$p \leq 0.001$; **$p \leq 0.05$; two-way ANOVA followed by Tukey's multiple comparison test).
(B) Subcellular localization of transiently expressed V5-tagged WT or mutant TMEM147 proteins in COS-1 cells under steady-state conditions revealed by confocal microscopy analysis. Cells were stained with the anti-V5 monoclonal antibody (red). Co-localization analysis was performed using the endoplasmic reticulum marker calnexin (green). Merged images with nuclei (Hoechst 33342 staining, blue) are displayed on the right. Scale bar, 10 μm.
(C) Quantification of mean fluorescence signals ± SEM detected in (D). Three technical replicates were performed per cell line. A total of 150 measurements per cell line were performed. Asterisks indicate statistically significant differences compared with cell lines from

(legend continued on next page)

autophagy and lysosomal protein degradation, rescued the reduced levels of all mutants (Figure 4A), indicating that accelerated degradation of TMEM147 mutant proteins occurs via the autophagy-lysosomal pathway. We then imaged COS-1 cells transiently transfected with plasmids encoding V5-tagged TMEM147, and we confirmed the localization of the wild-type (WT) protein to the endoplasmic reticulum, where it colocalizes with calnexin (Figure 4B). Assessment of the subcellular localization of the TMEM147 mutants showed that, similarly to the WT protein, all tested proteins colocalized with calnexin, indicating proper targeting to the endoplasmic reticulum (Figure 4B).

Next, we sought to investigate the functional readout of TMEM147 bi-allelic loss of function in derived primary cultures. For this purpose, we obtained primary fibroblast cells from i1 (F1-II-1) and i22 and i23 (F15-V-1 and F15-V-3, respectively) with compound heterozygous nonsense c.486C>G (p.Tyr162*) and frameshift c.100_118del (p.Lys34Serfs*33) variants or the homozygous p.Arg166Trp missense variant in *TMEM147*, respectively. ER markers CKAP4 (CLIMP-63) and RTN4 were significantly upregulated in TMEM147 individuals as compared to healthy volunteers (Figures 4C–4E). This upregulation was accompanied by a concomitant parallel rearrangement of ER, indicating that in absence of TMEM147, morphological and physiological changes take place (Figure 4D and 4E). *TMEM147*-deficient cells were previously shown to alter LBR localization. We thus performed LBR localization analysis in fibroblasts from healthy controls and affected individuals and observed that it was restricted to the nuclear envelope in the former, while it was evenly distributed throughout the nucleus in the latter condition (Figure 5A). Interestingly, nuclei from affected individuals were associated with a higher risk of showing abnormal segmentation as compared to cultures from healthy individuals, indicating that LBR mislocalization could cause nuclear instability (Figures 5D and 5C).

Finally, we hypothesized that hematological abnormalities such as Pelger-Huët anomaly would likely be present in *TMEM147*-null individuals. Cytological examination of the blood smear frequently revealed lobulation defects of the neutrophils (Figures 5D and S3A), with forms of hyposegmentation similar to the Pelger-Huët anomaly, although with a lower proportion, bilobed aspects in the shape of a sack with two round or oval lobes connected by a thread of chromatin, rare chromatin clumping, and frequent nuclear appendices (caryoschizes or drumsticks), isolated or associated with the preceding abnormalities. These cytological abnormalities concerned about 15%–25% of neutrophils and were also identified in sparse eosinophils, indicating that the anomaly affects all granulocytes but is most evident in polymorphonuclear neutrophils (Figure S3B). None were observed in platelets, red blood cells, lymphocytes, and monocytes. Formally, these hematological features could not be classified as a typical Pelger-Huët anomaly, but rather pseudo-Pelger-Huët anomaly because the majority of mature granulocytes were not bilobed, although they clearly belonged to the same phenotypic spectrum.

TMEM147 is widely expressed in the developing human brain (GTEx: https://gtexportal.org/home/). To further explore the relevance of *TMEM147* function during neurodevelopment, we studied its correlation with an established set of neurodevelopmental disorder (NDD)-associated genes.[45] By using the ARCHS4 database (ARCHS4: https://maayanlab.cloud/archs4/),[46] we observed that *TMEM147* expression is significantly correlated to this group of genes in the brain (Figure S4A, p value < 0.01) and that this correlation of expression patterns is brain specific (Figure S4B). To further support this tissue-specific correlation, NDD genes with correlation values greater than $|0.3|$ with *TMEM147* were also analyzed by means of GTEx database. Consistently, the cluster analysis highlighted that the co-expression profile of *TMEM147* and its most correlated NDD-associated genes clustered in two main groups, one of which specifically containing all brain sites, indicating a tissue-specific correlation among these genes (Figure S4C). Although several individuals in our cohort presented white matter abnormalities such as white matter atrophy, thin corpus callosum, dilated ventricles, enlarged cortical sulci, and possible myelination delay, it is noteworthy that *TMEM147*-related disorders cannot be considered as primary white matter disorders but rather as encephalopathies. Indeed, the lack of upper-motor neuron syndrome in all individuals, the presence of global hypotonia in several individuals, and the overall lack of clear white matter hypomyelination or demyelination in the available brain MRI is congruent with an encephalopathy rather than a leukodystrophy. Interestingly, among the list of NDD genes with strong co-expression with *TMEM147*, nine genes correlated with the HPO term "encephalopathy" (HP: 0001298), i.e., *CDKL5* (developmental and epileptic encephalopathy [MIM: 300672]), *CLTC* (intellectual developmental disorder, autosomal dominant 56 [MIM: 617854]), *KMT2E* (O'Donnel-Luria-Rodan syndrome [MIM: 618512]), *NRXN1* (Pitt-Hopkins-like syndrome 2 [MIM: 614325]), *SCN1A* (developmental and epileptic encephalopathy 6B, non-Dravet [MIM: 619317]; Dravet syndrome [MIM: 607208]; febrile seizures, familial, 3A [MIM: 604403]), *SCN2A* (developmental and epileptic encephalopathy 11 [MIM: 613721]), *TCF4* (Pitt-Hopkins syndrome [MIM: 610954]), *UNC80* (hypotonia, infantile, with psychomotor retardation and characteristic facies 2 [MIM: 616801]), and *WDR45* (neurodegeneration with brain iron accumulation 5 [MIM: 300894]).

healthy individuals: control 1, control 2, and control 3 (****p $\leq$ 0.0001; ***p $\leq$ 0.001; **p $\leq$ 0.01; *p $\leq$ 0.05; one-sample Wilcoxon test, based on the average of the three control samples).
(D and E) Localization analysis of CKAP4 (CLIMP-63) (D) and RTN4 (E) in fibroblasts from i1, i22, and i23 and healthy control individuals (only control 1 is shown in the figure). Scale bar, 20 μm.

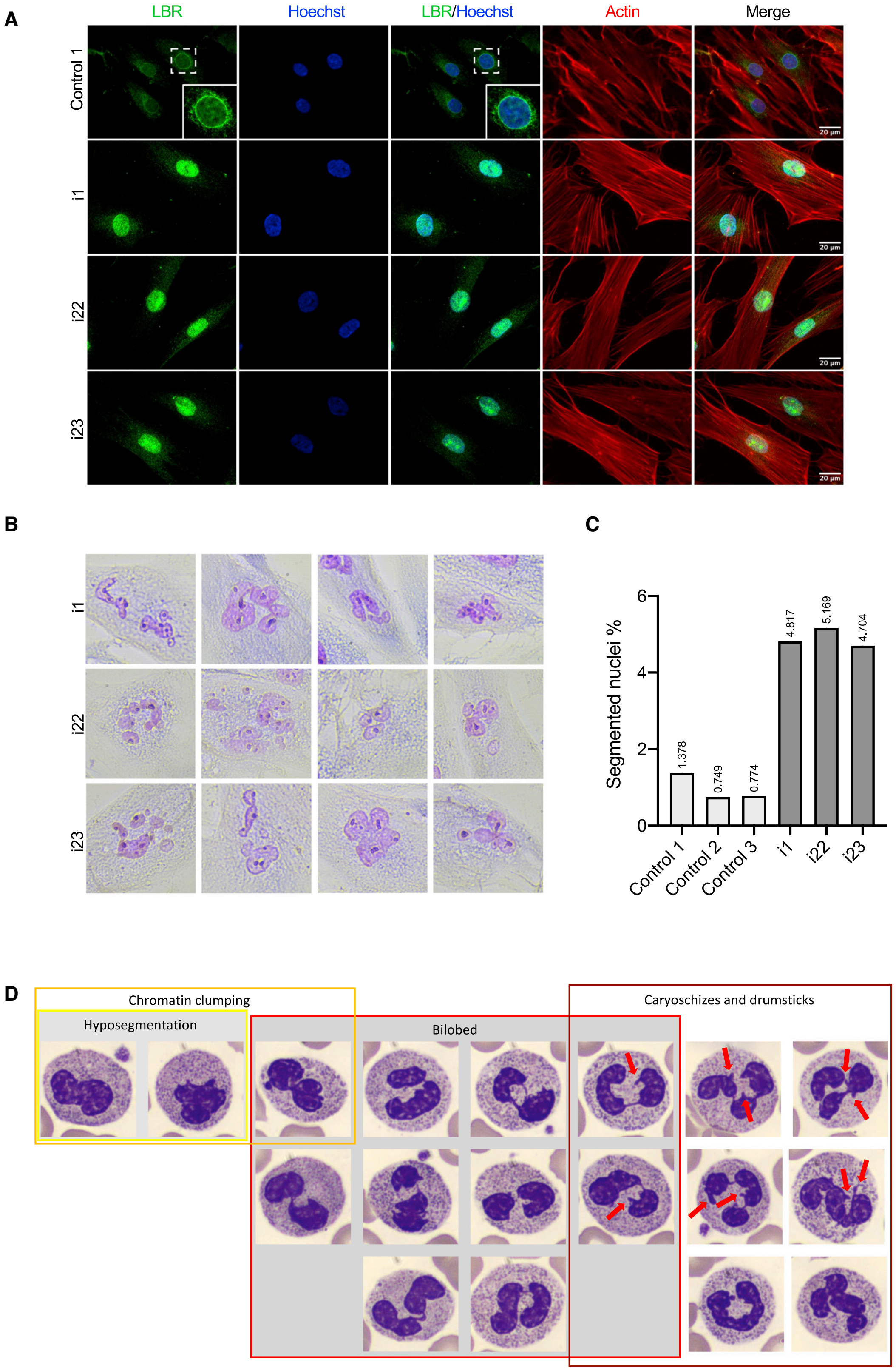

Figure 5. LBR localization, nuclear morphology in fibroblasts, and Pelger-Huët-like anomaly in granulocytes
(A) Localization analysis of LBR and actin in fibroblasts from i1, i22, and i23 and healthy control individuals (only control 1 is shown in the figure). Scale bar, 20 μm. The inset in control 1 line shows a higher magnification of the nucleus indicated by the dashed square. Actin staining shows that TMEM147 does not affect gross cell morphology.

(legend continued on next page)

The individuals in our cohort presented global developmental delay with particularly severe speech delay (only one individual could make short sentences upon last follow-up) and psychomotor delay. Even though all individuals had delayed motor milestones, they all eventually acquired walking, although gait was often described as imperfect or unsteady. Clinical follow-up revealed that fine motor skills were also impaired. Severe intellectual disability was observed in all individuals. Beyond these developmental features, the individuals' phenotype was mostly made of marked facial dysmorphism. Of note, the facial features of a large proportion of these individuals were suggestive of a RASopathy or a chromatinopathy.[47–50] For some, sequencing of a panel of genes associated with these diseases was performed. However, Episign analysis revealed a genome-wide DNA methylation profile incompatible with all 59 episignatures assessed, including chomatinopathy conditions such as Cornelia de Lange, Rubinstein-Taybi, Coffin-Siris, and Wiedemann-Steiner syndromes. A typical positive case presents with a methylation variant pathogenicity (MVP) greater than 0.5 and both tested individuals presented with MVPs of 0.02 or less (Figure S4D).

TMEM147 was originally identified as a protein complexing with NOMO and nicalin.[5] On the basis of the documented codependence among the three proteins, dysregulation of *TMEM147* is expected to alter the nicalin-NOMO protein complex and, consequently, the various molecular pathways it is involved in. Nicalin-NOMO interaction has been shown to modulate (antagonize) the Nodal pathway.[7] For instance, previous efforts had shown that the Nodal pathway is paramount in early embryonic development where it takes part in mesoendoderm induction,[51] right-left patterning,[52,53] and neural patterning as Nodal antagonism is required for the formation of neuroectoderm.[51] Some actors of the TGF-β superfamily even take part in the final patterning of the vertebrate central nervous system.[54] Overall, these elements made *TMEM147* an even stronger candidate for neurodevelopmental disorders.

Here, we described a cohort of 23 affected individuals identified through an international collaboration who harbored bi-allelic disease-causing variants in *TMEM147*, thus offering a clinical profiling of a neurodevelopmental disorder caused by *TMEM147* loss of function. Splice-site, nonsense, or frameshift disease-causing variants were largely represented in our cohort. Missense variants, resulting in accelerated degradation of the mutated protein via the autophagy-lysosomal pathway, were identified in several unrelated families showing consistent clinical features. No phenotypic differences between affected individuals with predicted loss-of-function versus missense variants were noted. The analysis of primary fibroblasts and granulocytes provided functional evidence of endoplasmic reticulum and the nuclear envelope dysfunction. Interestingly, previous experiments had shown that *TMEM147* knockdown results in a drastic decline of LBR protein levels, an altered diffusional mobility of LBR, and its relocation to the ER in HeLa cells.[15] However, our experiments demonstrate that the diffusional mobility of LBR is not accompanied by decreased protein levels in primary fibroblast cells from affected individuals. *TMEM147* is ubiquitously expressed, however co-expression data suggested a strong correlation with neurodevelopmental genes linking subcellular dysfunction to key genes involved in brain development.

Overall, *TMEM147* should be considered as a gene responsible for intellectual disability, developmental delay, and facial dysmorphism and should be considered as a potential differential diagnosis with chromatinopathies or RASopathies.

Data and code availability

All of the data produced in this study are included in the main manuscript and in supplemental material.

Supplemental information

Supplemental information can be found online at https://doi.org/10.1016/j.ajhg.2022.08.008.

Acknowledgments

We thank the families and affected individuals for taking part in the study. We thank the University of Burgundy Centre de Calcul (CCuB) for technical support and management of the informatics platform. We thank the "Centre de Ressources Biologiques Ferdinand Cabanne" (CHU Dijon) for sample biobanking. This work was supported by grants from Dijon University Hospital, the ISITE-BFC (PIA ANR), and the European Union through the FEDER programs, EJP-RD (NSEuroNET), AIRC (IG21614), and Italian Ministry of Health (5x1000). Sequencing for individual 15 was funded by the Institute for Information and Communications Technology Promotion (IITP) grant from the Korean government (MSIT) (2018-0-00861, Intelligent SW Technology Development for Medical Data Analysis). This study makes use of DECIPHER (http://decipher.sanger.ac.uk), which is funded by the Wellcome (www.ddduk.org/access.html).[55] This research was made possible through access to the data and findings generated by the 100KGP. The 100KGP is managed by Genomics England Limited (a wholly owned company of the Department of Health and Social Care). The 100KGP is funded by the National Institute for Health Research and NHS England. The Wellcome Trust, Cancer Research UK, and the Medical Research Council have also funded research infrastructure. The 100KGP uses data provided by individuals and collected by the National Health Service as part of their care and support. Several authors of this publication are members of the European Reference Network for Developmental Anomalies and Intellectual Disability (ERN-ITHACA). The Solve-RD project has received

(B) Nuclear segmentation is observed in fibroblast cell nuclei from affected individuals. Fibroblasts were stained with the May Grunwald-Giemsa (MGG) method.
(C) Quantification of the nuclear segmentation observed in fibroblast cell lines.
(D) Chromatin clumping, hyposegmentation, bilobed nuclei, carioschizes, and drumsticks (red arrows) observed in neutrophils of i1.

funding from the European Union's Horizon 2020 research and innovation program under grant agreement no. 779257.

Declaration of interests

S.A., I.K., J.P.B., and A.B.-A. are employees of Centogene GmbH.

Received: June 11, 2022
Accepted: August 9, 2022
Published: August 30, 2022

References

1. Schalock, R.L., Luckasson, R., and Tassé, M.J. (2021). An Overview of intellectual disability: Definition, diagnosis, classification, and systems of supports (12th ed.). Am. J. Intellect. Dev. Disabil. *126*, 439–442.
2. Ki, C.-S. (2021). Recent Advances in the Clinical Application of Next-Generation Sequencing. Pediatr. Gastroenterol. Hepatol. Nutr. *24*, 1–6.
3. Alazami, A.M., Patel, N., Shamseldin, H.E., Anazi, S., Al-Dosari, M.S., Alzahrani, F., Hijazi, H., Alshammari, M., Aldahmesh, M.A., Salih, M.A., et al. (2015). Accelerating Novel Candidate Gene Discovery in Neurogenetic Disorders via Whole-Exome Sequencing of Prescreened Multiplex Consanguineous Families. Cell Rep. *10*, 148–161.
4. Anazi, S., Maddirevula, S., Salpietro, V., Asi, Y.T., Alsahli, S., Alhashem, A., Shamseldin, H.E., AlZahrani, F., Patel, N., Ibrahim, N., et al. (2017). Expanding the genetic heterogeneity of intellectual disability. Hum. Genet. *136*, 1419–1429.
5. Dettmer, U., Kuhn, P.-H., Abou-Ajram, C., Lichtenthaler, S.F., Krüger, M., Kremmer, E., Haass, C., and Haffner, C. (2010). Transmembrane Protein 147 (TMEM147) Is a Novel Component of the Nicalin-NOMO Protein Complex. J. Biol. Chem. *285*, 26174–26181.
6. Rosemond, E., Rossi, M., McMillin, S.M., Scarselli, M., Donaldson, J.G., and Wess, J. (2011). Regulation of M_3 Muscarinic Receptor Expression and Function by Transmembrane Protein 147. Mol. Pharmacol. *79*, 251–261.
7. Haffner, C., Frauli, M., Topp, S., Irmler, M., Hofmann, K., Regula, J.T., Bally-Cuif, L., and Haass, C. (2004). Nicalin and its binding partner Nomo are novel Nodal signaling antagonists. EMBO J. *23*, 3041–3050.
8. Haffner, C., Dettmer, U., Weiler, T., and Haass, C. (2007). The Nicastrin-like Protein Nicalin Regulates Assembly and Stability of the Nicalin-Nodal Modulator (NOMO) Membrane Protein Complex. J. Biol. Chem. *282*, 10632–10638.
9. McGilvray, P.T., Anghel, S.A., Sundaram, A., Zhong, F., Trnka, M.J., Fuller, J.R., Hu, H., Burlingame, A.L., and Keenan, R.J. (2020). An ER translocon for multi-pass membrane protein biogenesis. Elife *9*, e56889.
10. Bolar, N.A., Golzio, C., Živná, M., Hayot, G., Van Hemelrijk, C., Schepers, D., Vandeweyer, G., Hoischen, A., Huyghe, J.R., Raes, A., et al. (2016). Heterozygous Loss-of-Function SEC61A1 Mutations Cause Autosomal-Dominant Tubulo-Interstitial and Glomerulocystic Kidney Disease with Anemia. Am. J. Hum. Genet. *99*, 174–187.
11. Xin, B., Puffenberger, E.G., Turben, S., Tan, H., Zhou, A., and Wang, H. (2010). Homozygous frameshift mutation in *TMCO1* causes a syndrome with craniofacial dysmorphism, skeletal anomalies, and mental retardation. Proc. Natl. Acad. Sci. USA *107*, 258–263.
12. Caglayan, A.O., Per, H., Akgumus, G., Gumus, H., Baranoski, J., Canpolat, M., Calik, M., Yikilmaz, A., Bilguvar, K., Kumandas, S., and Gunel, M. (2013). Whole-exome sequencing identified a patient with TMCO1 defect syndrome and expands the phenotic spectrum: Letter to the Editor. Clin. Genet. *84*, 394–395.
13. Sharkia, R., Zalan, A., Jabareen-Masri, A., Hengel, H., Schöls, L., Kessel, A., Azem, A., and Mahajnah, M. (2019). A novel biallelic loss-of-function mutation in *TMCO1* gene confirming and expanding the phenotype spectrum of cerebro-facio-thoracic dysplasia. Am. J. Med. Genet. *61168*.
14. Morimoto, M., Waller-Evans, H., Ammous, Z., Song, X., Strauss, K.A., Pehlivan, D., Gonzaga-Jauregui, C., Puffenberger, E.G., Holst, C.R., Karaca, E., et al. (2018). Bi-allelic CCDC47 Variants Cause a Disorder Characterized by Woolly Hair, Liver Dysfunction, Dysmorphic Features, and Global Developmental Delay. Am. J. Hum. Genet. *103*, 794–807.
15. Christodoulou, A., Maimaris, G., Makrigiorgi, A., Charidemou, E., Lüchtenborg, C., Ververis, A., Georgiou, R., Lederer, C.W., Haffner, C., Brügger, B., and Santama, N. (2020). TMEM147 interacts with Lamin B Receptor, regulates its localization and levels, and affects cholesterol homeostasis. J. Cell Sci. *133*, 245357.
16. Pyrpasopoulou, A., Meier, J., Maison, C., Simos, G., and Georgatos, S.D. (1996). The lamin B receptor (LBR) provides essential chromatin docking sites at the nuclear envelope. EMBO J. *15*, 7108–7119.
17. Duband-Goulet, I., Courvalin, J.C., and Buendia, B. (1998). LBR, a chromatin and lamin binding protein from the inner nuclear membrane, is proteolyzed at late stages of apoptosis. J. Cell Sci. *111*, 1441–1451.
18. Solovei, I., Wang, A.S., Thanisch, K., Schmidt, C.S., Krebs, S., Zwerger, M., Cohen, T.V., Devys, D., Foisner, R., Peichl, L., et al. (2013). LBR and Lamin A/C Sequentially Tether Peripheral Heterochromatin and Inversely Regulate Differentiation. Cell *152*, 584–598.
19. Hoffmann, K., Dreger, C.K., Olins, A.L., Olins, D.E., Shultz, L.D., Lucke, B., Karl, H., Kaps, R., Müller, D., Vayá, A., et al. (2002). Mutations in the gene encoding the lamin B receptor produce an altered nuclear morphology in granulocytes (Pelger–Huët anomaly). Nat. Genet. *31*, 410–414.
20. Reynolds, T.B., Denison, E.K., Frankl, H.D., Lieberman, F.L., and Peters, R.L. (1971). Primary biliary cirrhosis with scleroderma, Raynaud's phenomenon and telangiectasia. Am. J. Med. *50*, 302–312.
21. Greenberg, C.R., Rimoin, D.L., Gruber, H.E., DeSa, D.J., Reed, M., Lachman, R.S., Optiz, J.M., and Reynolds, J.F. (1988). A new autosomal recessive lethal chondrodystrophy with congenital hydrops. Am. J. Med. Genet. *29*, 623–632.
22. Borovik, L., Modaff, P., Waterham, H.R., Krentz, A.D., and Pauli, R.M. (2013). Pelger-huet anomaly and a mild skeletal phenotype secondary to mutations in *LBR*. Am. J. Med. Genet. *161*, 2066–2073.
23. Sobreira, N., Modaff, P., Steel, G., You, J., Nanda, S., Hoover-Fong, J., Valle, D., and Pauli, R.M. (2015). An anadysplasia-like, spontaneously remitting spondylometaphyseal dysplasia secondary to lamin B receptor (*LBR*) gene mutations: Further definition of the phenotypic heterogeneity of *LBR* -bone dysplasias. Am. J. Med. Genet. *167*, 159–163.
24. Young, A.N., Perlas, E., Ruiz-Blanes, N., Hierholzer, A., Pomella, N., Martin-Martin, B., Liverziani, A., Jachowicz, J.W., Giannakouros, T., and Cerase, A. (2021). Deletion of LBR N-terminal domains recapitulates Pelger-Huet anomaly

phenotypes in mouse without disrupting X chromosome inactivation. Commun. Biol. *4*, 478.
25. Maimaris, G., Christodoulou, A., Santama, N., and Lederer, C.W. (2021). Regulation of ER Composition and Extent, and Putative Action in Protein Networks by ER/NE Protein TMEM147. Int. J. Mol. Sci. *22*, 10231.
26. Vedrenne, C., Klopfenstein, D.R., and Hauri, H.-P. (2005). Phosphorylation Controls CLIMP-63–mediated Anchoring of the Endoplasmic Reticulum to Microtubules. Mol. Biol. Cell *16*, 1928–1937.
27. Schweizer, A., Ericsson, M., Bächi, T., Griffiths, G., and Hauri, H.P. (1993). Characterization of a novel 63 kDa membrane protein. Implications for the organization of the ER-to-Golgi pathway. J. Cell Sci. *104*, 671–683.
28. Schweizer, A., Rohrer, J., Hauri, H.P., and Kornfeld, S. (1994). Retention of p63 in an ER-Golgi intermediate compartment depends on the presence of all three of its domains and on its ability to form oligomers. J. Cell Biol. *126*, 25–39.
29. Klopfenstein, D.R., Klumperman, J., Lustig, A., Kammerer, R.A., Oorschot, V., and Hauri, H.-P. (2001). Subdomain-Specific Localization of Climp-63 (P63) in the Endoplasmic Reticulum Is Mediated by Its Luminal α-Helical Segment. J. Cell Biol. *153*, 1287–1300.
30. Klopfenstein, D.R., Kappeler, F., and Hauri, H.-P. (1998). A novel direct interaction of endoplasmic reticulum with microtubules. EMBO J. *17*, 6168–6177.
31. Sutendra, G., Dromparis, P., Wright, P., Bonnet, S., Haromy, A., Hao, Z., McMurtry, M.S., Michalak, M., Vance, J.E., Sessa, W.C., and Michelakis, E.D. (2011). The Role of Nogo and the Mitochondria–Endoplasmic Reticulum Unit in Pulmonary Hypertension. Sci. Transl. Med. *3*, 88ra55.
32. Voeltz, G.K., Prinz, W.A., Shibata, Y., Rist, J.M., and Rapoport, T.A. (2006). A Class of Membrane Proteins Shaping the Tubular Endoplasmic Reticulum. Cell *124*, 573–586.
33. Konno, T., Parutto, P., Bailey, D.M.D., Davì, V., Crapart, C., Awadelkareem, M.A., Hockings, C., Brown, A., Xiang, K.M., Agrawal, A., et al. (2021). Endoplasmic Reticulum morphological regulation by RTN4/NOGO modulates neuronal regeneration by curbing luminal transport. Cell Biol.
34. Sobreira, N., Schiettecatte, F., Valle, D., and Hamosh, A. (2015). GeneMatcher: A Matching Tool for Connecting Investigators with an Interest in the Same Gene. Hum. Mutat. *36*, 928–930.
35. Ferry, Q., Steinberg, J., Webber, C., FitzPatrick, D.R., Ponting, C.P., Zisserman, A., and Nellåker, C. (2014). Diagnostically relevant facial gestalt information from ordinary photos. Elife *3*, e02020.
36. Nagy, E., and Maquat, L.E. (1998). A rule for termination-codon position within intron-containing genes: when nonsense affects RNA abundance. Trends Biochem. Sci. *23*, 198–199.
37. Lindeboom, R.G.H., Vermeulen, M., Lehner, B., and Supek, F. (2019). The impact of nonsense-mediated mRNA decay on genetic disease, gene editing and cancer immunotherapy. Nat. Genet. *51*, 1645–1651.
38. Jaganathan, K., Kyriazopoulou Panagiotopoulou, S., McRae, J.F., Darbandi, S.F., Knowles, D., Li, Y.I., Kosmicki, J.A., Arbelaez, J., Cui, W., Schwartz, G.B., et al. (2019). Predicting Splicing from Primary Sequence with Deep Learning. Cell *176*, 535–548.e24.
39. Rentzsch, P., Witten, D., Cooper, G.M., Shendure, J., and Kircher, M. (2019). CADD: predicting the deleteriousness of variants throughout the human genome. Nucleic Acids Res. *47*, D886–D894.
40. Adzhubei, I., Jordan, D.M., and Sunyaev, S.R. (2013). Predicting Functional Effect of Human Missense Mutations Using PolyPhen-2. . Current Protocols in Human Genetics, *76*.
41. Cooper, G.M., Stone, E.A., Asimenos, G., NISC Comparative Sequencing Program, Batzoglou, S., and Sidow, A. (2005). Distribution and intensity of constraint in mammalian genomic sequence. Genome Res. *15*, 901–913.
42. Ioannidis, N.M., Rothstein, J.H., Pejaver, V., Middha, S., McDonnell, S.K., Baheti, S., Musolf, A., Li, Q., Holzinger, E., Karyadi, D., et al. (2016). REVEL: An Ensemble Method for Predicting the Pathogenicity of Rare Missense Variants. Am. J. Hum. Genet. *99*, 877–885.
43. Won, D.-G., Kim, D.-W., Woo, J., and Lee, K. (2021). 3Cnet: pathogenicity prediction of human variants using multitask learning with evolutionary constraints. Bioinformatics *37*, 4626–4634.
44. Wiel, L., Baakman, C., Gilissen, D., Veltman, J.A., Vriend, G., and Gilissen, C. (2019). MetaDome: Pathogenicity analysis of genetic variants through aggregation of homologous human protein domains. Hum. Mutat. *40*, 1030–1038.
45. Radio, F.C., Pang, K., Ciolfi, A., Levy, M.A., Hernández-García, A., Pedace, L., Pantaleoni, F., Liu, Z., de Boer, E., Jackson, A., et al. (2021). SPEN haploinsufficiency causes a neurodevelopmental disorder overlapping proximal 1p36 deletion syndrome with an episignature of X chromosomes in females. Am. J. Hum. Genet. *108*, 502–516.
46. Lachmann, A., Torre, D., Keenan, A.B., Jagodnik, K.M., Lee, H.J., Wang, L., Silverstein, M.C., and Ma'ayan, A. (2018). Massive mining of publicly available RNA-seq data from human and mouse. Nat. Commun. *9*, 1366.
47. Rauen, K.A. (2013). The RASopathies. Annu. Rev. Genomics Hum. Genet. *14*, 355–369.
48. Asadollahi, R., Oneda, B., Sheth, F., Azzarello-Burri, S., Baldinger, R., Joset, P., Latal, B., Knirsch, W., Desai, S., Baumer, A., et al. (2013). Dosage changes of MED13L further delineate its role in congenital heart defects and intellectual disability. Eur. J. Hum. Genet. *21*, 1100–1104.
49. Gelb, B.D., Roberts, A.E., and Tartaglia, M. (2015). Cardiomyopathies in Noonan syndrome and the other RASopathies. Prog. Pediatr. Cardiol. *39*, 13–19.
50. Parenti, I., and Kaiser, F.J. (2021). Cornelia de Lange Syndrome as Paradigm of Chromatinopathies. Front. Neurosci. *15*, 774950.
51. Shen, M.M. (2007). Nodal signaling: developmental roles and regulation. Development *134*, 1023–1034.
52. Ryan, A.K., and Izpisúa Belmonte, J.C. (2000). Establishing a Left-Right Axis in the Embryo. IUBMB Life *50*, 1–11.
53. Namigai, E.K.O., Kenny, N.J., and Shimeld, S.M. (2014). Right across the tree of life: The evolution of left-right asymmetry in the Bilateria: Evolution of Left-Right Asymmetry in the Bilateria. Genesis *52*, 458–470.
54. Taira, M., Saint-Jeannet, J.-P., and Dawid, I.B. (1997). Role of the *Xlim-1* and *Xbra* genes in anteroposterior patterning of neural tissue by the head and trunk organizer. Proc. Natl. Acad. Sci. USA *94*, 895–900.
55. Deciphering Developmental Disorders Study (2015). Large-scale discovery of novel genetic causes of developmental disorders. Nature *519*, 223–228.

The American Journal of Human Genetics, Volume *109*

Supplemental information

Bi-allelic loss-of-function variants in *TMEM147* cause moderate to profound intellectual disability with facial dysmorphism and pseudo-Pelger-Huët anomaly

Quentin Thomas, Marialetizia Motta, Thierry Gautier, Maha S. Zaki, Andrea Ciolfi, Julien Paccaud, François Girodon, Odile Boespflug-Tanguy, Thomas Besnard, Jennifer Kerkhof, Haley McConkey, Aymeric Masson, Anne-Sophie Denommé-Pichon, Benjamin Cogné, Eva Trochu, Virginie Vignard, Fatima El It, Lance H. Rodan, Mohammad Ayman Alkhateeb, Rami Abou Jamra, Laurence Duplomb, Emilie Tisserant, Yannis Duffourd, Ange-Line Bruel, Adam Jackson, Siddharth Banka, Meriel McEntagart, Anand Saggar, Joseph G. Gleeson, David Sievert, Hyunwoo Bae, Beom Hee Lee, Kisang Kwon, Go Hun Seo, Hane Lee, Anjum Saeed, Nadeem Anjum, Huma Cheema, Salem Alawbathani, Imran Khan, Jorge Pinto-Basto, Joyce Teoh, Jasmine Wong, Umar Bin Mohamad Sahari, Henry Houlden, Kristina Zhelcheska, Melanie Pannetier, Mona A. Awad, Marion Lesieur-Sebellin, Giulia Barcia, Jeanne Amiel, Julian Delanne, Christophe Philippe, Laurence Faivre, Sylvie Odent, Aida Bertoli-Avella, Christel Thauvin, Bekim Sadikovic, Bruno Reversade, Reza Maroofian, Jérôme Govin, Marco Tartaglia, and Antonio Vitobello

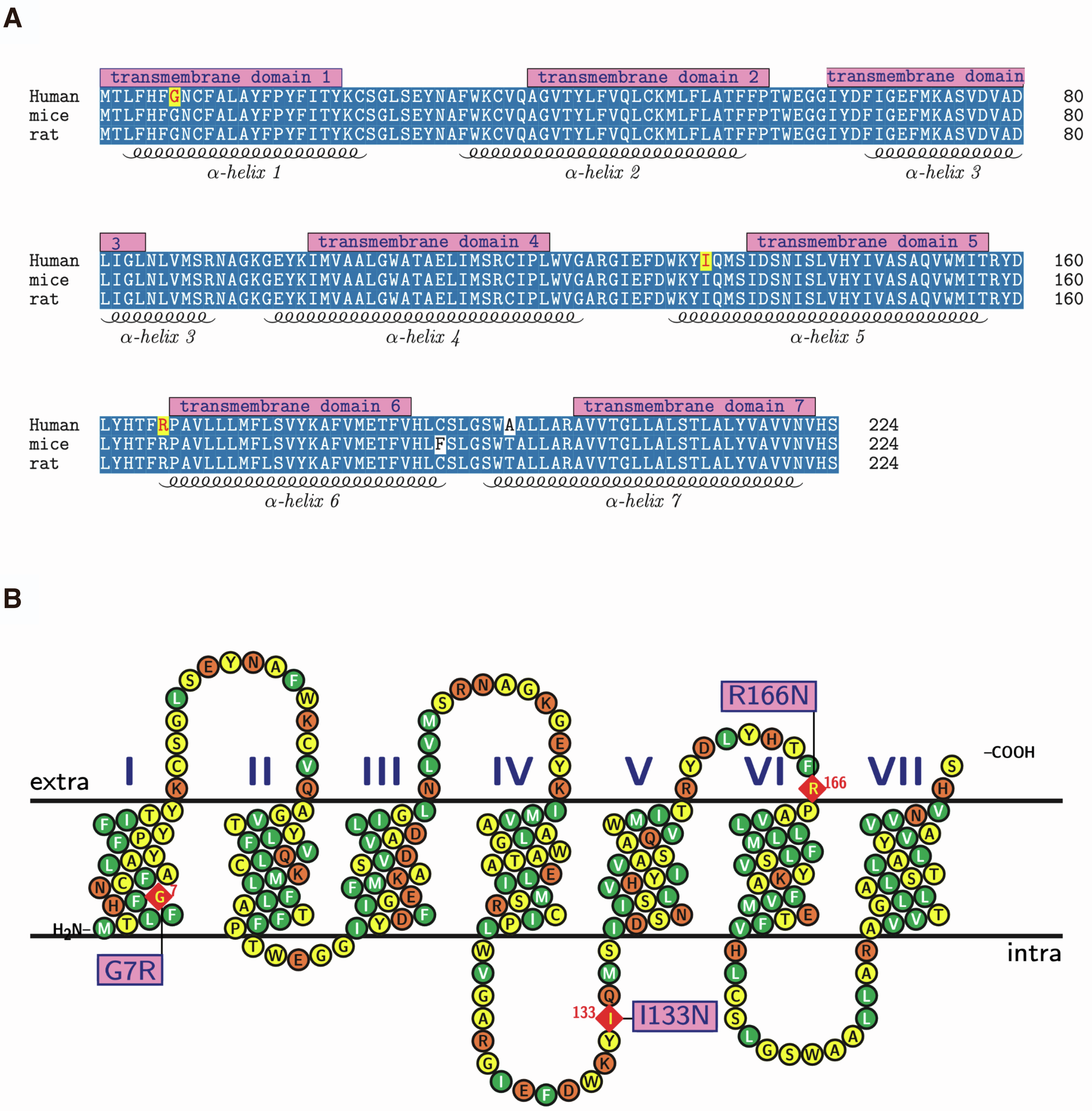
A
transmembrane domain 1
transmembrane domain 2
transmembrane domain
Human MTLFHFGNCFALAYFPYFITYKCSGLSEYNAFWKCVQAGVTYLFVQLCKMLFLATFFPTWEGGIYDFIGEFMKASVDVAD 80
mice MTLFHFGNCFALAYFPYFITYKCSGLSEYNAFWKCVQAGVTYLFVQLCKMLFLATFFPTWEGGIYDFIGEFMKASVDVAD 80
rat MTLFHFGNCFALAYFPYFITYKCSGLSEYNAFWKCVQAGVTYLFVQLCKMLFLATFFPTWEGGIYDFIGEFMKASVDVAD 80
α-helix 1
α-helix 2
α-helix 3
3
transmembrane domain 4
transmembrane domain 5
Human LIGLNLVMSRNAGKGEYKIMVAALGWATAELIMSRCIPLWVGARGIEFDWKYIQMSIDSNISLVHYIVASAQVWMITRYD 160
mice LIGLNLVMSRNAGKGEYKIMVAALGWATAELIMSRCIPLWVGARGIEFDWKYIQMSIDSNISLVHYIVASAQVWMITRYD 160
rat LIGLNLVMSRNAGKGEYKIMVAALGWATAELIMSRCIPLWVGARGIEFDWKYIQMSIDSNISLVHYIVASAQVWMITRYD 160
α-helix 3
α-helix 4
α-helix 5
transmembrane domain 6
transmembrane domain 7
Human LYHTFRPAVLLLMFLSVYKAFVMETFVHLCSLGSWAALLARAVVTGLLALSTLALYVAVVNVHS 224
mice LYHTFRPAVLLLMFLSVYKAFVMETFVHLFSLGSWTALLARAVVTGLLALSTLALYVAVVNVHS 224
rat LYHTFRPAVLLLMFLSVYKAFVMETFVHLCSLGSWTALLARAVVTGLLALSTLALYVAVVNVHS 224
α-helix 6
α-helix 7
B
extra
intra
I
II
III
IV
V
VI
VII
R166N
166
-COOH
H2N-
G7R
7
133
I133N

Figure S1 TMEM147 protein organization. (A) Clustal Ω alignment between the human, rat and mice sequences of TMEM147. The three sequences are 224 amino acid long and share above 99% identity. The seven transmembrane domains are indicated above the alignment and the α-helix regions are indicated below (from UniProt: Q9BVK8 and AlphaFold: AF-Q9VBK8-F1). Mutation positions are shown in red on a yellow background in the human sequence. The alignment was imaged with the TEXshade package in LaTeX.

(B) Membrane protein topology plot. The seven transmembrane domains are represented spaced by short loops. Mutation positions are indicated in pink boxes and the wild-type amino acid as a red diamond in the sequence string. Amino acids colored orange are considered external, green ones internal and yellow ambivalent in the structure. The plot was generated based on data found both in the UniProt and the AlphaFold databases using the TEXtopo package in LaTeX.

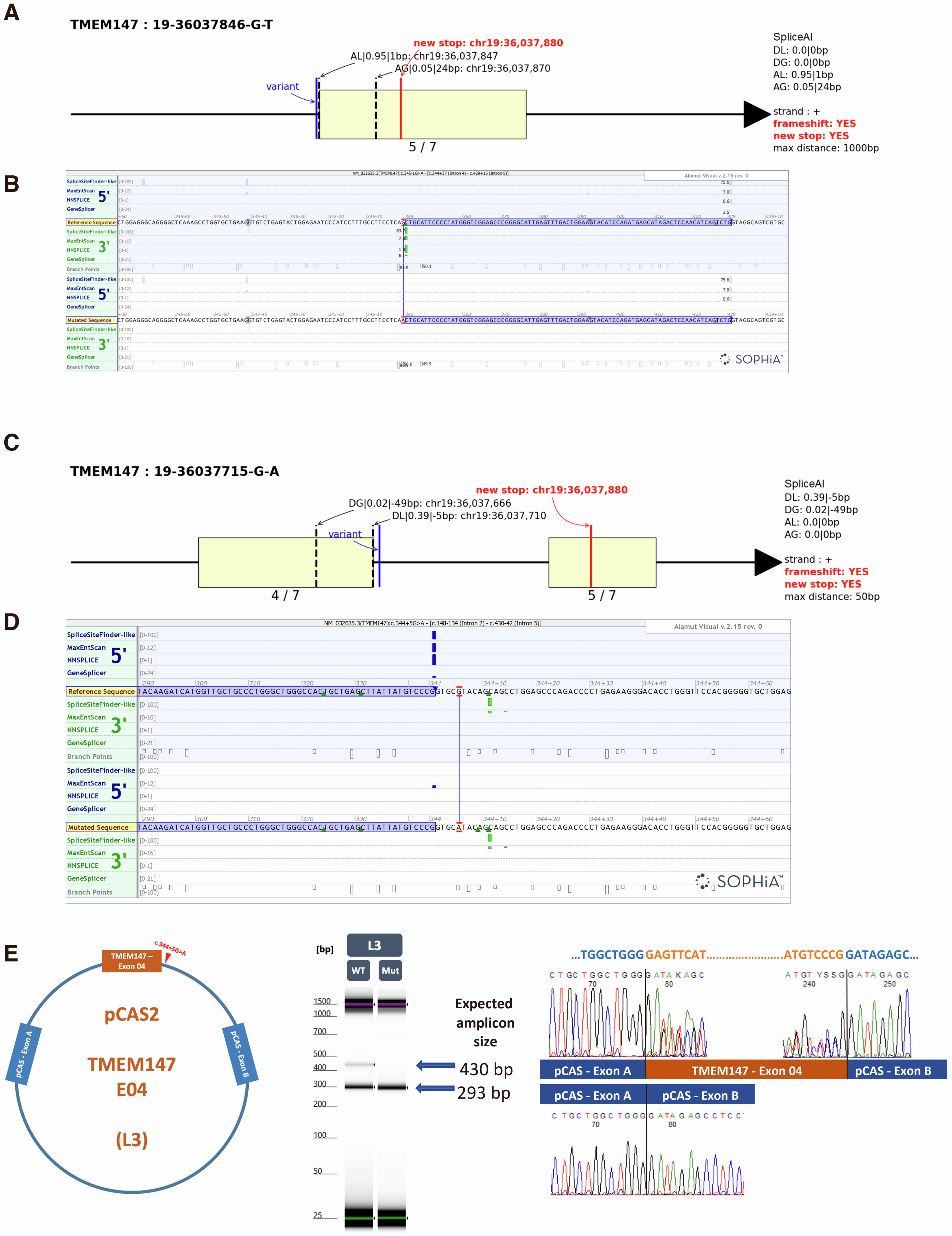

A
TMEM147 : 19-36037846-G-T
new stop: chr19:36,037,880
AL|0.95|1bp: chr19:36,037,847
AG|0.05|24bp: chr19:36,037,870
variant
5 / 7
SpliceAI
DL: 0.0|0bp
DG: 0.0|0bp
AL: 0.95|1bp
AG: 0.05|24bp
strand : +
frameshift: YES
new stop: YES
max distance: 1000bp
B
Reference Sequence
Mutated Sequence
SpliceSiteFinder-like
MaxEntScan
NNSPLICE
GeneSplicer
Branch Points
SOPHiA
C
TMEM147 : 19-36037715-G-A
new stop: chr19:36,037,880
DG|0.02|-49bp: chr19:36,037,666
DL|0.39|-5bp: chr19:36,037,710
variant
4 / 7
5 / 7
SpliceAI
DL: 0.39|-5bp
DG: 0.02|-49bp
AL: 0.0|0bp
AG: 0.0|0bp
strand : +
frameshift: YES
new stop: YES
max distance: 50bp
D
NM_032635.3(TMEM147):c.344+5G>A - [c.148-134 (Intron 2) - c.430-42 (Intron 5)]
Alamut Visual v.2.15 rev. 0
Reference Sequence
Mutated Sequence
SpliceSiteFinder-like
MaxEntScan
NNSPLICE
GeneSplicer
Branch Points
SOPHiA
E
TMEM147 – Exon 04
c.344+5G>A
pCAS2
TMEM147
E04
(L3)
pCAS - Exon A
pCAS - Exon B
[bp]
L3
WT
Mut
1500
1000
700
500
400
300
200
100
50
25
Expected amplicon size
430 bp
293 bp
...TGGCTGGG GAGTTCAT.....................ATGTCCCG GATAGAGC...
pCAS - Exon A
TMEM147 - Exon 04
pCAS - Exon B
pCAS - Exon A
pCAS - Exon B

Figure S2 Splice-site variants predictions and minigene results. SpliceAI score-based drawing (SpliceVI - 3 billion) and Alamut (Sophia Genetics) prediction of c.345-1G>T (A-B) and c.344+5G>A (C-D) splice-site variants. (E) Minigene analysis of the c.344+5G>A variant showing exon 4 skipping. The construct backbone (L3) utilized for this analysis is shown on the left. The red arrowhead indicates the position of the intronic variant. Reverse transcription polymerase chain reaction (RT-PCR) products obtained from wild-type (WT) and mutated (Mut) minigene constructs. The Sanger sequencing results of the amplicons obtained from the minigene analysis are shown on the right, confirming the inclusion of exon 4 in the WT construct and exon skipping in the Mut construct.

A

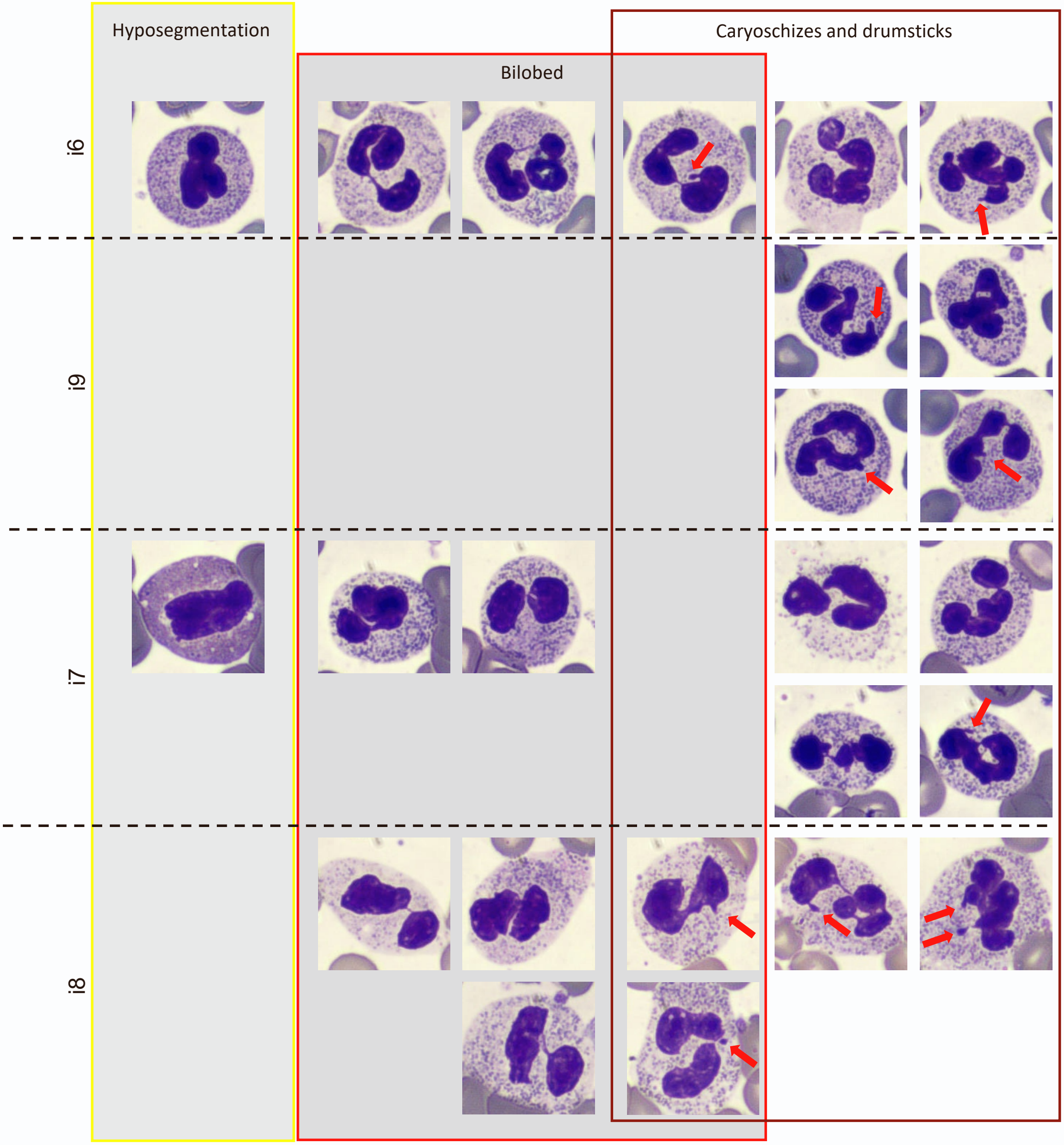
Hyposegmentation
Bilobed
Caryoschizes and drumsticks
i6
i9
i7
i8

B

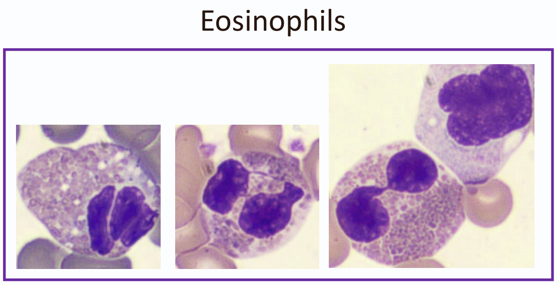
Eosinophils

Figure S3. Pseudo-Pelger-Huet anomaly identified in additional *TMEM147* individuals. (A) Chromatin clumping, hyposegmentation, bilobed nuclei, carioschizes, and drumsticks (red arrows) observed in neutrophils of i6, i7, i8, and i9 (F5-IV-2, F6-V-2, 5 and 6 respectively).(B) Sparse eosinophils showing nuclear anomalies observed in *TMEM147* individuals.

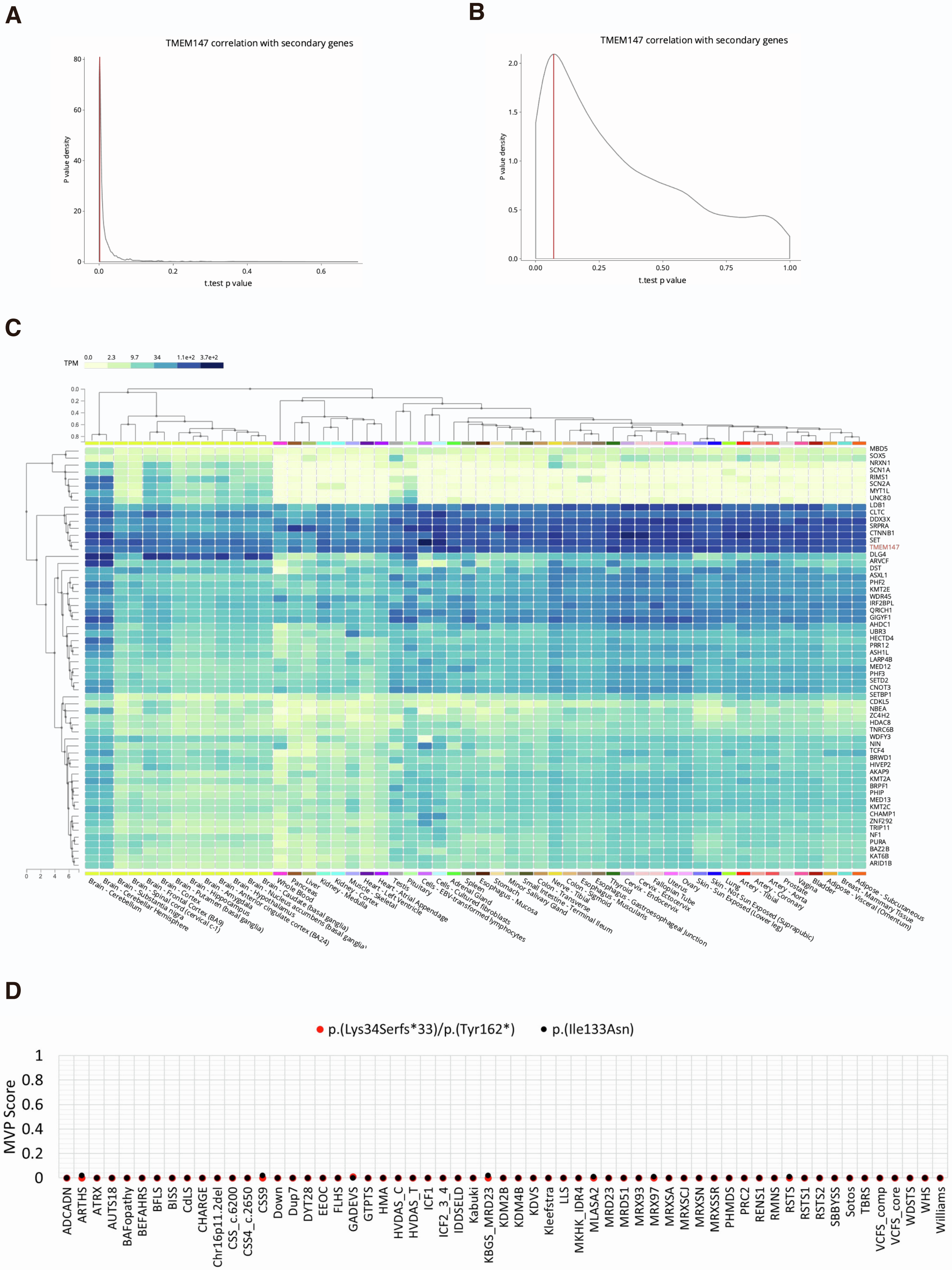
A
TMEM147 correlation with secondary genes
P value density
t.test p value
B
TMEM147 correlation with secondary genes
P value density
t.test p value
C
TPM
0.0 2.3 9.7 34 1.1e+2 3.7e+2
MBD5
SOX5
NRXN1
SCN1A
RIMS1
SCN2A
MYT1L
UNC80
LDB1
CLTC
DDX3X
SRPRA
CTNNB1
SET
TMEM147
DLG4
ARVCF
DST
ASXL1
PHF2
KMT2E
WDR45
IRF2BPL
QRICH1
GIGYF1
AHDC1
UBR3
HECTD4
PRR12
ASH1L
LARP4B
MED12
PHF3
SETD2
CNOT3
SETBP1
CDKL5
NBEA
ZC4H2
HDAC8
TNRC6B
WDFY3
NIN
TCF4
BRWD1
HIVEP2
AKAP9
KMT2A
BRPF1
PHIP
MED13
KMT2C
CHAMP1
ZNF292
TRIP11
NF1
PURA
BAZ2B
KAT6B
ARID1B
Brain - Cerebellum
Brain - Cerebellar Hemisphere
Brain - Substantia nigra
Brain - Spinal cord (cervical c-1)
Brain - Frontal Cortex (BA9)
Brain - Cortex
Brain - Putamen (basal ganglia)
Brain - Hippocampus
Brain - Amygdala
Brain - Hypothalamus
Brain - Anterior cingulate cortex (BA24)
Brain - Nucleus accumbens (basal ganglia)
Brain - Caudate (basal ganglia)
Whole Blood
Pancreas
Liver
Kidney - Medulla
Kidney - Cortex
Muscle - Skeletal
Heart - Left Ventricle
Heart - Atrial Appendage
Testis
Pituitary
Cells - EBV-transformed lymphocytes
Cells - Cultured fibroblasts
Adrenal Gland
Spleen
Esophagus - Mucosa
Stomach
Minor Salivary Gland
Small Intestine - Terminal Ileum
Colon - Transverse
Nerve - Tibial
Colon - Sigmoid
Esophagus - Muscularis
Thyroid
Esophagus - Gastroesophageal Junction
Cervix - Endocervix
Cervix - Ectocervix
Fallopian Tube
Uterus
Ovary
Skin - Sun Exposed (Lower leg)
Skin - Not Sun Exposed (Suprapubic)
Lung
Artery - Tibial
Artery - Coronary
Artery - Aorta
Prostate
Vagina
Bladder
Breast - Mammary Tissue
Adipose - Visceral (Omentum)
Adipose - Subcutaneous
D
p.(Lys34Serfs*33)/p.(Tyr162*)
p.(Ile133Asn)
MVP Score
ADCADN
ARTHS
ATRX
AUTS18
BAFopathy
BEFAHRS
BFLS
BISS
CdLS
CHARGE
Chr16p11.2del
CSS_c.6200
CSS4_c.2650
CSS9
Down
Dup7
DYT28
EEOC
FLHS
GADEVS
GTPTS
HMA
HVDAS_C
HVDAS_T
ICF1
ICF2_3_4
IDDSELD
Kabuki
KBGS_MRD23
KDM2B
KDM4B
KDVS
Kleefstra
LLS
MKHK_IDR4
MLASA2
MRD23
MRD51
MRX93
MRX97
MRXSA
MRXSCJ
MRXSN
MRXSSR
PHMDS
PRC2
RENS1
RMNS
RSTS
RSTS1
RSTS2
SBBYSS
Sotos
TBRS
VCFS_comp
VCFS_core
WDSTS
WHS
Williams

Figure S4 *TMEM147* co-expression analysis with neurodevelopmental genes and EpiSign (DNA methylation) analysis of peripheral blood from two cases with variants in *TMEM147*. *TMEM147* expression profile in different human tissues. (A) The plot shows the distribution of t-test *p*-values for Pearson correlations between *TMEM147* and NDD gene set in brain tissues as obtained by permutation testing. Summit *p*-value = 0.002 [**]. (B) The plot shows the distribution of t-test *p*-values for Pearson correlations between *TMEM147* and NDD genes in all tissues as obtained by permutation testing. Summit p-value = 0.071 [ns]. (C) Heatmap showing TMP (transcripts *per* kilobase million) values from GTEx database for *TMEM147* and its most correlated NDD genes (>|0.3|). Gene expression profiles are clustered by gene (vertical clustering) and by tissue (horizontal clustering). (D) A multi-class supervised classification system capable of discerning between multiple episignatures by generating a probability score (MVP) for each episignature. A positive score is typically greater than 0.5 and the presentation of all scores as 0.02 or less indicate a profile more similar to controls for each episignature assessed.

Table S1. Genetic variants identified in the *TMEM147* cohort

Individual Family	Genomic coordinates (GRCh37; hg19) chr19:	Transcript NM_032635.4	Protein	Familial segregation	GnomAD frequency	RASopathy or Chromatinopathy indication	*In silico* predictions
i1 – F1	g.36036812_36036830del<br>g.36038077C>G	c.100_118del<br>c.486C>G	p.Lys34Serfs*33<br>p.Tyr162*	Compound heterozygous	3.99e-6<br>0	RASopathy	
i2 – F2	g.36036704C>G<br>g.36037921dup	c.63C>G<br>c.419dup	p.Tyr21*<br>p.Asn140Lysfs*21	Compound heterozygous	0<br>0	none	
i3 – F3	g.36037900T>A	c.398T>A	p.Ile133Asn	homozygous	0	Chromatinopathy (Cornelia de Lange)	CADD 28<br>PP2 0.98<br>GERP 5.11<br>REVEL 0.472<br>3Cnet 0.859
i4 – F4	g.36037900T>A	c.398T>A	p.Ile133Asn	homozygous	0		CADD 28<br>PP2 0.98<br>GERP 5.11<br>REVEL 0.472<br>3Cnet 0.859
i5 – F4	g.36037900T>A	c.398T>A	p.Ile133Asn	homozygous	0		CADD 28<br>PP2 0.98<br>GERP 5.11<br>REVEL 0.472<br>3Cnet 0.859
i6 – F5	g.36037900T>A	c.398T>A	p.Ile133Asn	homozygous	0		CADD 28<br>PP2 0.98<br>GERP 5.11<br>REVEL 0.472<br>3Cnet 0.859
i7 – F6	g.36037900T>A	c.398T>A	p.Ile133Asn	homozygous	0		CADD 28<br>PP2 0.98<br>GERP 5.11<br>REVEL 0.472<br>3Cnet 0.859
i8 – F6	g.36037900T>A	c.398T>A	p.Ile133Asn	homozygous	0		CADD 28<br>PP2 0.98<br>GERP 5.11<br>REVEL 0.472<br>3Cnet 0.859
i9 – F6	g.36037900T>A	c.398T>A	p.Ile133Asn	homozygous	0		CADD 28<br>PP2 0.98<br>GERP 5.11<br>REVEL 0.472<br>3Cnet 0.859
i10 – F6	g.36037900T>A	c.398T>A	p.Ile133Asn	homozygous	0		CADD 28<br>PP2 0.98<br>GERP 5.11<br>REVEL 0.472<br>3Cnet 0.859
i11 – F7	g.36037900T>A	c.398T>A	p.Ile133Asn	homozygous	0		CADD 28<br>PP2 0.98<br>GERP 5.11<br>REVEL 0.472<br>3Cnet 0.859
i12 – F8	g.36037715G>A	c.344+5G>A	p.?	homozygous	1.41e-5		
i13 – F8	g.36037715G>A	c.344+5G>A	p.?	homozygous	1.41e-5		
i14 – F8	g.36037715G>A	c.344+5G>A	p.?	homozygous	1.41e-5		
i15 – F9	g.36037846G>T	c.345-1G>T	p.?	homozygous	0		
i16 – F10	g.36036660G>C	c.19G>C	p.Gly7Arg	homozygous	5.01e-5	RASopathy	CADD 27.9<br>PP2 1.0<br>GERP 5.4<br>REVEL 0.345<br>3Cnet 0.056
i17 – F10	g.36036660G>C	c.19G>C	p.Gly7Arg	homozygous	5.01e-5	RASopathy	CADD 27.9<br>PP2 1.0<br>GERP 5.4<br>REVEL 0.345<br>3Cnet 0.056

i18 – F11	g.36036660G>C	c.19G>C	p.Gly7Arg	homozygous	5.01e-5	none	CADD 27.9<br>PP2 1.0<br>GERP 5.4<br>REVEL 0.345<br>3Cnet 0.056
i19 – F12	g.36037443_36037452del	c.169_172del	p.Phe57Profs*15	homozygous	0		
i20 – F13	g.36037892G>A	c.390G>A	p.Trp130*	homozygous	0		
i21 – F14	g.36038131_36038134dup	c.540_543dup	p.Val182Leufs*66	homozygous	1.99e-5		
i22 – F15	g.36038087C>T	c.496C>T	p.Arg166Trp	homozygous	0		CADD 25.9<br>PP2 0.99<br>GERP 5.64<br>REVEL 0.206<br>3Cnet 0.471
i23 – F15	g.36038087C>T	c.496C>T	p.(Arg166Trp)	homozygous	0		CADD 25.9<br>PP2 0.99<br>GERP 5.64<br>REVEL 0.206<br>3Cnet 0.471

[a] PP2: PolyPhen2

[b] 3Cnet cutoff is 0.75

Table S2 Extended clinical features of the *TMEM147* cohort

Individual<br>Family	i1<br>F1	i2<br>F2	i3<br>F3	i4<br>4	i5<br>F4	i6<br>F5	i7<br>F6	i8<br>F6	i9<br>F6	i10<br>F6	i11<br>F7
Year of birth	2004	2008	2020	2005	2016	2016	2013	2019	2016	2018	2012
Sex	female	female	female	male	male	female	female	male	female	male	male
Measurements at birth	-1.12/+0.35/-1.23	UN	-6/-6/-5	-0.98/-1/-1.7	-0.5/+0.3/+05	-0.5/-0.08/-1.11	-4/-1.8/+0.1	-4.3/-1/-0.89	-3.2/-0.88/-1.4	-4/-1.3/-1.7	-1.9/-1.2/-1.5
Age at last follow-up	16y	8y	2y	13y	4y 3m	6y 6m	9y 1m	2y 11m	6y	3y 2m	2y 6m
Measurements at last follow-up	-1.5/+2.25/+1.5	-0.69/-0.5/UN	UN	-1.7/+1.3/+2.4	-2.1/+1/-1.2	-1/-1/-1.5	-0.8/-1.5/-2.3	-1.7/-1.8/-1.6	-1.2/-2.3/-1	-1.6/-1.6/-1.6	-1.8/-1/-4.6
Motor delay	+	+	+	+	+	+	+	+	+	+	+
Walking age	2y	5y	not acquired	4y	not acquired	3y	4y	not acquired	5y	not acquired	not acquired
Intellectual disability	+	+	UN	+	+	+	+	+	+	+	+
Severity	UN	severe	UN	IQ: 50	IQ: 45	IQ: 53	IQ: 48	IQ: 35-40	IQ: 45	IQ: 35	IQ: 20-25
Language delay	+	+	+	+	+	+	+	+	+	+	+
Speech ability upon last examination	single words	no speech	babbling	short sentences	no speech	single words	single words	no speech	single words	no speech	no speech
Behavioral problems	outbursts of anger, hyperactivity, self-injury	aggressivity, frustration intolerance self-injury	-	repetitive movements, outbursts of anger, hyperactivity	outbursts of anger, hyperactivity, anxiety	outbursts of anger, hyperactivity, hair-eating	outbursts of anger, hyperactivity	outbursts of anger, anxiety	outbursts of anger	outbursts of anger, anxiety	outbursts of anger, excessive crying
Neurological features	synkinesis with mirror movements of the hands (childhood), hypotonia	-	general hypotonia	-	-	-	-	-	-	bilateral severe sensorineural hearing loss	tonic seizures
Facial features	coarse facies, thick curly hair, hypertelorism, low-set ears, epicanthus large mouth, thick lower lip, sparse eyebrows, broad depressed nasal root	coarse facies, thin upper lip, epicanthus synophrys	broad depressed nasal root, synophrys, epicanthus, tented mouth	prominent forehead, broad nasal root, flat nasal tip, large tented mouth, long philtrum low set ears	prominent forehead, hypertelorism, down-slanting palpebral fissures, depressed broad nasal root, long philtrum, thin upper lip, everted lower lip, low set ears	coarse facies, elongated face, sparse hair, prominent forehead, depressed nasal root, down-slanting palpebral fissures, long philtrum, tented big mouth, low set ears	coarse facies, prominent forehead, sparse eyebrows, prominent nasal root, triangular prominent nose, long smooth philtrum, thin upper lip, low set large ears	coarse facies, prominent forehead, down-slanting palpebral fissures, synophrys depressed nasal root, long smooth philtrum, tented mouth, low set ears	coarse facies, prominent forehead depressed nasal root, broad nasal tip, long smooth philtrum, big mouth, low set ears	coarse facies, prominent forehead, curly hair, down-slanting palpebral fissures, depressed nasal root, long smooth philtrum, tented mouth, low set ears	prominent forehead, sparse eyebrows, depressed nasal root, broad nose tip, smooth philtrum, tented mouth, low set ears
Other Features	kyphosis, fifth finger bilateral clinodactyly, flat feet, valgus feet transverse palmar crease, pubic hair at 5 years 6 months	scoliosis, 5th finger clinodactyly, transverse palmar crease	transverse palmar crease	-	-	-	-	-	-	patent foramen ovale	-

Brain MRI	normal	normal	normal	thin corpus callosum	thin corpus callosum, myelination delay	thin corpus callosum, enlarged lateral ventricles, periventricular white matter T2 hyperintensities	thin corpus callosum, mild prominent verminan folia,	thin corpus callosum, mild prominent vermian folia	thin corpus callosum, enlarged lateral ventricles, prominent vermian folia	thin corpus callosum, enlarged lateral ventricles, white matter hyperintensities, prominent cerebellar folia	thin corpus callosum, hypoplastic lower vermis

Individual<br>Family	i12<br>F8	i13<br>F8	i14<br>F8	i15<br>F9	i16<br>F10	i17<br>F10	i18<br>F11	i19<br>F12	i20<br>F13	i21<br>F14	i22<br>F15	i23<br>F15
Year of birth	1996	2002	2004	2018	2013	2016	2004	2019	2016	2020	2006	2010
Sex	male	female	female	male	female	male	female	male	male	male	male	male
Measurements at birth	within norms	within norms	within norms	UN	within norms	within norms	UN	-1.17/-0.77/-0.9	-1/-0.8/-1.7	-1.17/+1.5/-1.8	-1/-0.5/+0.9	-1.2/-0.4/+1
Age at last follow-up	14y	8y	6y	3y	8y	5y	18y	2 years	4y6m	14m	6y	1y
Measurements at last follow-up	within norms	within norms	within norms	-1.2/0/-2.5	UN	-2/-2/UN	UN	+1/-1/+1	-3/-3/-3	-2/-1/+1	-2/-2/-4.6	-1.7/+0.2/-2.1
Motor delay	+	+	+	+	+	+	+	+	+	+	+	+
Walking age	3y	3y	3y	3y	UN	UN	3y	1.5y	not acquired	not acquired	not acquired	not acquired
Intellectual disability	+	+	+	+	+	+	+	+	+	+	+	too young
Severity	severe	severe	severe	severe	severe	severe	severe	moderate	severe	UN	IQ: 35	too young
Language delay	+	+	+	+	+	+	+	+	+	+	+	UN
Speech ability upon last examination	no speech	single words	no speech	single words	short sentences	single words	single words	single words	single words	babbling	no speech	UN
Behavioral problems	-	-	-	UN	outbursts of anger	outbursts of anger, self-injury	Anxiety, hyperactivity	-	outbursts of anger, aggressivity	-	outburst of anger, autistic traits	-
Neurological features	-	-	-	-	-	general hypotonia	-	-	-	-	tonic seizures	-
Facial features	coarse facies, synophrys, epicanthus, long smooth philtrum, elongated face	coarse facies, synophrys, epicanthus, broad nasal root, smooth philtrum, big mouth, thin upper lip	coarse facies, synophrys, epicanthus, broad nasal root, smooth philtrum big mouth	marked epicantus, large ears, tented mouth, short philtrum inverted nasal tip	coarse facies, hypertelorism, sparse hair, synophyrs, full lips	UN	long face, exophoria, prognathism, everted lower lip, anteverted floppy ears	coarse facies, prominent forehead epicanthus, thin upper lip	coarse facies, synophrys, marked epicanthus, flat nasal tip, long smooth philtrum big tented mouth, elongated face	coarse facies, prominent forehead, sparse eyebrows, epicanthus, depressed nasal root, long philtrum tented mouth	prominent forehead, depressed nasal root, broad nose tip, prominent nares, short philtrum, tented mouth, low set ears	prominent forehead, depressed nasal root, broad nose tip, prominent nares, long philtrum, tented mouth, low set ears
Other features	elbows arthrogryposis	ichthyosis	ichthyosis	atrial septal defect, large patent ductus arteriosus			kyphoscoliosis, pes planus, long and slender fingers and toes, late puberty	patent foramen ovale	-	-	-	-
Brain MRI	UN	UN	UN	normal	UN	UN	posterior white matter hyperintensity	reduced white matter volume	Periventricular and occipital white matter T2 hyperintensities, myelination delay	normal	thin corpus callosum mild frontoparietal cortical atrophy, wide interhemispheric fissure, bilateral mild deep sylvian fissure,	thin corpus callosum, mild frontoparietal cortical atrophy, wide interhemispheric fissure, bilateral mild deep sylvian fissure,

											periventricular and occipital white matter T2 hyperintensities	periventricular white matter T2 hyperintensities

[a] y: years
[b] m: months
[c] na: not acquired
[d] UN: unknown
[e] ty: too young
[f] se: severe
[g] mo moderate
[h] sw: single words
[i] ns: no speech
[j] ba: babbling
[k] ss: short sentences
[l] Measurements were expressed as standard deviation scores of weight, height and head circumference respectively

Supplemental material and methods

Protein modelling and *in silico* analysis

The ER translocon structure[1] was fetched from the Protein database (PDB id:6W6L) and displayed using PyMol version 2.5.2 (https://pymol.org/). All proteins were displayed as cartoon views. When necessary, surface was added to the polypeptide chains with the transparency set to 0.3. The PyMol Wizard tools was used to model the different mutations inside TMEM147 sequence. For each mutation studied, the WT amino acid was first visualized as sticks inside the cartoon representation, leaving only the side chain colored. Surface view was generated as mentioned above. Then the amino acid was mutated accordingly and made visible in the same manner. When steric clashes were reported for the possible rotamers by the wizard tool in PyMol, the least severe situation was selected. A putative view was modeled and oriented to match the WT situation. The alignment in Figure S1 was obtained with the TEXshade package in LaTeX.[2] The plot was generated based on data found both in the UniProt and the AlphaFold databases using the TEXtopo package in LaTeX.[3]

Reagents

Dulbecco's modified Eagle's medium (DMEM), fetal bovine serum (FBS), phosphate-buffered saline (PBS), glutamine and antibiotics were obtained from Euroclone (Wetherby, UK). Polyethylenimine (PEI) transfection reagent was purchased from Polysciences (Warrington, PA). Protease and phosphatase inhibitor cocktails, cycloheximide (CHX) and bafilomycin A1 were from Sigma-Aldrich (St. Luis, MO). Trans-Blot Turbo Transfer Packs were obtained from Bio-Rad Laboratories (Hercules, CA). ECL Western Blotting Detection reagents were from Pierce Biotechnology (Rockford, IL). QuiKChange II Site-Directed Mutagenesis kit was purchased from Stratagene (La Jolla, CA). pcDNA6.2/V5-HisA eukaryotic expression vector,

and Hoechst 33342 staining solution were from Invitrogen (Carlsbad, CA). The following antibodies were used: mouse monoclonal anti-V5 (R96025, Invitrogen); mouse monoclonal anti-GAPDH (SC-32233, Santa Cruz, Dallas, CA); rabbit polyclonal anti-calnexin (ab 10286, Abcam, Cambridge, UK); mouse monoclonal anti-CKAP4 (anti-CLIMP-63; ABS669-0100 Enzo Life Sciences, Farmingdale, NY, USA); rabbit polyclonal anti-RTN4 (anti-NOGO; ab47085 Abcam - Cambridge, UK); rabbit polyclonal anti-LBR (HPA062236 Sigma Aldrich, St. Louis, Missouri, USA); anti-actin horseradish peroxidase-conjugated anti-mouse (31450, Invitrogen); goat anti-mouse conjugated to Alexa Fluor 594 (A-11020, Invitrogen); goat anti-mouse conjugated to Alexa Fluor 568 (A-11031, Invitrogen) for fibroblast cell staining; goat anti-rabbit conjugated to Alexa Fluor 488 (A-11070, Invitrogen for COS-1 cell staining or A-11034, Invitrogen for fibroblast cell staining). Actin staining was obtained using ActinRed^{TM} 555 ReadyProbes^{TM} reagent (R37112, Invitrogen).

Constructs

The entire coding sequence of WT TMEM147 was cloned into the pcDNA6.2/V5-HisA eukaryotic expression vector. Mutant TMEM147 constructs carrying the p.Gly7Arg, p.Ile133Asn or p.Arg166Trp amino acid substitutions were generated by PCR-based site-directed mutagenesis using the QuiKChange II Site-Directed Mutagenesis Kit.[4] The identity of each construct was verified by bidirectional sequencing (ABI BigDye terminator Sequencing Kit v3.1, SeqStudio Genetic Analyzer; Applied Biosystems, Foster City, CA).

Cell culture, transfection, and inhibitor treatment

COS-1 cells were cultured in DMEM medium supplemented with 10% heat-inactivated FBS, 1% L-glutamine, and antibiotics (37 °C, humidified atmosphere containing 5% CO_2). Subconfluent cells were transfected using the PEI transfection reagent according to the

manufacturer's instructions. Cells were treated with cycloheximide (CHX) (10 µg/mL) or bafilomycin A1 (200 nM) to analyze protein stability and degradation.

Human primary fibroblasts (healthy control and patient-derived fibroblasts) were cultured in DMEM High Glucose medium 4.5 g/L (HyClone Thermo Scientific, Waltham, MA) supplemented with 10% Fetal Bovine Serum (FBS, Thermo Scientific) and 1% ZellShield (commercial mixture of antibiotics and anti-mycoplasmas reagents) (Minerva, Biovalley, France). Cells were cultured at 37 °C in a humidified 5% CO_2 atmosphere.

Cell homogenate and immunoblotting analyses

Cells were lysed in radio-immune precipitation assay (RIPA) buffer, pH 8.0, supplemented with protease and phosphatase inhibitors. Lysates were centrifuged at 16000g for 20 min at 4 °C, and supernatant protein concentration was determined by Bradford assay, using BSA as standard. Western blotting (WB) and densitometric analyses were performed as previously described.[4,5]

Immunohistochemistry and cell staining

Fibroblast cells were seeded 24 hours before labeling in 6-well plates containing coverslips, 2.10^5 cells per well. Cells were washed with cold PBS and fixed with 4% formaldehyde for 20 min at room temperature. The cells were then incubated with NH_4Cl (50mM) solution for 10 min, followed by permeabilization with 0.3% PBS-Triton solution for 7 min, still at room temperature. Saturation was performed with 1% PBS-BSA, 0.1% Tween 20, 15 min at room temperature. The primary antibody was incubated at 4°C overnight, diluted in blocking buffer. The next day, after 3 washes with cold PBS, the 1/1000th secondary antibody was added for 45 min. After 3 washes, nuclei were stained using Hoechst, washed and the mount is made on slides for microscope analysis.

Fibroblast cell lines were stained with May Grunwald-Giemsa (MGG) staining method to inspect nucleus morphology.

Imaging

COS-1 cells (15x10^3) were seeded on glass coverslips and transfected with the various constructs for 24 h. Cells were fixed with 4% paraformaldehyde (30 min, 4 °C) and permeabilized (0.5% Triton X-100, 10 min, room temperature [RT]). Cells were stained with a mouse monoclonal anti-V5 followed by goat anti-mouse Alexa Fluor 594, and then with a rabbit polyclonal anti-calnexin followed by goat anti-rabbit Alexa Fluor 488. Finally, nuclei were stained with Hoechst 33342 solution and glass coverslips were mounted on the microscope slides by using PBS-glycerol buffer. Confocal laser scanning microscopy analysis was performed by a Leica TCS-SP8X (Leica Microsystems) equipped with a 405 nm diode laser and a white light laser (WLL) source using excitation spectral laser lines at 488 nm and 594 nm. COS-1 cells stained only with the fluorochrome-conjugated secondary antibody were used to set up acquisition parameters. Signals from different fluorescent probes were taken in sequential scanning mode. Image processing used Adobe Photoshop 7.0 software (Adobe Systems Incorporated). Microscopic studies in patient-derived primary fibroblasts were performed at the GAD laboratory with a Zeiss AX10. Image analyses and quantifications were performed with ImageJ software. Quantification was obtained by calculating a Correlated Total Cell Fluorescence (CTCF) parameter with the following equation:

CTCF = integrated density – (area of selected cell x mean fluorescence of background).[6]

Blood smears were stained using standard May-Grünwald-Giemsa stain and then analyzed on a Sysmex XN haematology analyser coupled with a DI camera.

Statistical analysis

Statistical analyses were performed using GraphPad Prism software (GraphPad Software, La Jolla, CA) with a two-way ANOVA followed by Tukey's multiple comparison test or a one sample Wilcoxon test.

Transcriptome analysis

ARCHS4, a database with thousands of standardized RNA-Seq datasets (ARCHS4: https://maayanlab.cloud/archs4),[7] and *Correlation AnalyzeR* tool,[8] were used to calculate co-expression correlations with respect to tissue and disease (cancer/normal) condition. Pearson correlation between the *TMEM147* expression pattern and those from a previously defined set of genes mutated in neurodevelopmental disorders[9] in brain tissues was assessed by *Correlation AnalyzeR* R package. The selected gene set included genes with at least three *de novo bona fide* loss-of-function (LoF) mutations in unrelated individuals with NDDs and no *de novo* LoF mutation in apparently healthy subjects from the denovo-db v.1.6.1 database (denovo-db: https://denovo-db.gs.washington.edu/denovo-db/),[10] and nonredundant *de novo* mutations from Satterstrom et al. (2020).[11] Statistical significance of correlations was evaluated by permutation t-test comparing the selected NDD genes to a list of random genes of the same size. The *p*-value distribution constructed during permutation testing approximates the likelihood that *TMEM147* and the gene list are more correlated than expected by random association.[8] The GTEx database (GTEx: https://www.gtexportal.org/home/) was used to independently test the brain-specific correlation of *TMEM147* with the most correlated genes (>|0.3|) of the NDD gene set.

DNA Methylation Data Analysis

Methylation analysis was performed with version 3 of the clinically validated EpiSign assay as previously described.[12–15] Briefly, methylated and unmethylated signal intensity generated from the EPIC array was imported into R 3.5.1 for normalization, background correction and filtering. Beta values ranging from 0 (no methylation) to 1 (complete methylation) were calculated as a measure of methylation level and processed through the established support vector machine (SVM) classification algorithm for EpiSign disorders. The EpiSign Knowledge Database composed of over 5000 methylation profiles from reference disorder-specific and unaffected control cohorts was utilized by the classifier to generate disorder specific methylation variant pathogenicity (MVP) scores. MVP scores are a measure of prediction confidence for each disorder, ranging from 0 (discordant) to 1 (highly concordant). A positive classification typically generates MVP scores greater than 0.5 and these scores in combination with assessment of hierarchical clustering and multidimensional scaling are used in generating the final matched EpiSign result.

Supplemental references

1. McGilvray, P.T., Anghel, S.A., Sundaram, A., Zhong, F., Trnka, M.J., Fuller, J.R., Hu, H., Burlingame, A.L., and Keenan, R.J. (2020). An ER translocon for multi-pass membrane protein biogenesis. ELife *9*, e56889.

2. Beitz, E. (2000). TeXshade: shading and labeling of multiple sequence alignments using LaTeX2e. Bioinformatics *16*, 135–139.

3. Beitz, E. (2000). TeXshade: shading and labeling of multiple sequence alignments using LaTeX2e. Bioinformatics *16*, 135–139.

4. Motta, M., Fasano, G., Gredy, S., Brinkmann, J., Bonnard, A.A., Simsek-Kiper, P.O., Gulec, E.Y., Essaddam, L., Utine, G.E., Guarnetti Prandi, I., et al. (2021). SPRED2 loss-of-function causes a recessive Noonan syndrome-like phenotype. The American Journal of Human Genetics *108*, 2112–2129.

5. Motta, M., Pannone, L., Pantaleoni, F., Bocchinfuso, G., Radio, F.C., Cecchetti, S., Ciolfi, A., Di Rocco, M., Elting, M.W., Brilstra, E.H., et al. (2020). Enhanced MAPK1 Function Causes a

Neurodevelopmental Disorder within the RASopathy Clinical Spectrum. The American Journal of Human Genetics *107*, 499–513.

6. Abaza, M.S.I., Afzal, M., Al-Attiyah, R.J., and Guleri, R. (2016). Methylferulate from Tamarix aucheriana inhibits growth and enhances chemosensitivity of human colorectal cancer cells: possible mechanism of action. BMC Complement Altern Med *16*, 384.

7. Lachmann, A., Torre, D., Keenan, A.B., Jagodnik, K.M., Lee, H.J., Wang, L., Silverstein, M.C., and Ma'ayan, A. (2018). Massive mining of publicly available RNA-seq data from human and mouse. Nat Commun *9*, 1366.

8. Miller, H.E., and Bishop, A.J.R. (2021). Correlation AnalyzeR: functional predictions from gene co-expression correlations. BMC Bioinformatics *22*, 206.

9. Radio, F.C., Pang, K., Ciolfi, A., Levy, M.A., Hernández-García, A., Pedace, L., Pantaleoni, F., Liu, Z., de Boer, E., Jackson, A., et al. (2021). SPEN haploinsufficiency causes a neurodevelopmental disorder overlapping proximal 1p36 deletion syndrome with an episignature of X chromosomes in females. The American Journal of Human Genetics *108*, 502–516.

10. Turner, T.N., Yi, Q., Krumm, N., Huddleston, J., Hoekzema, K., F. Stessman, H.A., Doebley, A.-L., Bernier, R.A., Nickerson, D.A., and Eichler, E.E. (2017). denovo-db: a compendium of human *de novo* variants. Nucleic Acids Res *45*, D804–D811.

11. Satterstrom, F.K., Kosmicki, J.A., Wang, J., Breen, M.S., De Rubeis, S., An, J.-Y., Peng, M., Collins, R., Grove, J., Klei, L., et al. (2020). Large-Scale Exome Sequencing Study Implicates Both Developmental and Functional Changes in the Neurobiology of Autism. Cell *180*, 568-584.e23.

12. Levy, M.A., McConkey, H., Kerkhof, J., Barat-Houari, M., Bargiacchi, S., Biamino, E., Bralo, M.P., Cappuccio, G., Ciolfi, A., Clarke, A., et al. (2022). Novel diagnostic DNA methylation episignatures expand and refine the epigenetic landscapes of Mendelian disorders. Human Genetics and Genomics Advances *3*, 100075.

13. Aref-Eshghi, E., Kerkhof, J., Pedro, V.P., Barat-Houari, M., Ruiz-Pallares, N., Andrau, J.-C., Lacombe, D., Van-Gils, J., Fergelot, P., Dubourg, C., et al. (2020). Evaluation of DNA Methylation Episignatures for Diagnosis and Phenotype Correlations in 42 Mendelian Neurodevelopmental Disorders. The American Journal of Human Genetics *106*, 356–370.

14. Aref-Eshghi, E., Bend, E.G., Colaiacovo, S., Caudle, M., Chakrabarti, R., Napier, M., Brick, L., Brady, L., Carere, D.A., Levy, M.A., et al. (2019). Diagnostic Utility of Genome-wide DNA Methylation Testing in Genetically Unsolved Individuals with Suspected Hereditary Conditions. The American Journal of Human Genetics *104*, 685–700.

15. Sadikovic, B., Levy, M.A., Kerkhof, J., Aref-Eshghi, E., Schenkel, L., Stuart, A., McConkey, H., Henneman, P., Venema, A., Schwartz, C.E., et al. (2021). Clinical epigenomics: genome-wide DNA methylation analysis for the diagnosis of Mendelian disorders. Genetics in Medicine *23*, 1065–1074.
